# Supplementary material for: SIRT2-mediated ACSS2 K271 deacetylation suppresses lipogenesis under nutrient stress
Source: eLife. 2025 May 7;13:RP97019. doi: 10.7554/eLife.97019 (PMC12058118; doi:10.7554/eLife.97019)
Supplement: Figure 1—source data 1. [file elife-97019-fig1-data1.zip › Figure 1-source data 1/Figure 1-source data 1..pdf]

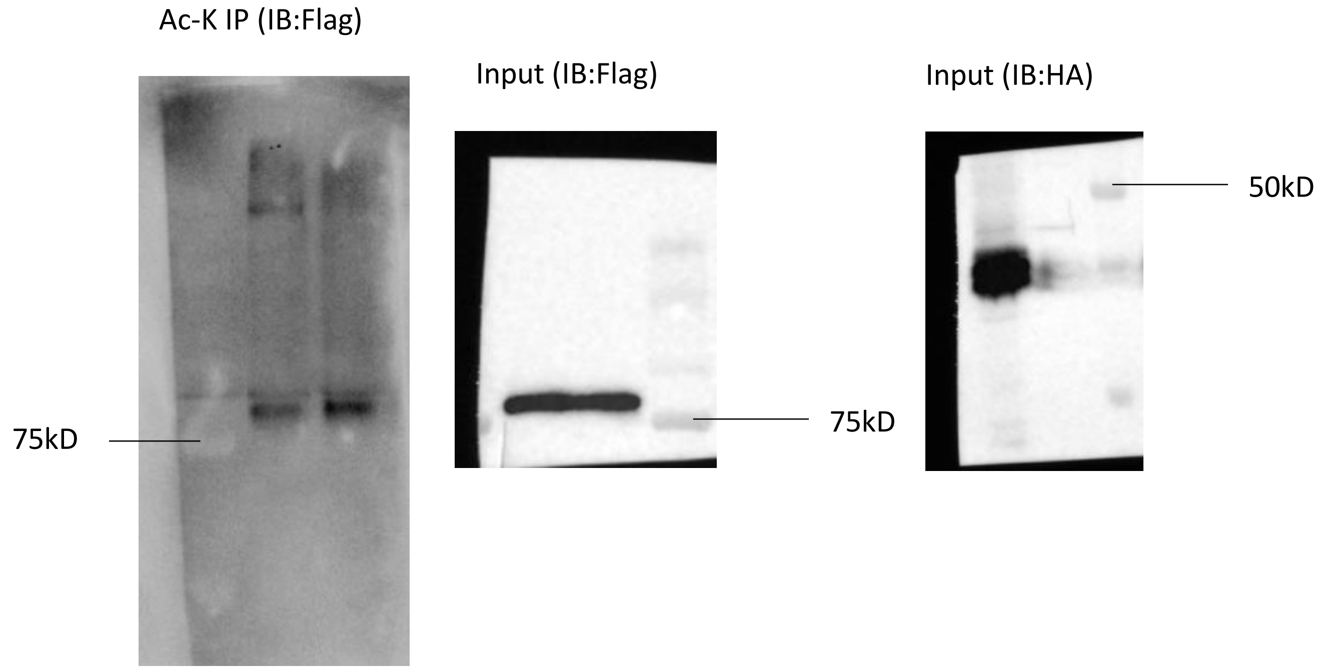

Figure 1, Source Data 1. Original membranes corresponding to Figure 1 A. BioRad protein ladder was used. Molecular weight for ACSS2 (78kD) and SIRT2 (43kD) are labelled.

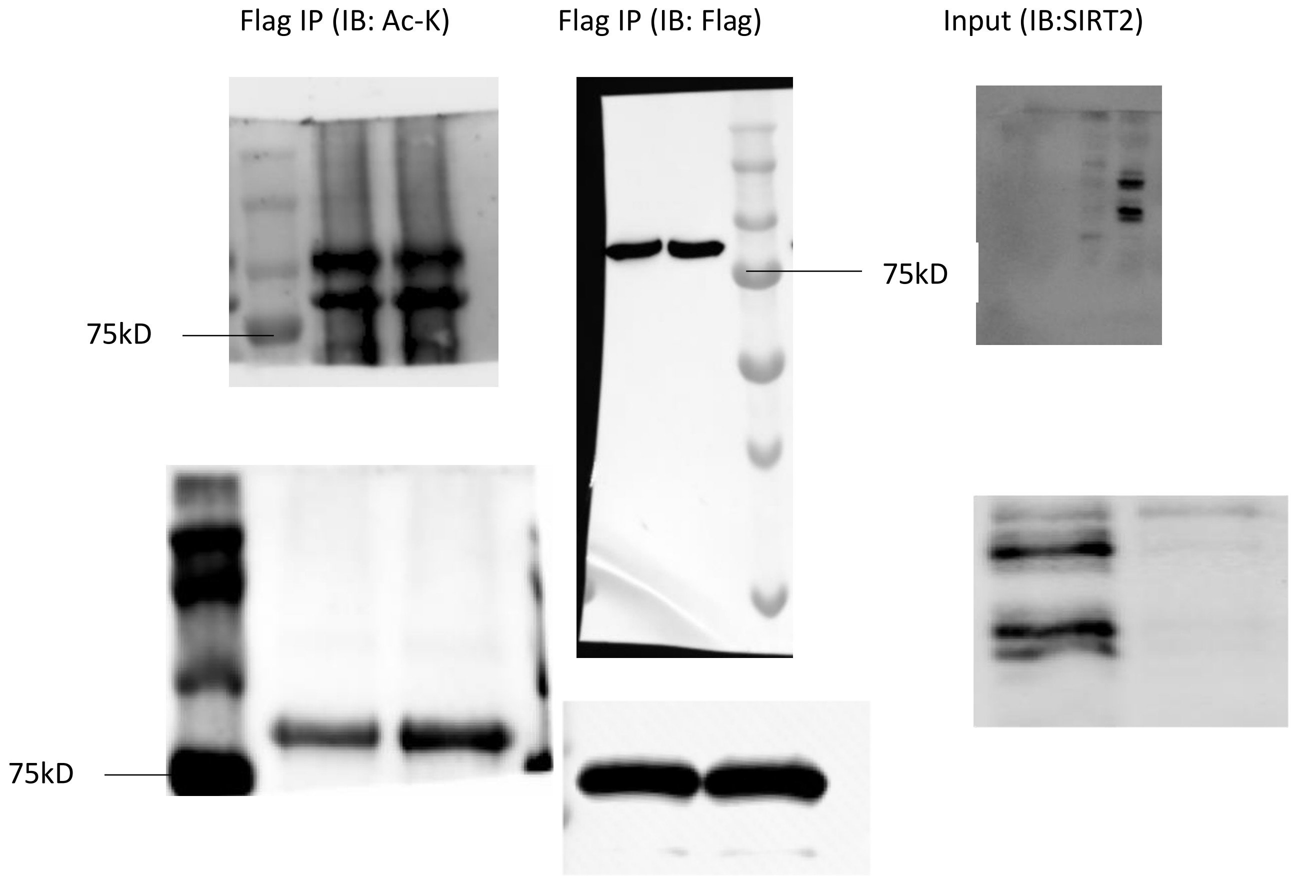

Figure 1, Source Data 1. Original membranes corresponding to Figure 1 B. BioRad protein ladder was used. Molecular weight for ACSS2 (78kD) is labelled.

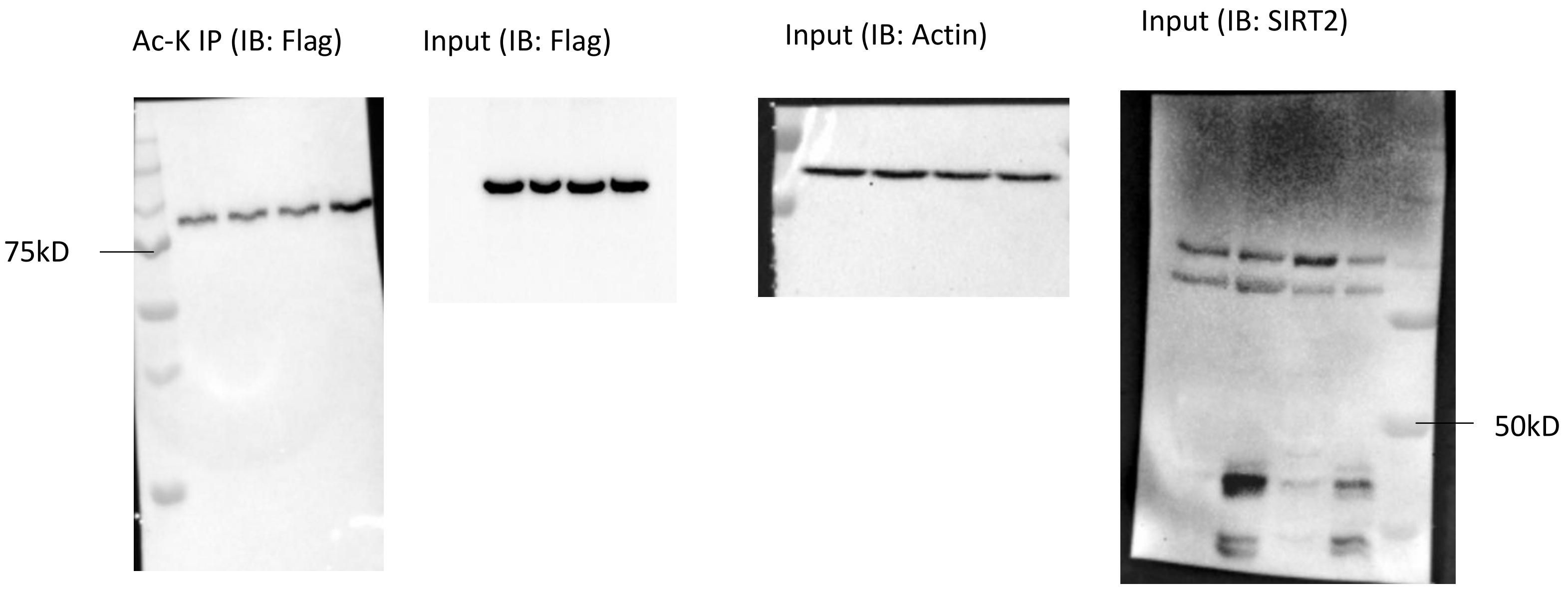

Figure 1, Source Data 1. Original membranes corresponding to Figure 1 C. BioRad protein ladder was used. Molecular weight for ACSS2 (78kD) and SIRT2 (43kD) are labelled.
