# Supplementary figures and images for: SIRT2-mediated ACSS2 K271 deacetylation suppresses lipogenesis under nutrient stress
Source: eLife. 2025 May 7;13:RP97019. doi: 10.7554/eLife.97019 (PMC12058118; doi:10.7554/eLife.97019)

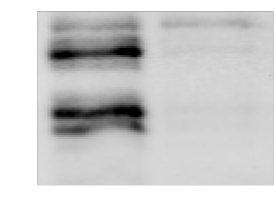

Supplement: Figure 1—source data 2. [file elife-97019-fig1-data2.zip › Figure 1-source data 2/1B/SIRT2_2.tiff]

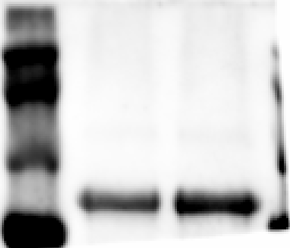

Supplement: Figure 1—source data 2. [file elife-97019-fig1-data2.zip › Figure 1-source data 2/1B/IP2.tiff]

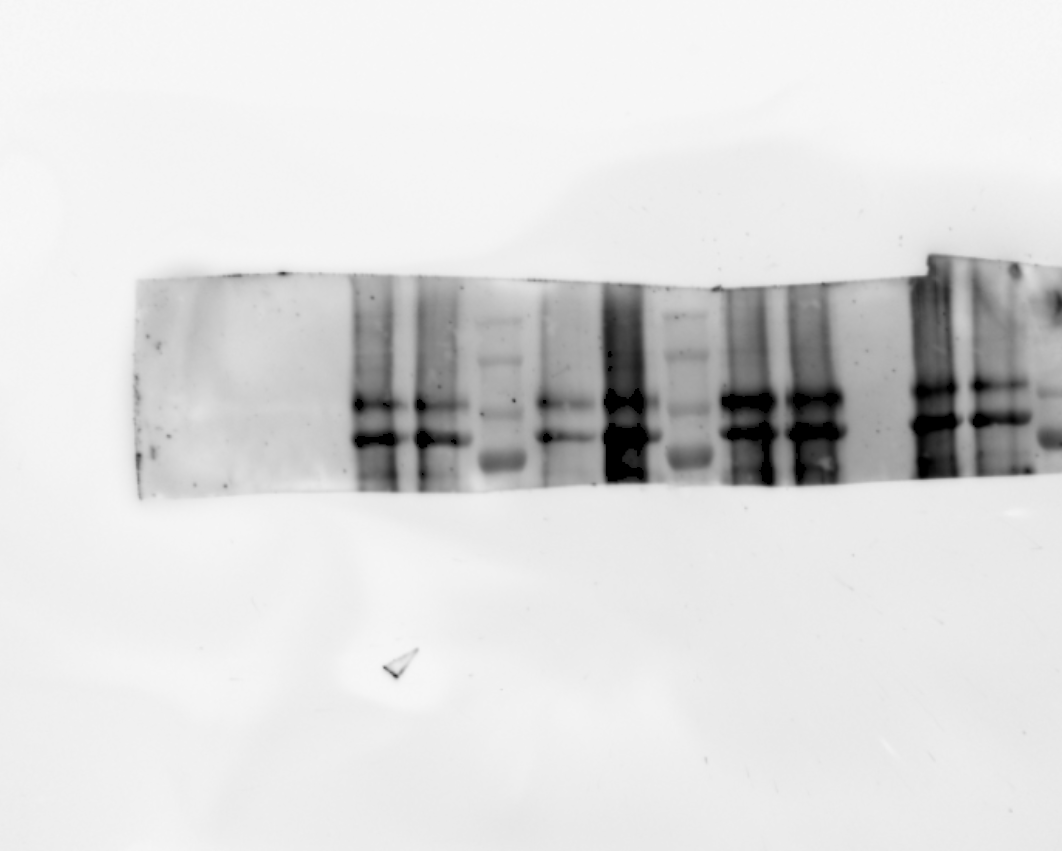

Supplement: Figure 1—source data 2. [file elife-97019-fig1-data2.zip › Figure 1-source data 2/1B/IP.tif]

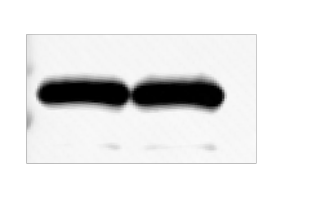

Supplement: Figure 1—source data 2. [file elife-97019-fig1-data2.zip › Figure 1-source data 2/1B/ACSS2_2.tiff]

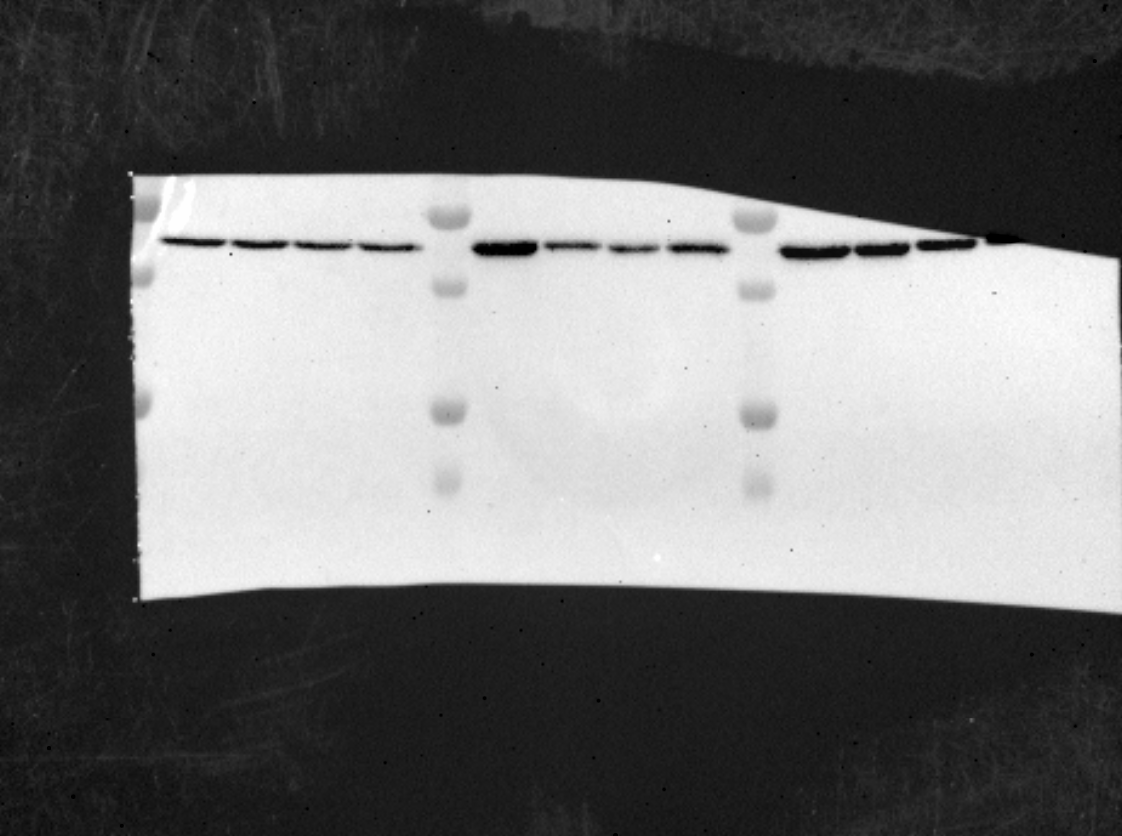

Supplement: Figure 1—source data 2. [file elife-97019-fig1-data2.zip › Figure 1-source data 2/1C/Actin.tif]

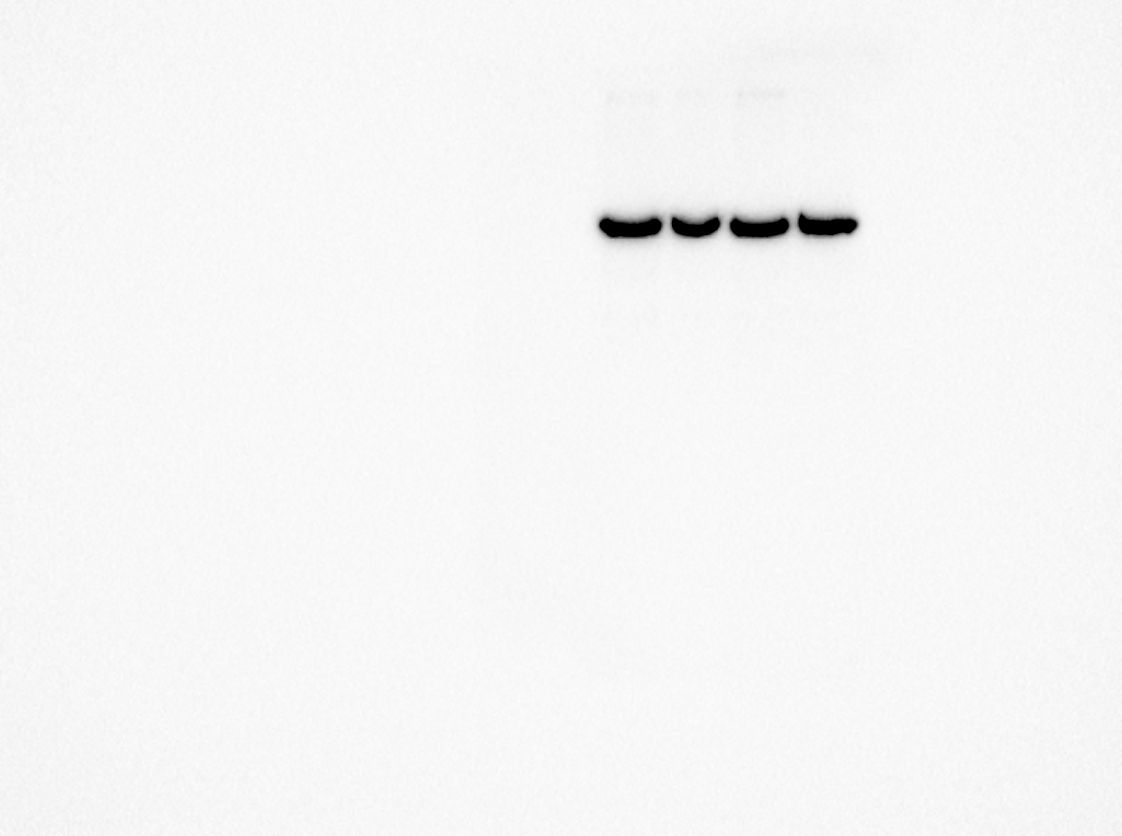

Supplement: Figure 1—source data 2. [file elife-97019-fig1-data2.zip › Figure 1-source data 2/1C/Flag.tif]

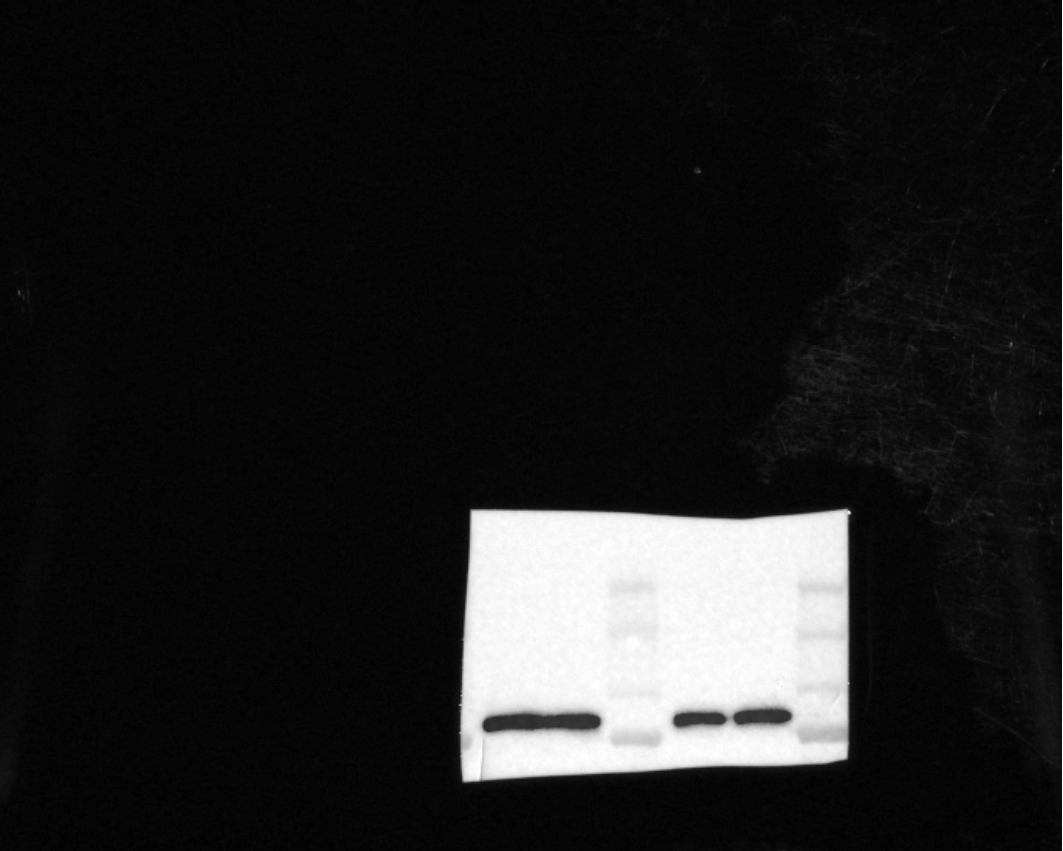

Supplement: Figure 1—source data 2. [file elife-97019-fig1-data2.zip › Figure 1-source data 2/1A/ACSS2.tif]

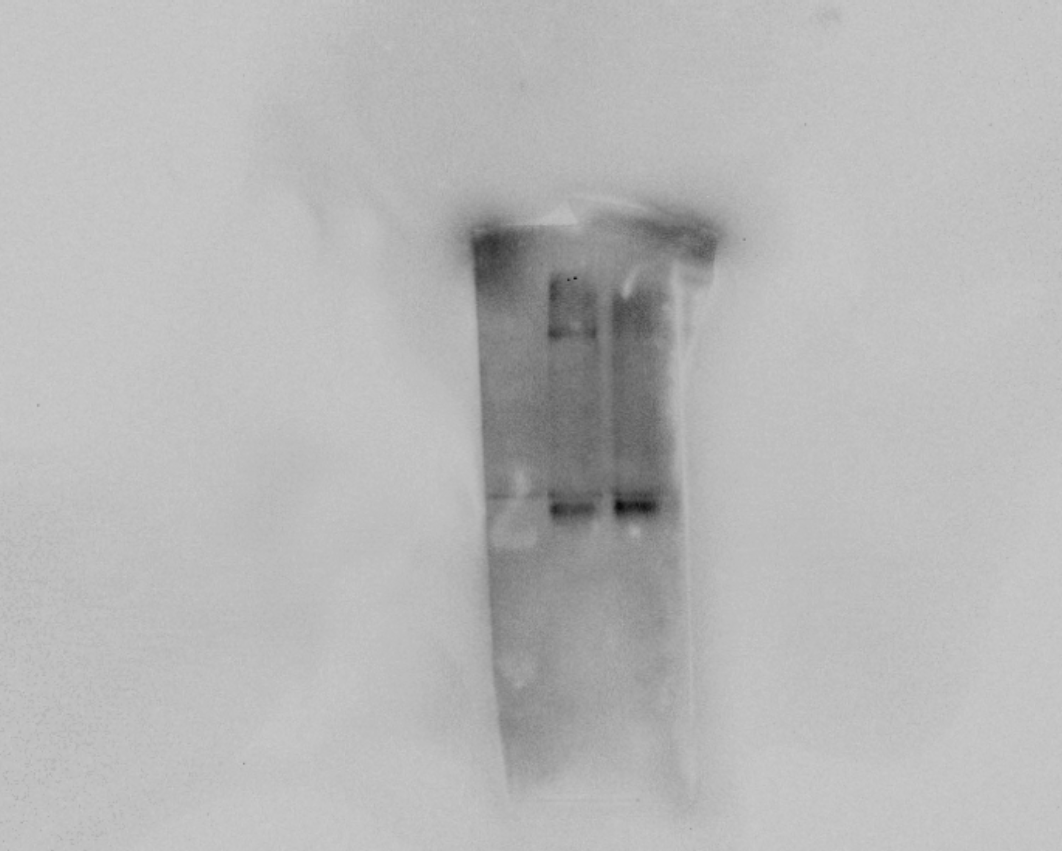

Supplement: Figure 1—source data 2. [file elife-97019-fig1-data2.zip › Figure 1-source data 2/1A/IP.tif]

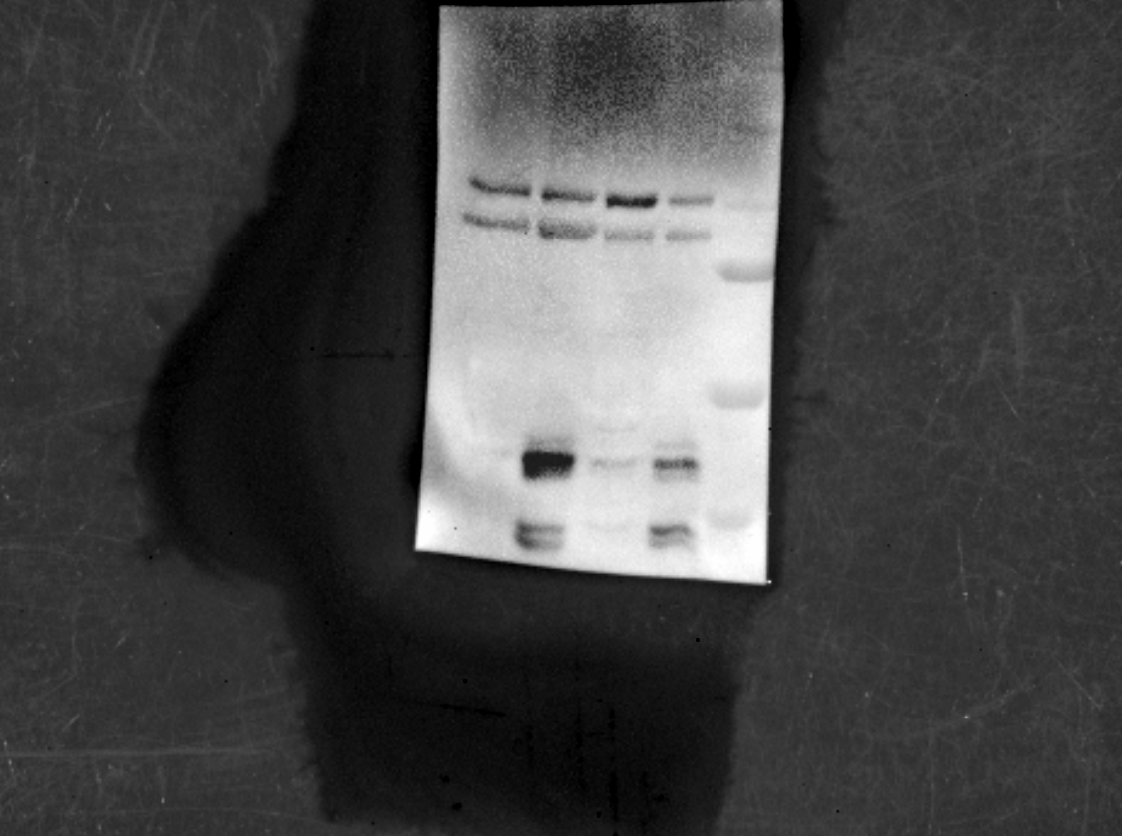

Supplement: Figure 1—source data 2. [file elife-97019-fig1-data2.zip › Figure 1-source data 2/1C/SIRT2.tif]

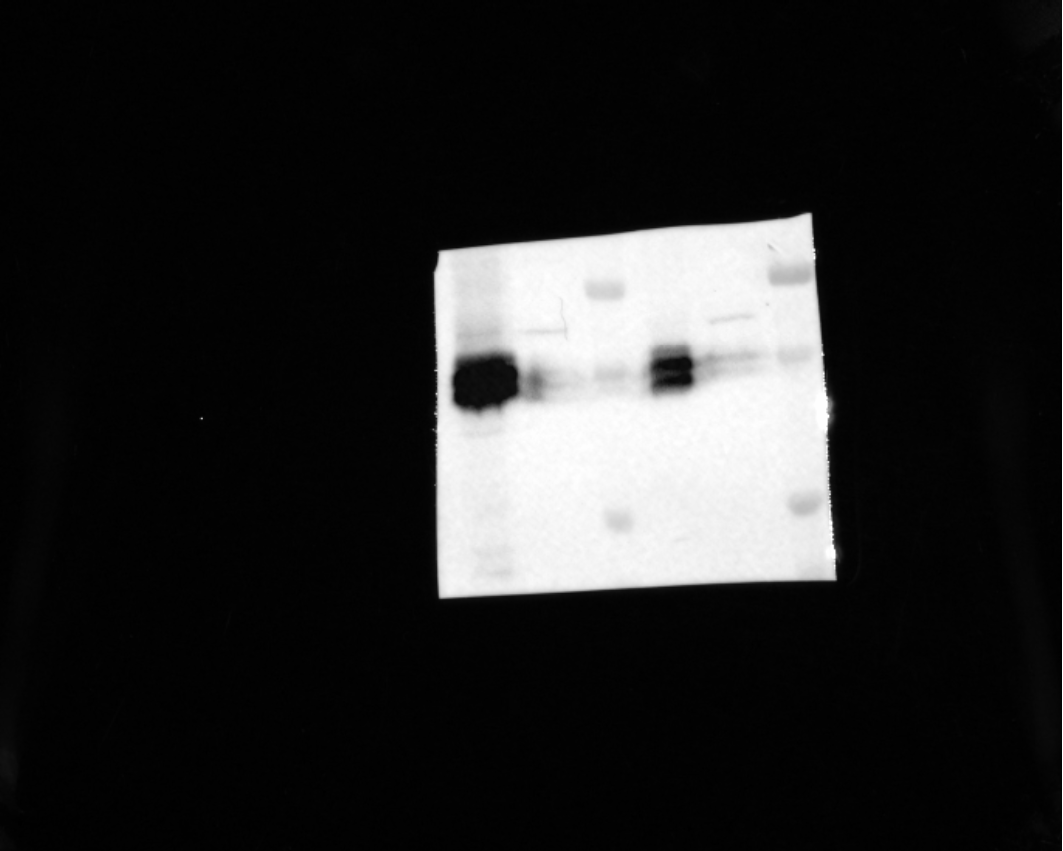

Supplement: Figure 1—source data 2. [file elife-97019-fig1-data2.zip › Figure 1-source data 2/1A/SIRT2.tif]

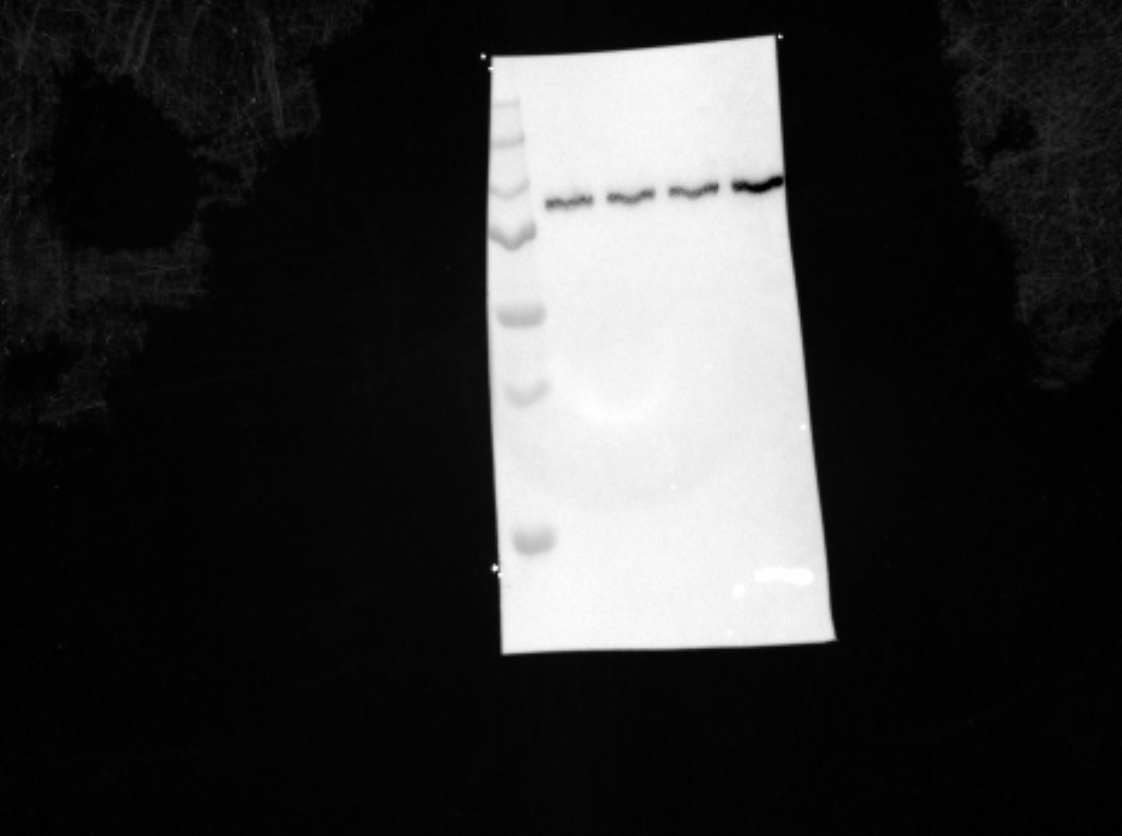

Supplement: Figure 1—source data 2. [file elife-97019-fig1-data2.zip › Figure 1-source data 2/1C/IP.tif]

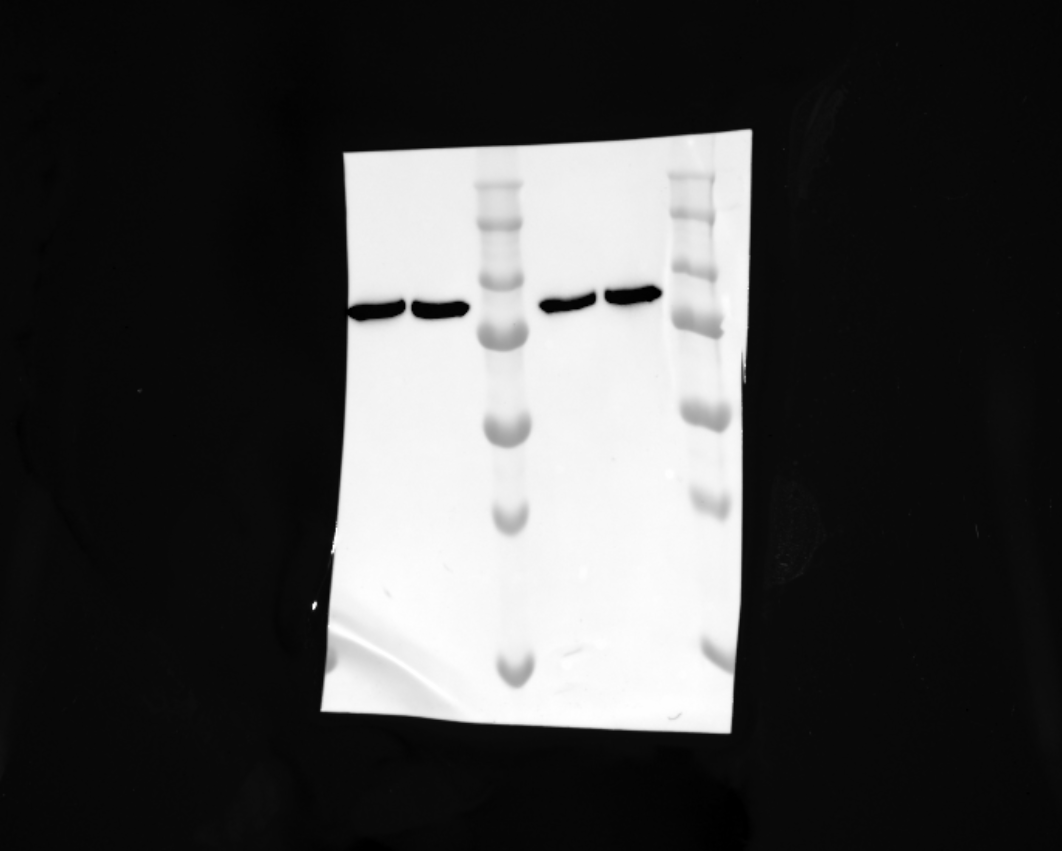

Supplement: Figure 1—source data 2. [file elife-97019-fig1-data2.zip › Figure 1-source data 2/1B/ACSS2.tif]

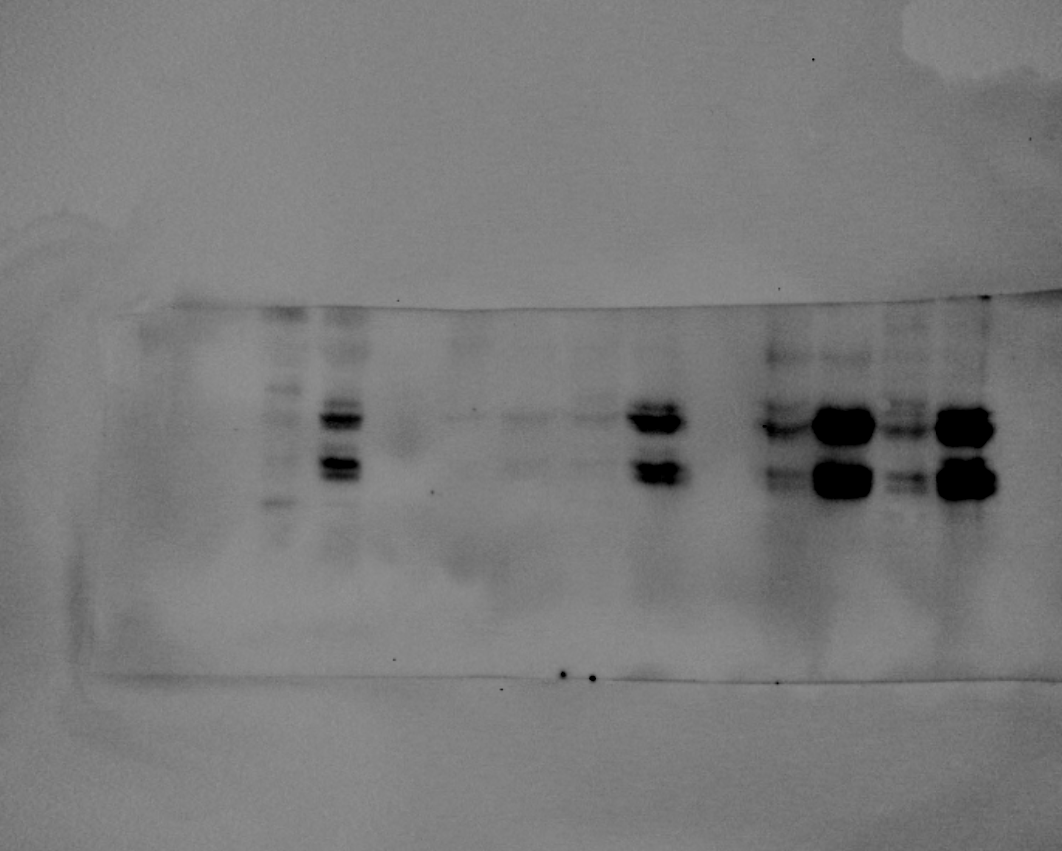

Supplement: Figure 1—source data 2. [file elife-97019-fig1-data2.zip › Figure 1-source data 2/1B/SIRT2.tif]

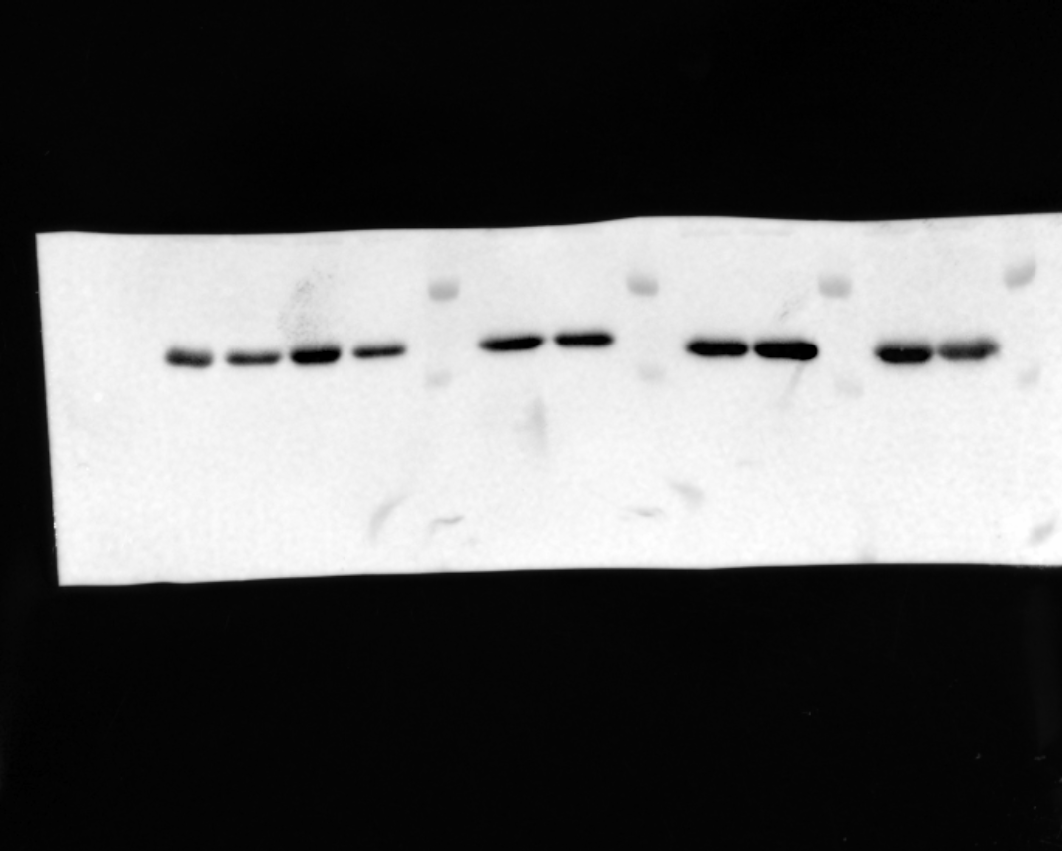

Supplement: Figure 1—figure supplement 1—source data 2. [file elife-97019-fig1-figsupp1-data2.zip › Figure 1-figure supplement 1, Source Data 2/Actin.tif]

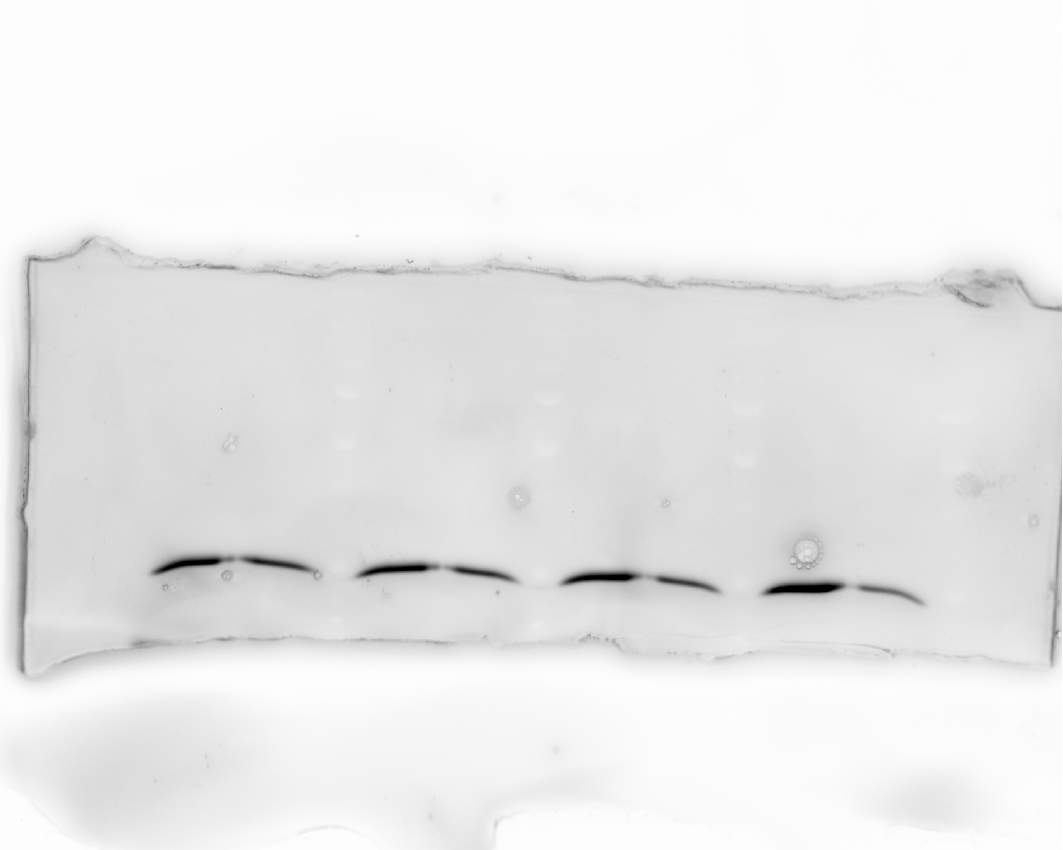

Supplement: Figure 1—figure supplement 1—source data 2. [file elife-97019-fig1-figsupp1-data2.zip › Figure 1-figure supplement 1, Source Data 2/Alkyne.tif]

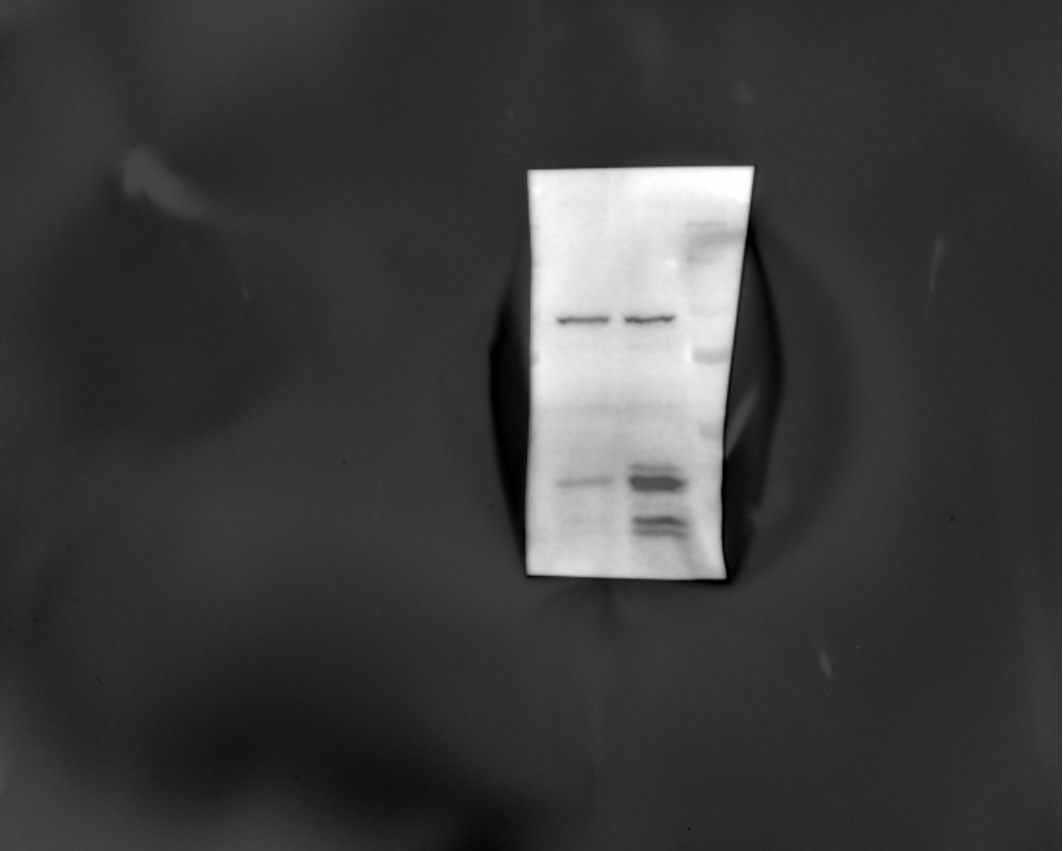

Supplement: Figure 1—figure supplement 1—source data 2. [file elife-97019-fig1-figsupp1-data2.zip › Figure 1-figure supplement 1, Source Data 2/ISIRT2.tif]

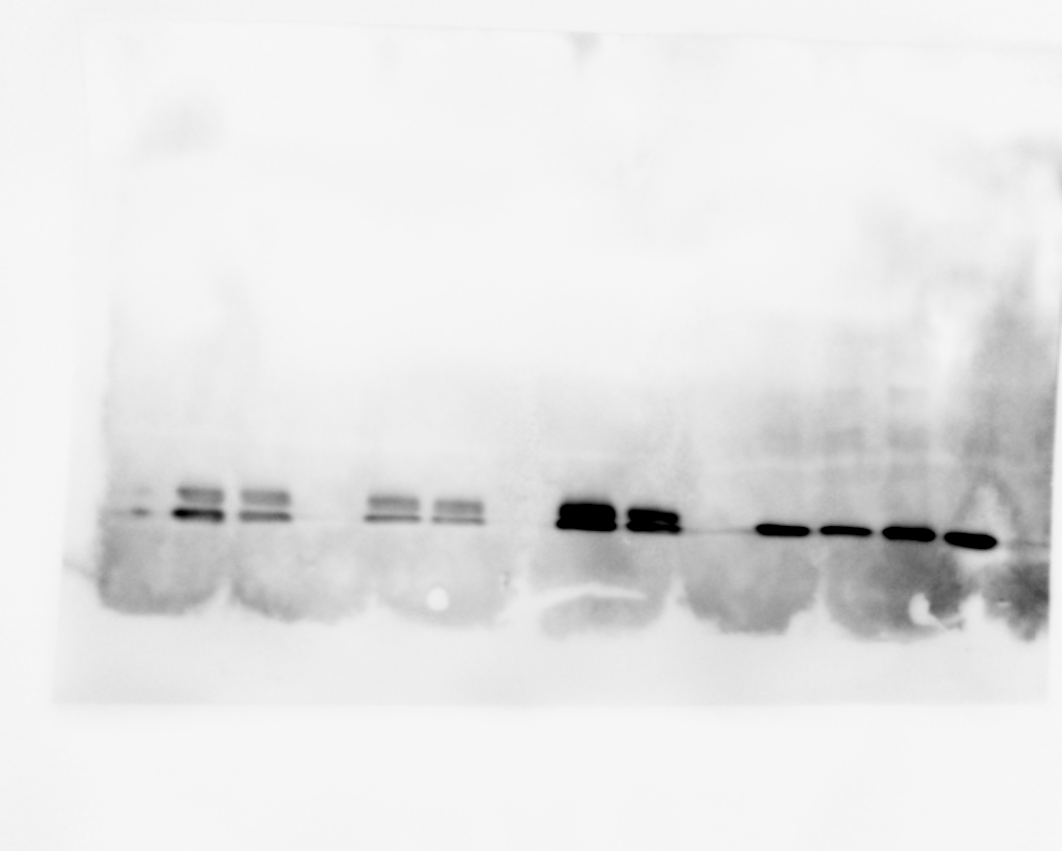

Supplement: Figure 1—figure supplement 1—source data 2. [file elife-97019-fig1-figsupp1-data2.zip › Figure 1-figure supplement 1, Source Data 2/Flag.tif]

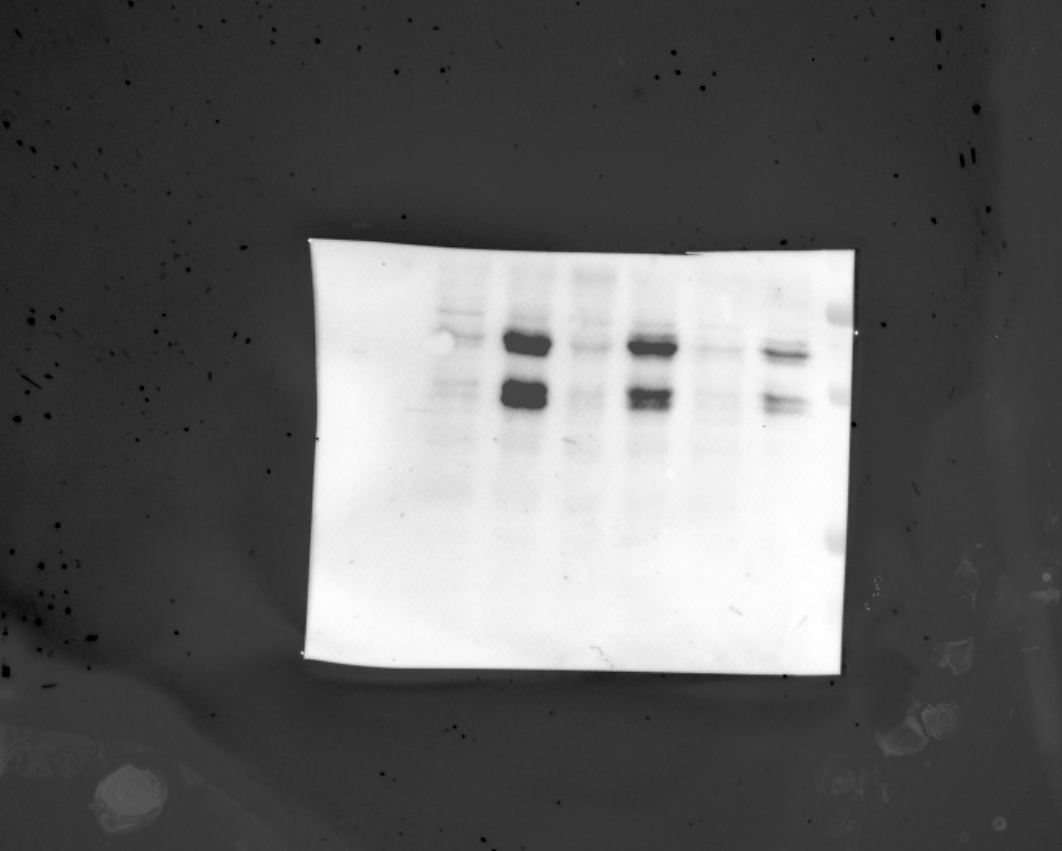

Supplement: Figure 1—figure supplement 2—source data 2. [file elife-97019-fig1-figsupp2-data2.zip › Figure 1-figure supplement 2, Source data 2/SIRT2.tif]

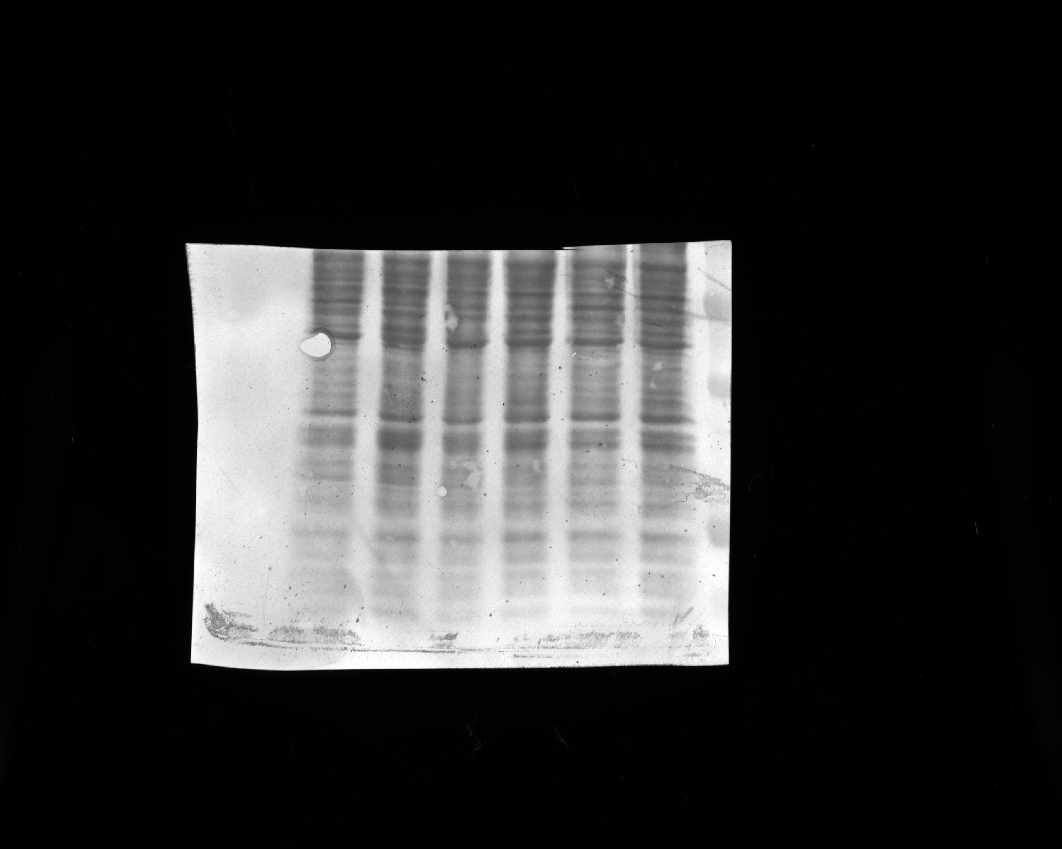

Supplement: Figure 1—figure supplement 2—source data 2. [file elife-97019-fig1-figsupp2-data2.zip › Figure 1-figure supplement 2, Source data 2/CBB.tif]

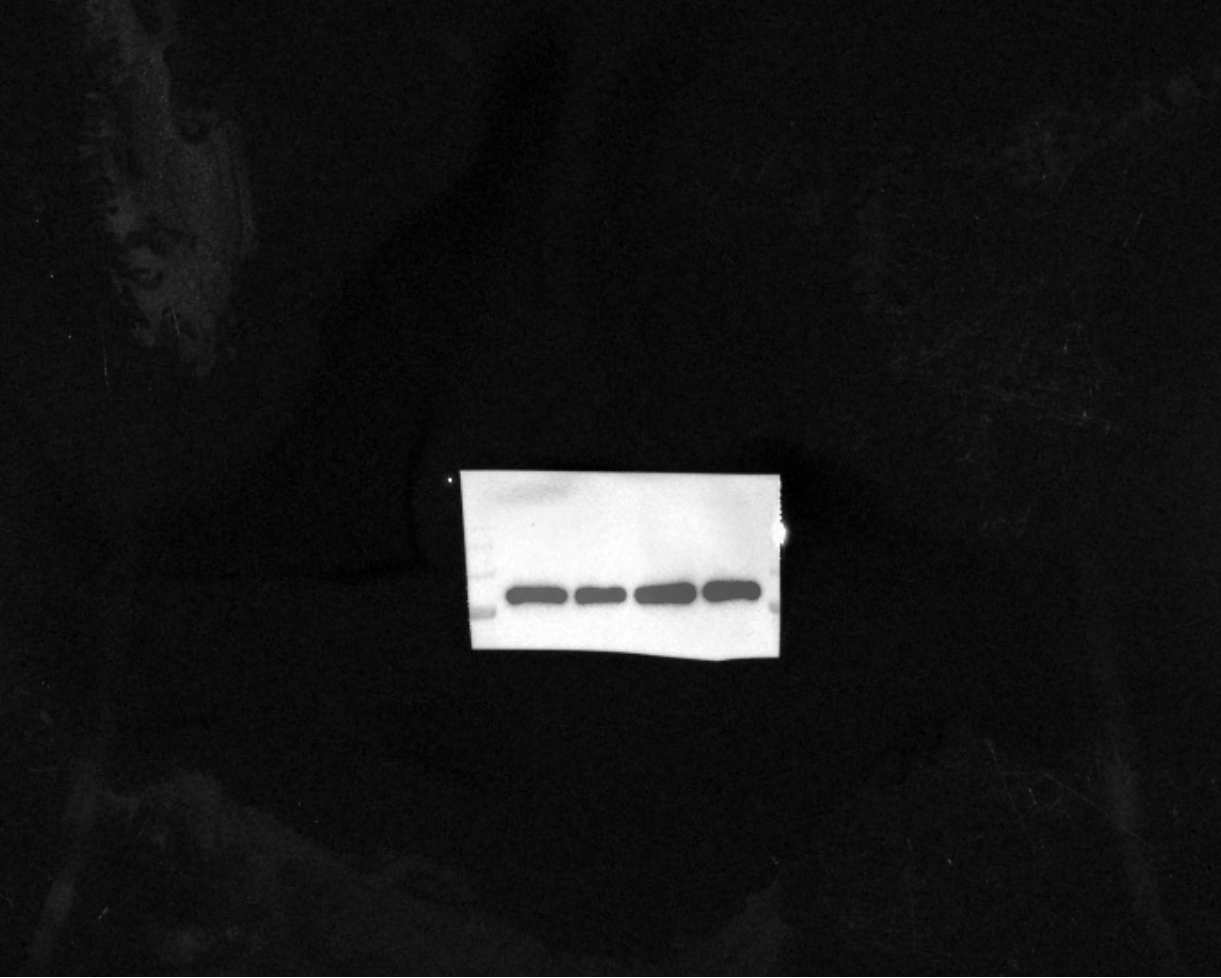

Supplement: Figure 1—figure supplement 3—source data 2. [file elife-97019-fig1-figsupp3-data2.zip › Figure 1-figure supplement 3, source data 2/Flag.tif]

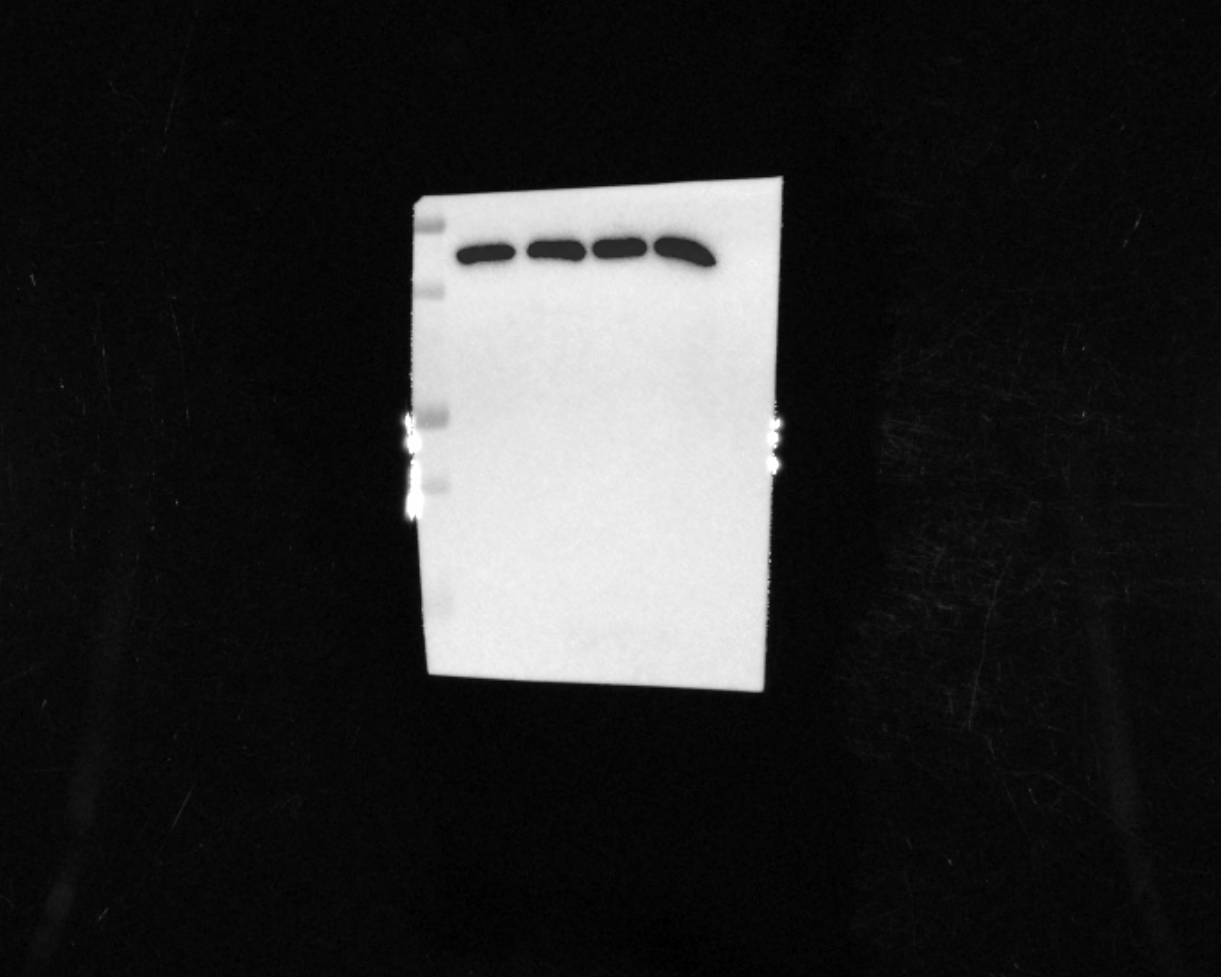

Supplement: Figure 1—figure supplement 3—source data 2. [file elife-97019-fig1-figsupp3-data2.zip › Figure 1-figure supplement 3, source data 2/Actin.tif]

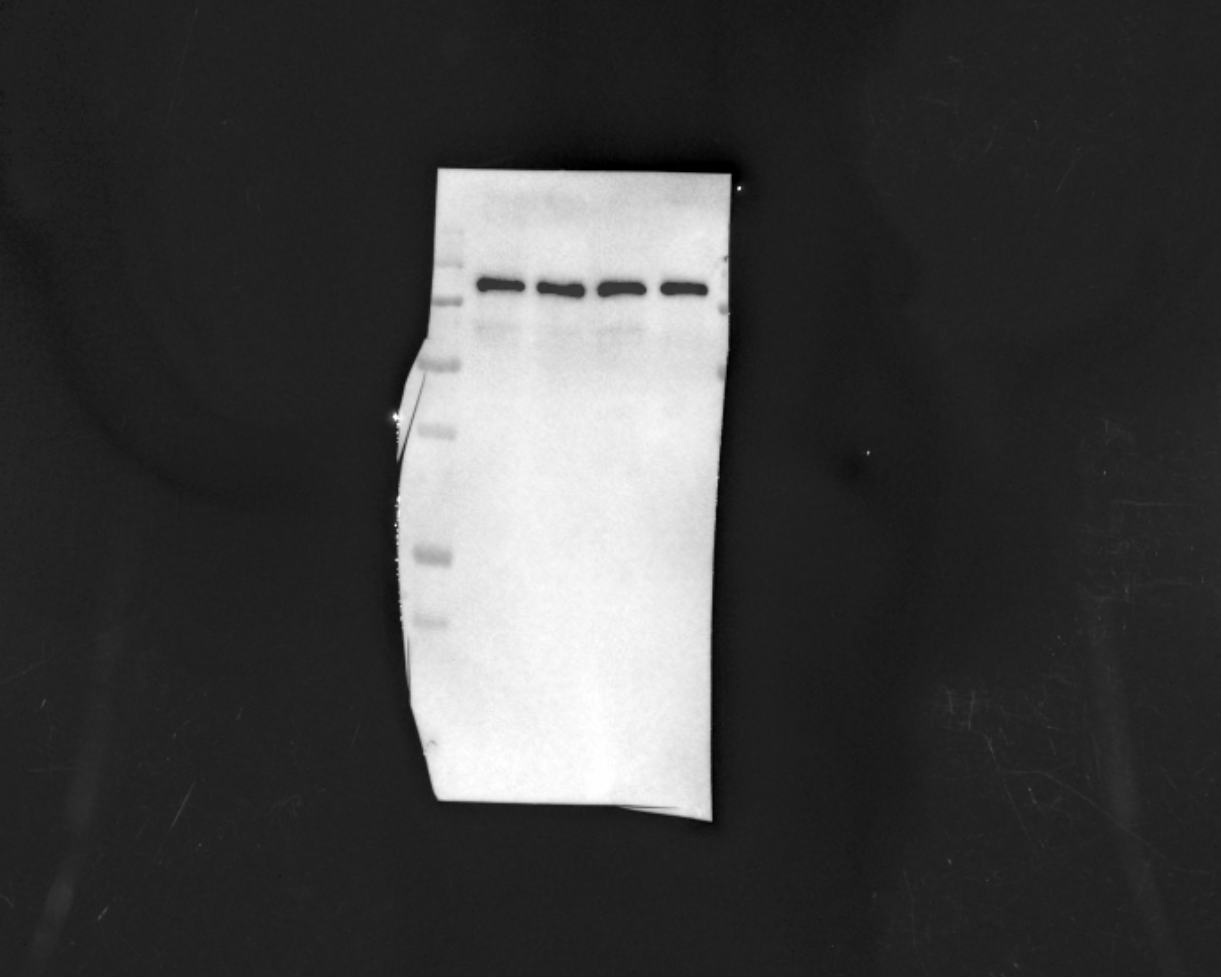

Supplement: Figure 1—figure supplement 3—source data 2. [file elife-97019-fig1-figsupp3-data2.zip › Figure 1-figure supplement 3, source data 2/IP.tif]

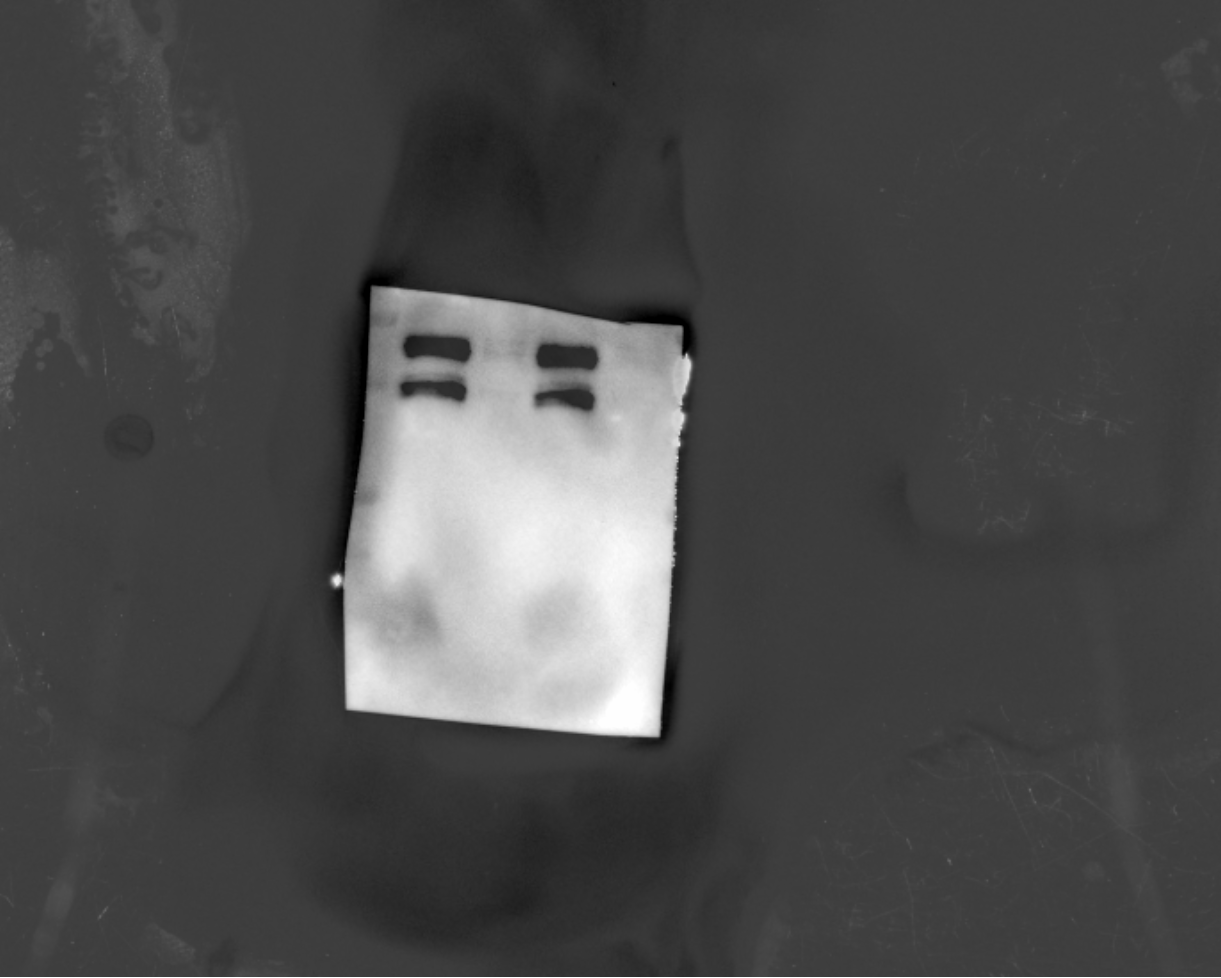

Supplement: Figure 1—figure supplement 3—source data 2. [file elife-97019-fig1-figsupp3-data2.zip › Figure 1-figure supplement 3, source data 2/SIRT2.tif]

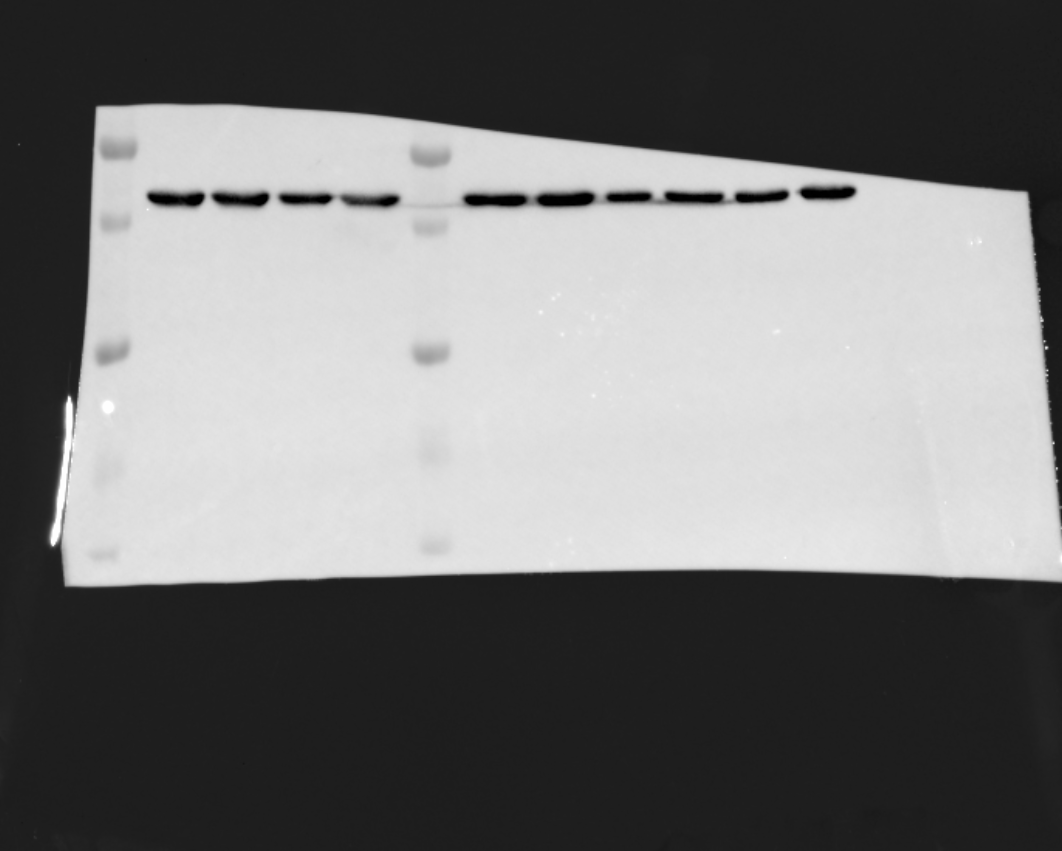

Supplement: Figure 1—figure supplement 4—source data 2. [file elife-97019-fig1-figsupp4-data2.zip › Figure 1-figure supplement 4, source data 2/Actin.tif]

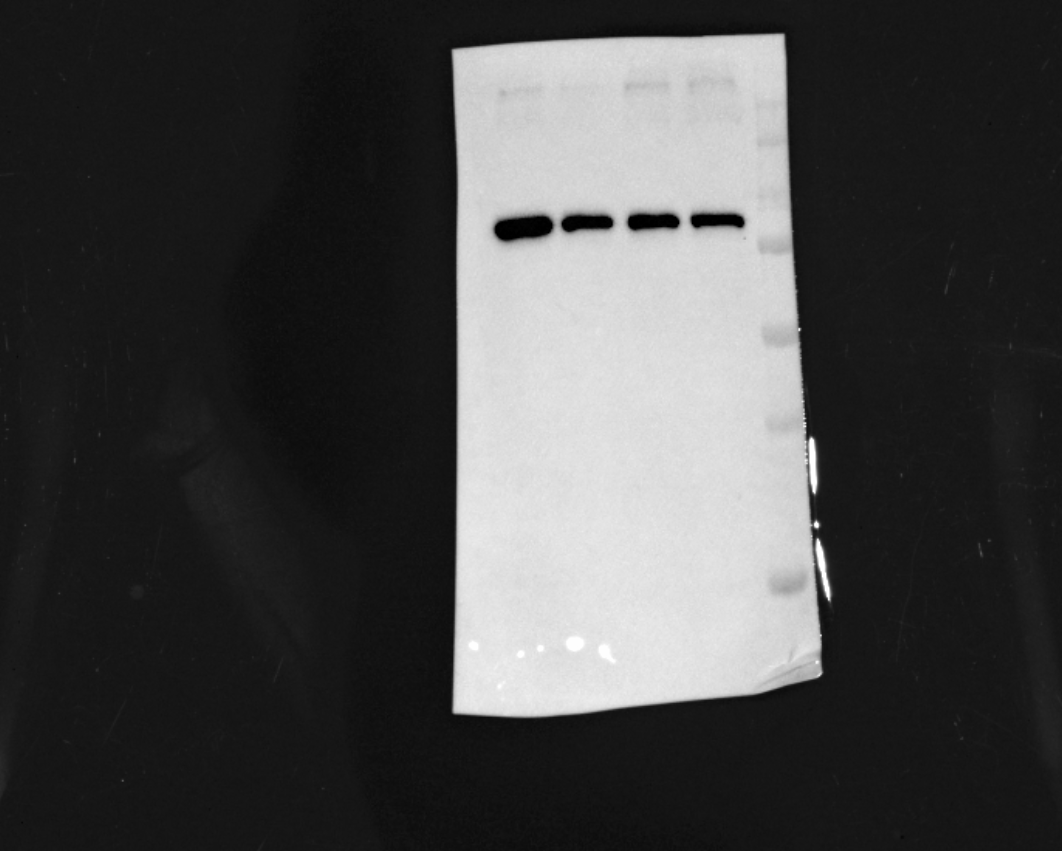

Supplement: Figure 1—figure supplement 4—source data 2. [file elife-97019-fig1-figsupp4-data2.zip › Figure 1-figure supplement 4, source data 2/IP.tif]

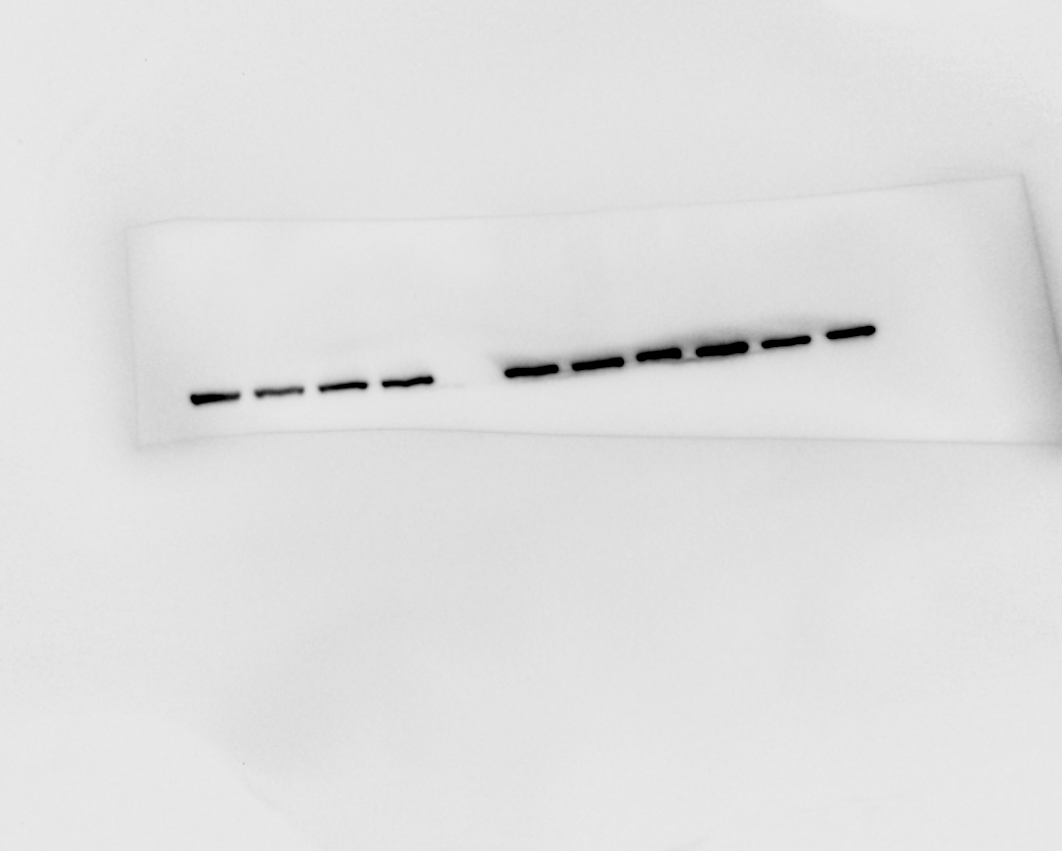

Supplement: Figure 1—figure supplement 4—source data 2. [file elife-97019-fig1-figsupp4-data2.zip › Figure 1-figure supplement 4, source data 2/flag.tif]

IB:ACSS2

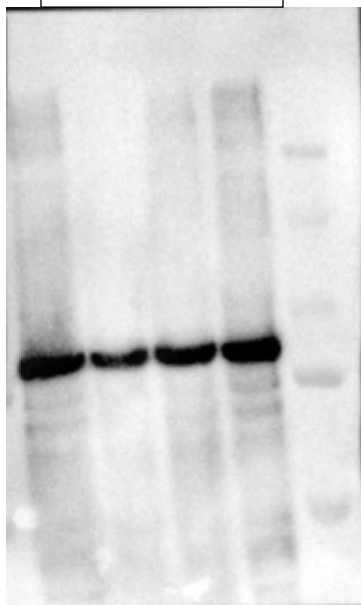

IB:SIRT2

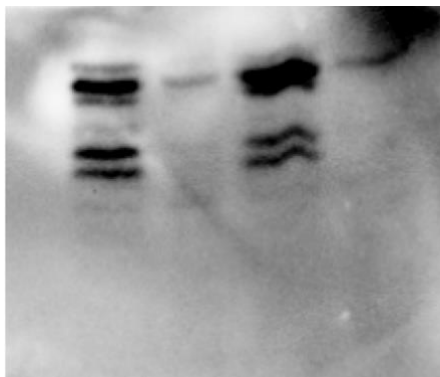

IB:Actin

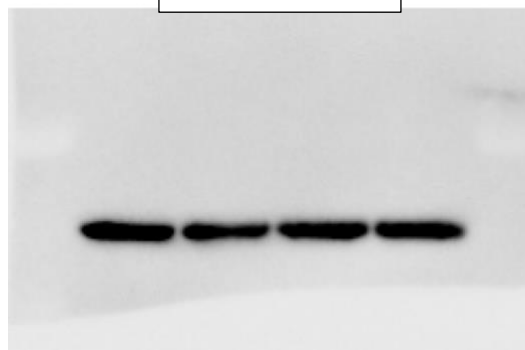

IB:ACSS2

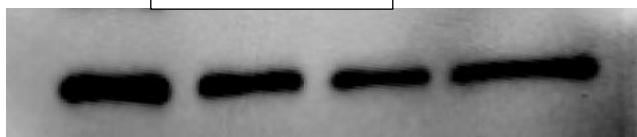

Supplement: Figure 1—figure supplement 5—source data 1. [file elife-97019-fig1-figsupp5-data1.zip › Figure 1-supplement 5.pdf]

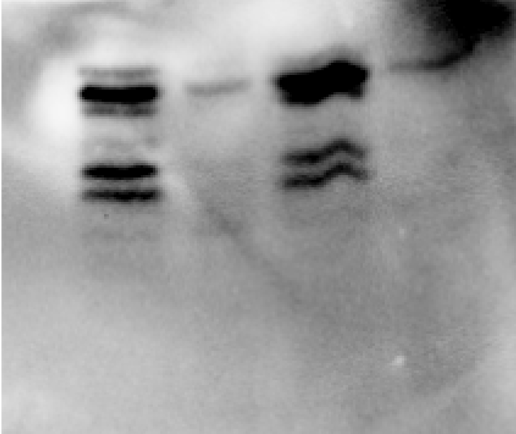

Supplement: Figure 1—figure supplement 5—source data 2. [file elife-97019-fig1-figsupp5-data2.zip › Figure 1-supplement 5-source data 2/19349510-0c1d-4489-9315-c9ff2f631ae1.tiff]

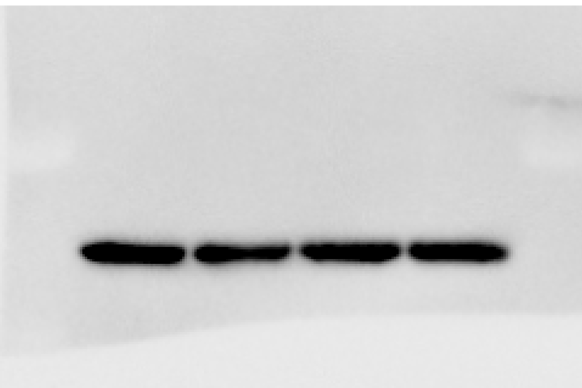

Supplement: Figure 1—figure supplement 5—source data 2. [file elife-97019-fig1-figsupp5-data2.zip › Figure 1-supplement 5-source data 2/866181af-fc52-46d3-9c62-fa5ae6dbad90.tiff]

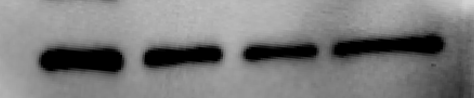

Supplement: Figure 1—figure supplement 5—source data 2. [file elife-97019-fig1-figsupp5-data2.zip › Figure 1-supplement 5-source data 2/a0ffadf7-f9da-494f-83df-d405e82a7862 (1).tiff]

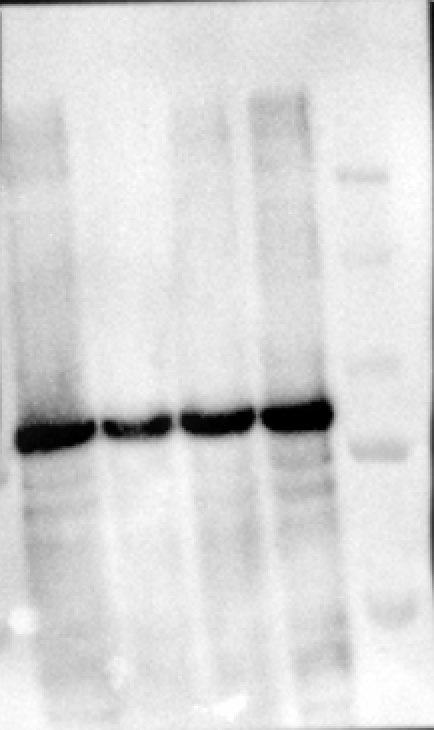

Supplement: Figure 1—figure supplement 5—source data 2. [file elife-97019-fig1-figsupp5-data2.zip › Figure 1-supplement 5-source data 2/bf3c4287-febf-4b49-a5ed-fbbdaab1b10e.tiff]

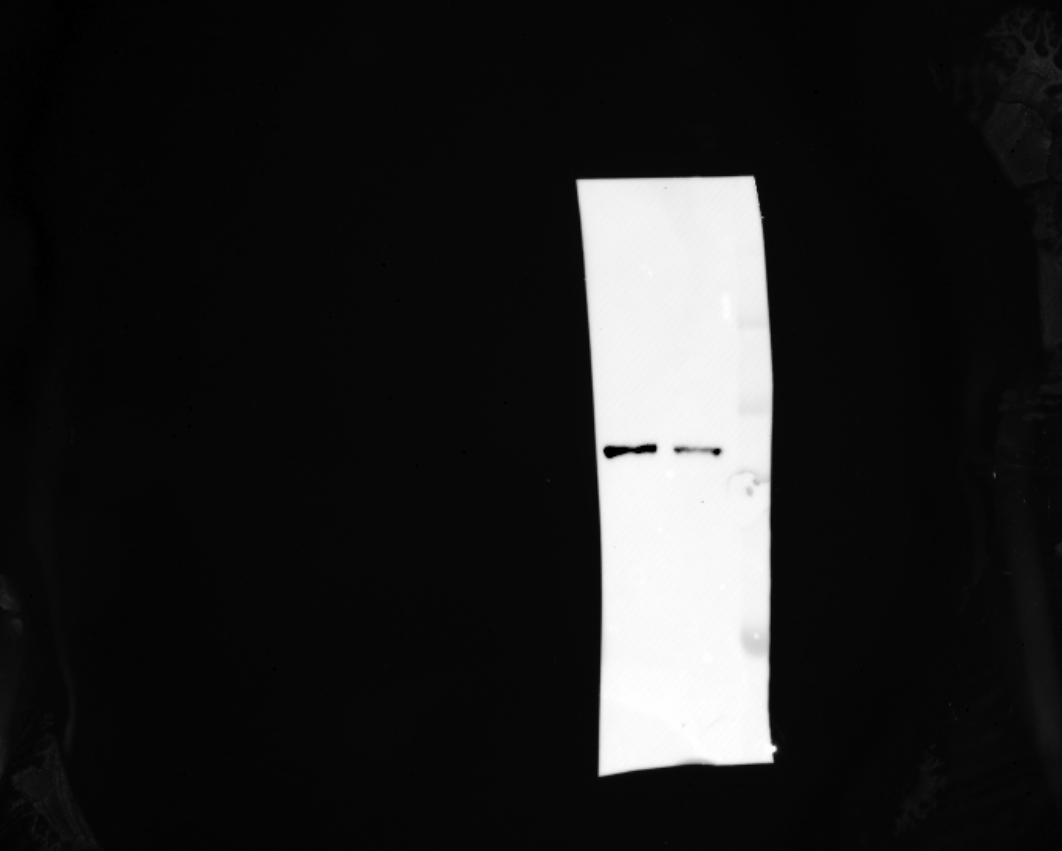

Supplement: Figure 2—source data 2. [file elife-97019-fig2-data2.zip › Figure 2-source data 2/2G/input.tif]

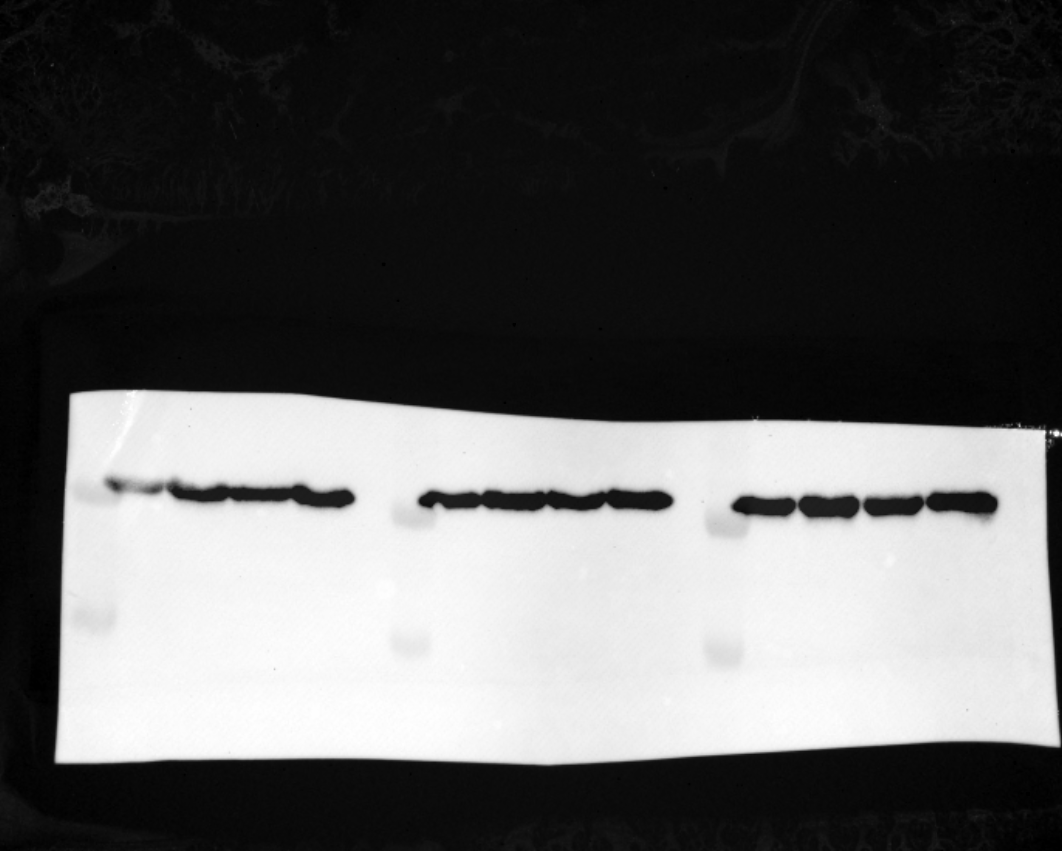

Supplement: Figure 2—source data 2. [file elife-97019-fig2-data2.zip › Figure 2-source data 2/2A/Actin.tif]

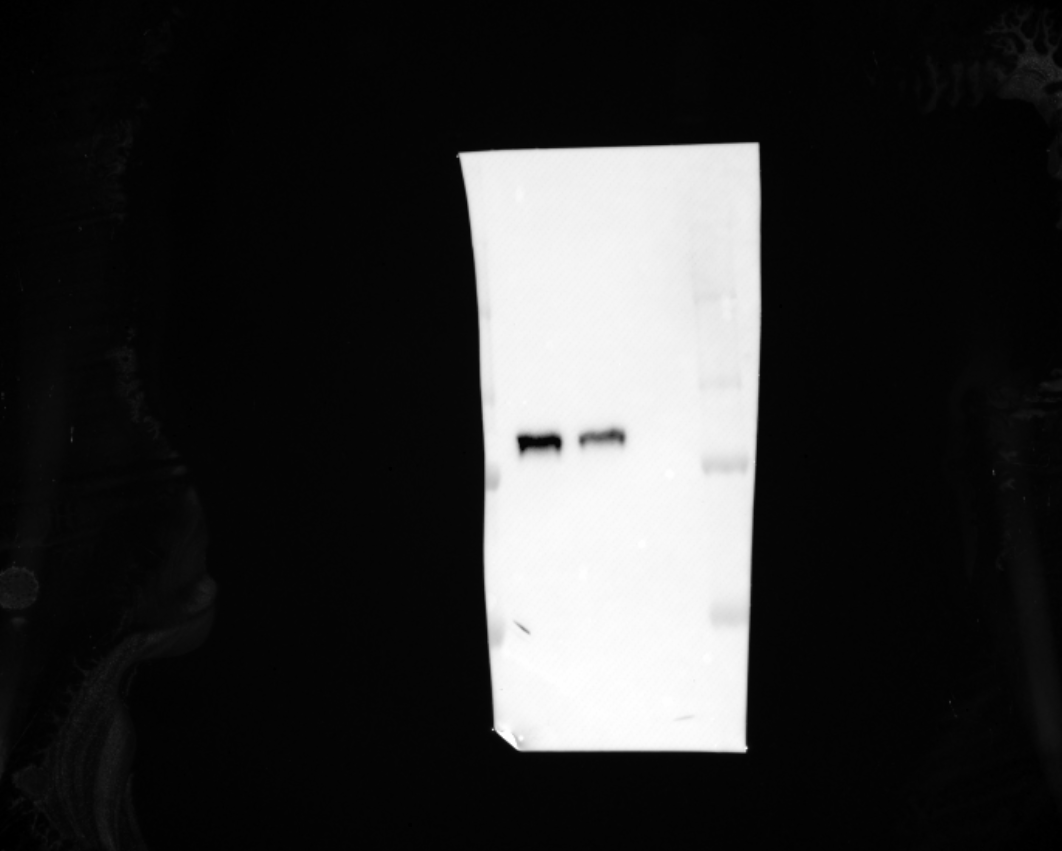

Supplement: Figure 2—source data 2. [file elife-97019-fig2-data2.zip › Figure 2-source data 2/2G/IP.tif]

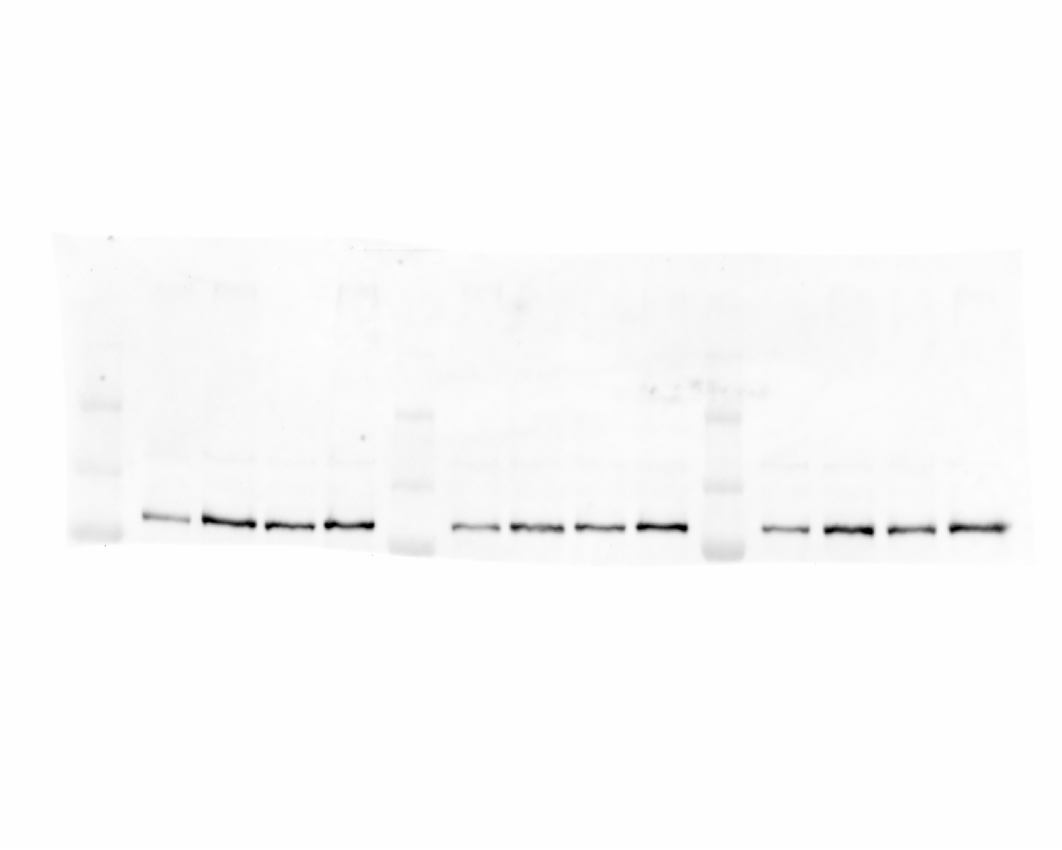

Supplement: Figure 2—source data 2. [file elife-97019-fig2-data2.zip › Figure 2-source data 2/2A/ACSS2.tif]

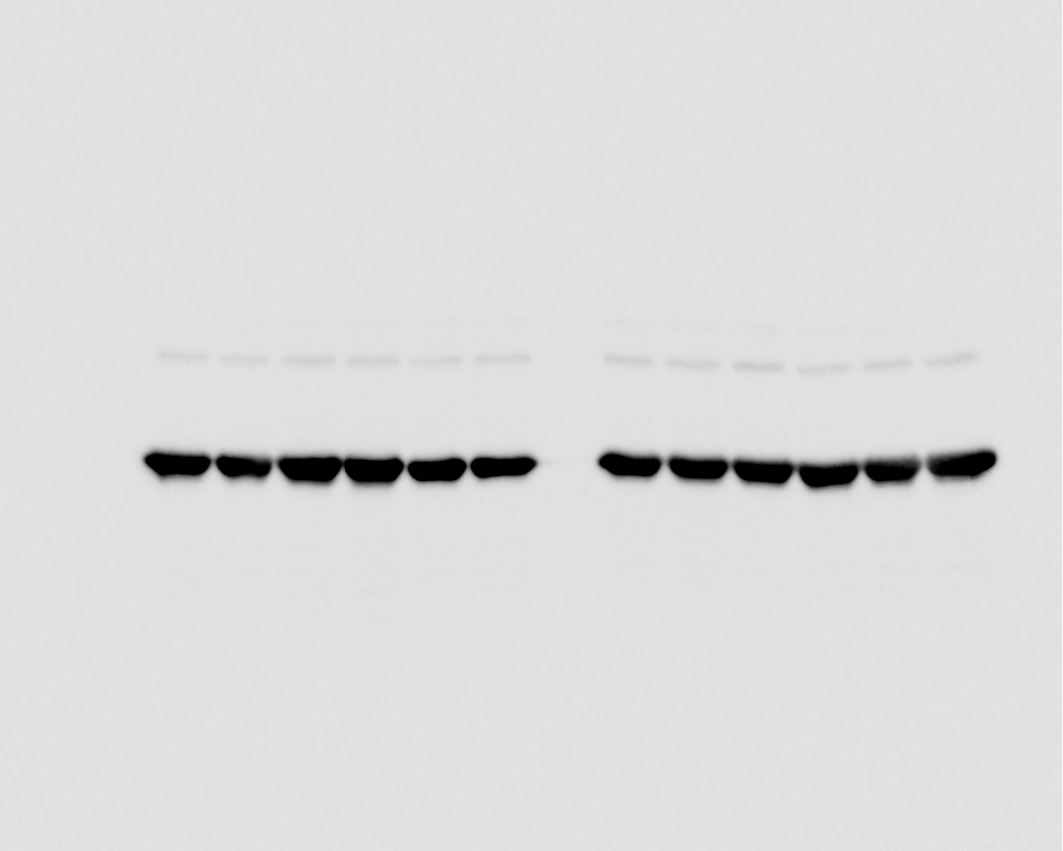

Supplement: Figure 2—source data 2. [file elife-97019-fig2-data2.zip › Figure 2-source data 2/2C/Acrin.tif]

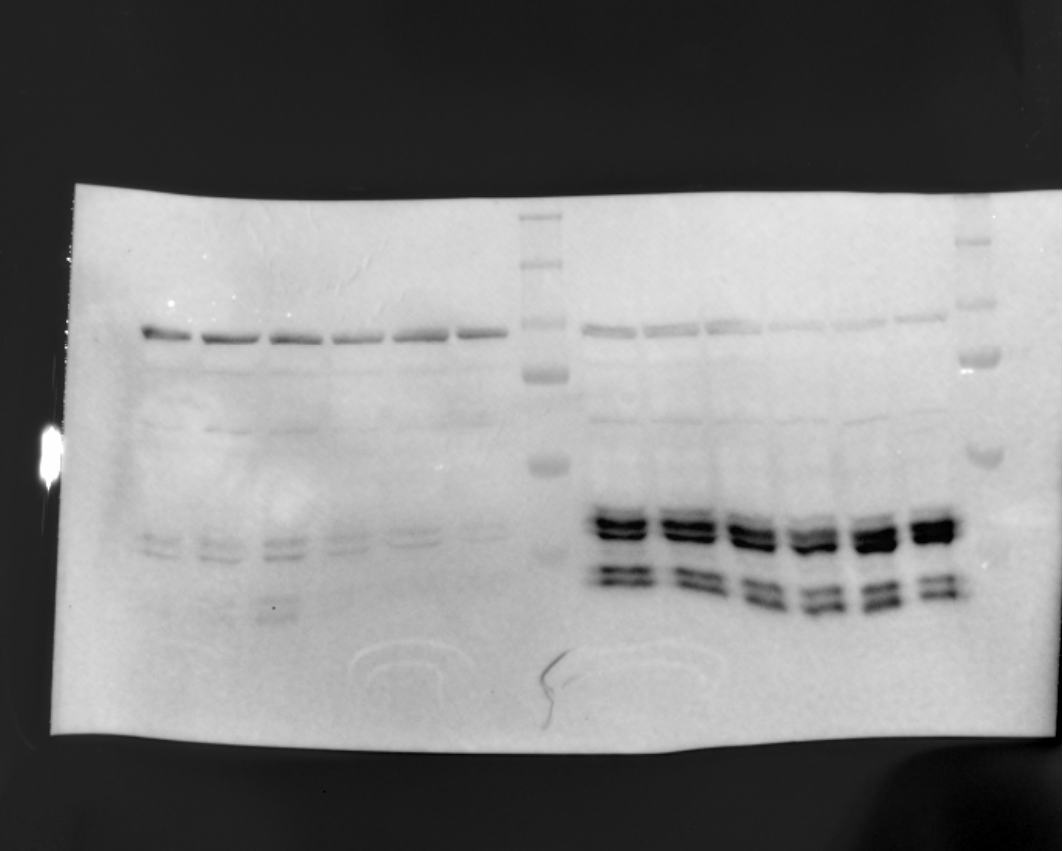

Supplement: Figure 2—source data 2. [file elife-97019-fig2-data2.zip › Figure 2-source data 2/2C/SIRT2.tif]

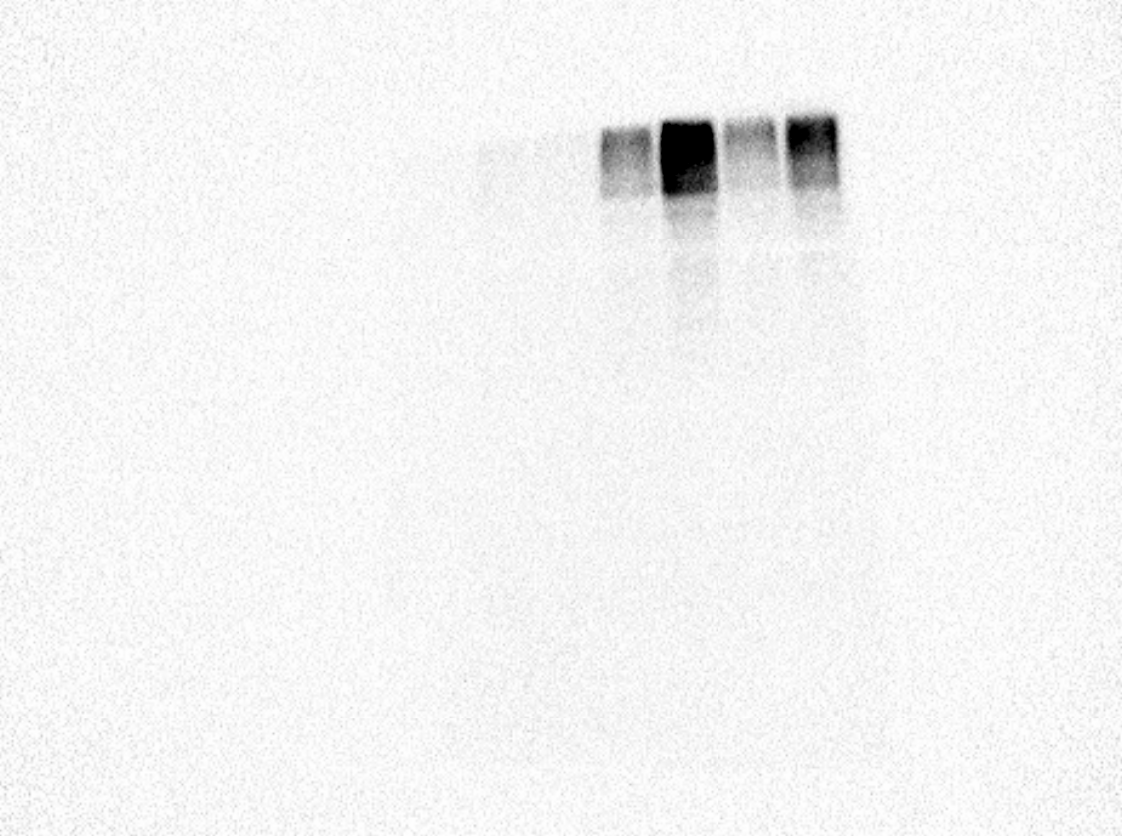

Supplement: Figure 2—source data 2. [file elife-97019-fig2-data2.zip › Figure 2-source data 2/2E/Ub.tif]

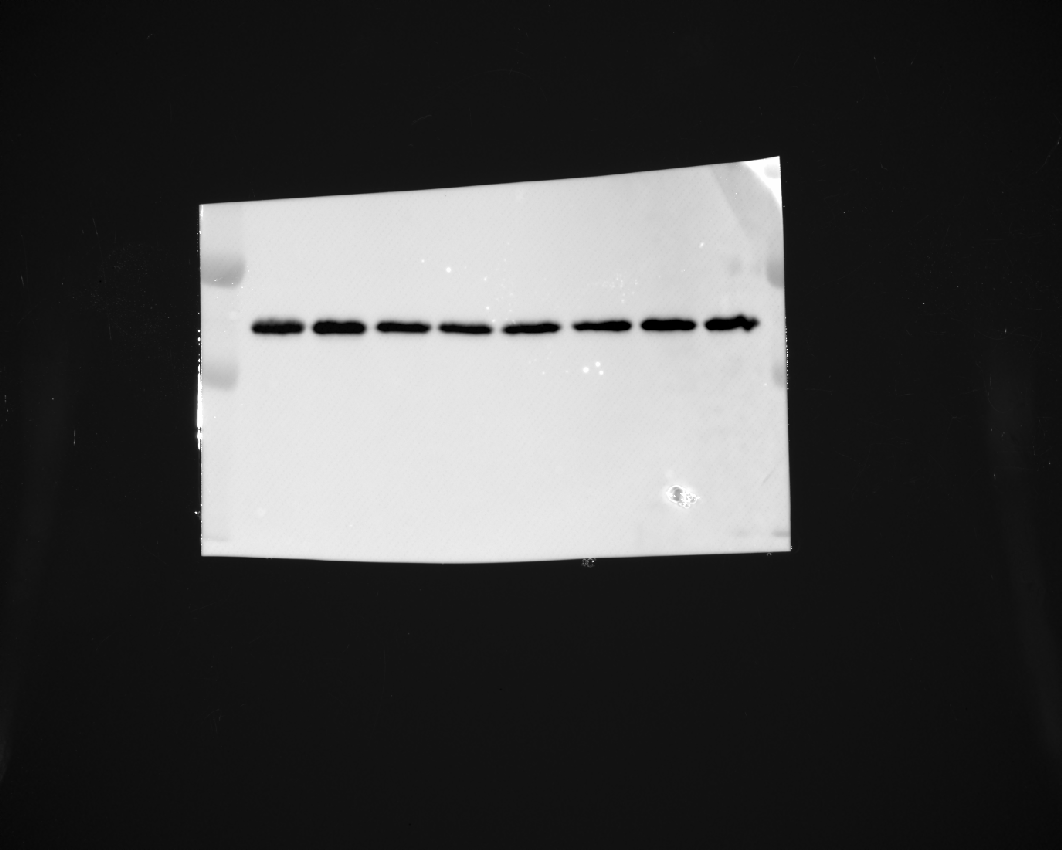

Supplement: Figure 2—source data 2. [file elife-97019-fig2-data2.zip › Figure 2-source data 2/2B/Actin.tif]

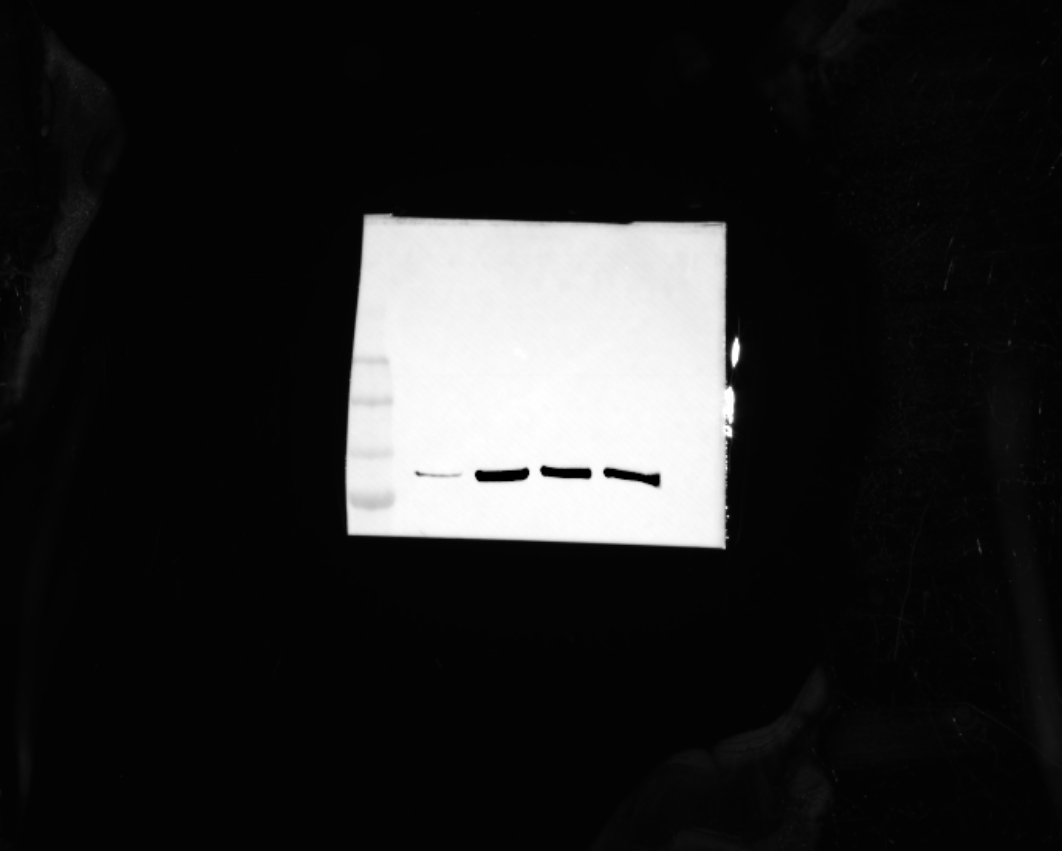

Supplement: Figure 2—source data 2. [file elife-97019-fig2-data2.zip › Figure 2-source data 2/2F/IP-Flag.tif]

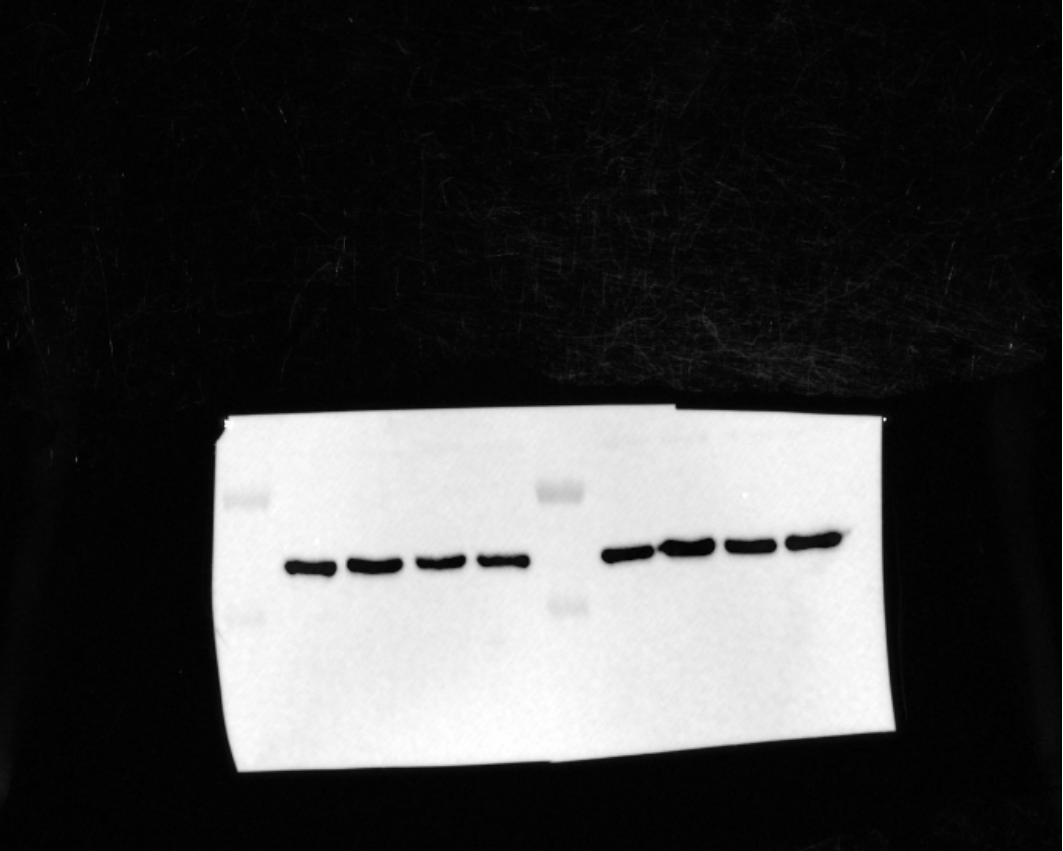

Supplement: Figure 2—source data 2. [file elife-97019-fig2-data2.zip › Figure 2-source data 2/2H/Actin.tif]

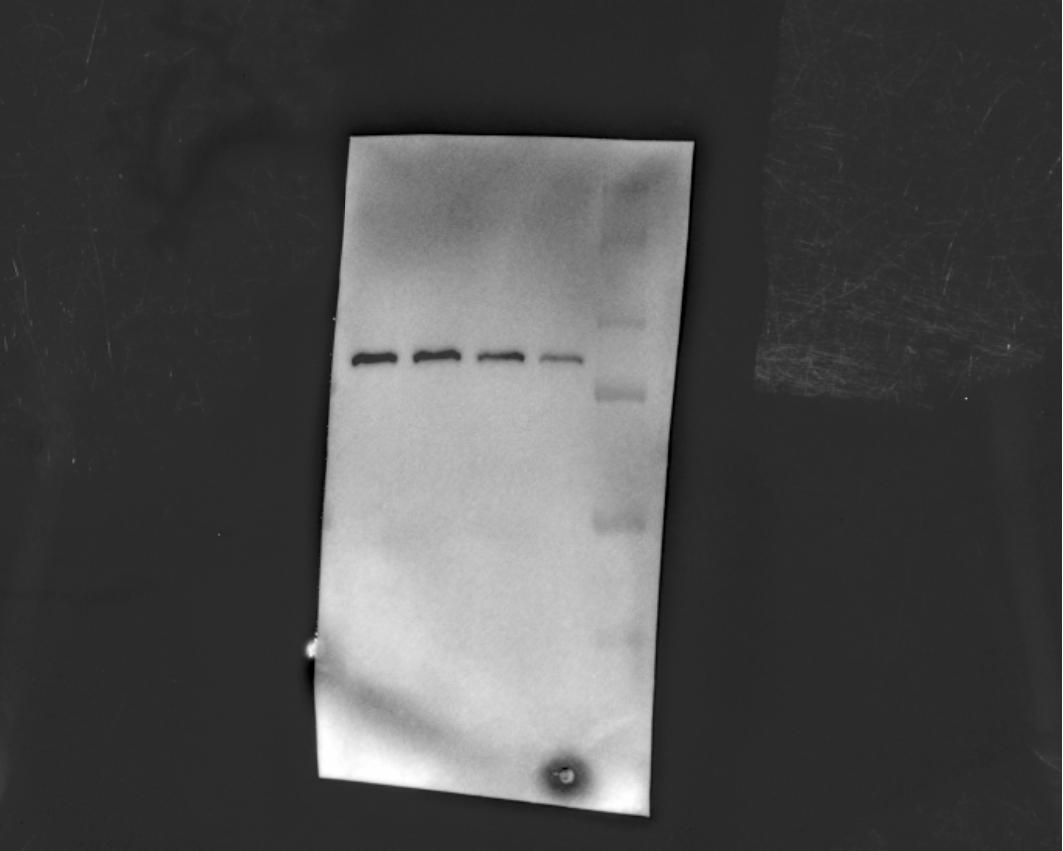

Supplement: Figure 2—source data 2. [file elife-97019-fig2-data2.zip › Figure 2-source data 2/2H/IP.tif]

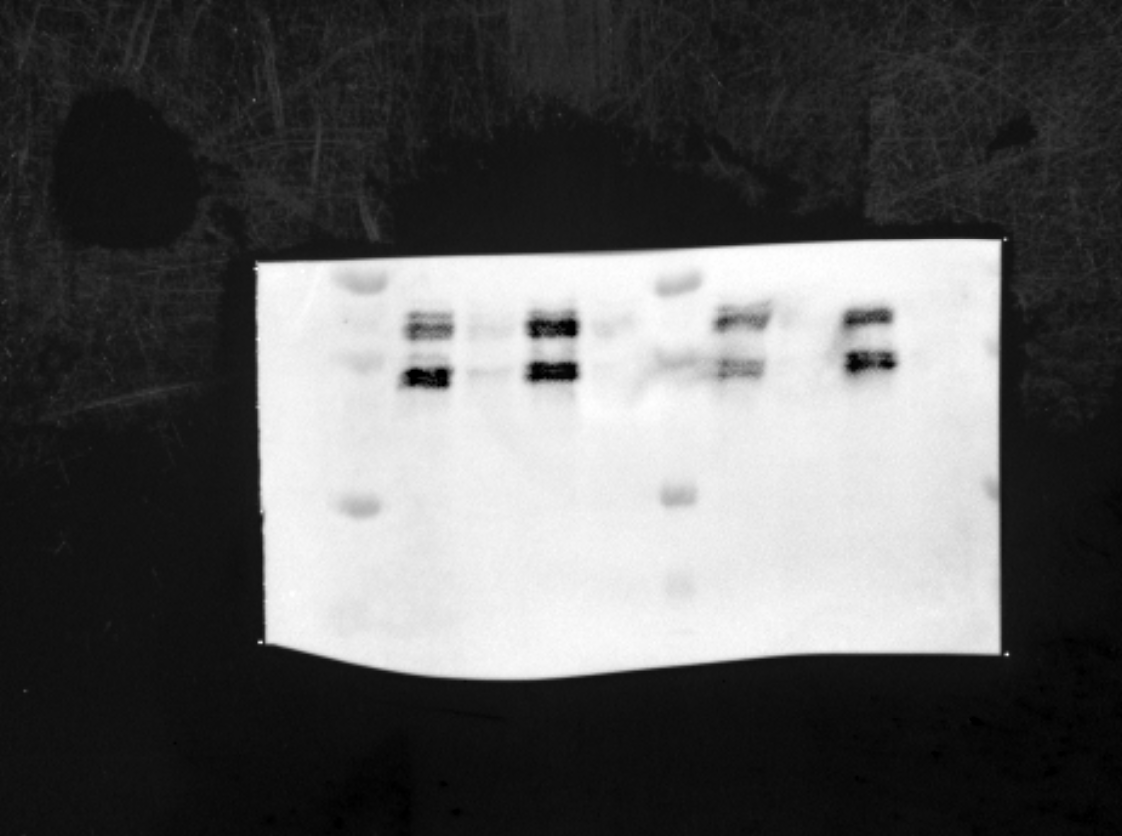

Supplement: Figure 2—source data 2. [file elife-97019-fig2-data2.zip › Figure 2-source data 2/2B/SIRT2.tif]

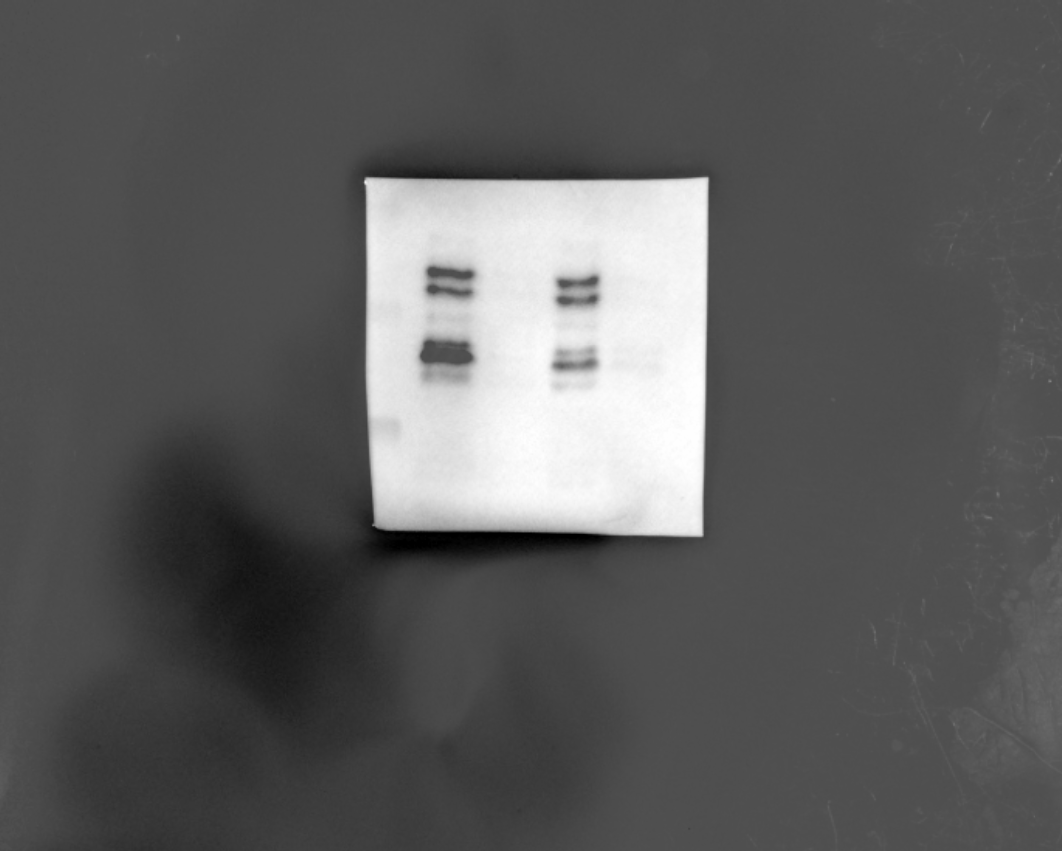

Supplement: Figure 2—source data 2. [file elife-97019-fig2-data2.zip › Figure 2-source data 2/2H/SIRT2.tif]

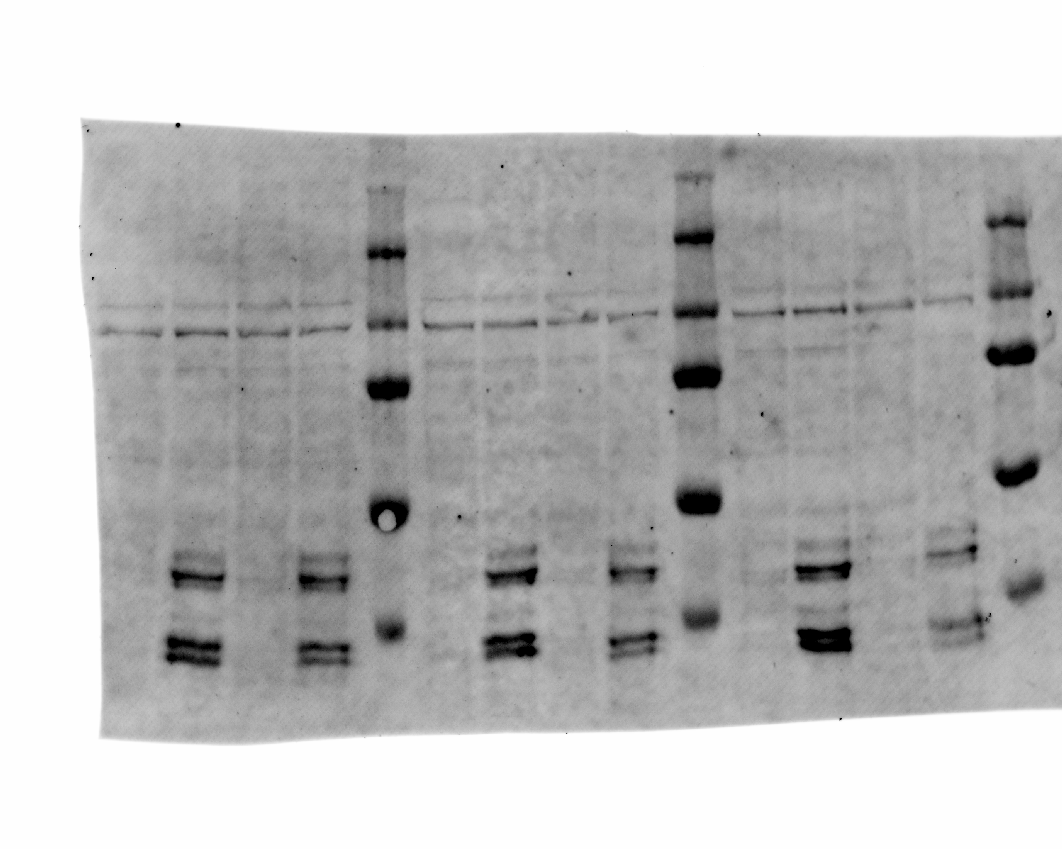

Supplement: Figure 2—source data 2. [file elife-97019-fig2-data2.zip › Figure 2-source data 2/2A/SIRT2.tif]

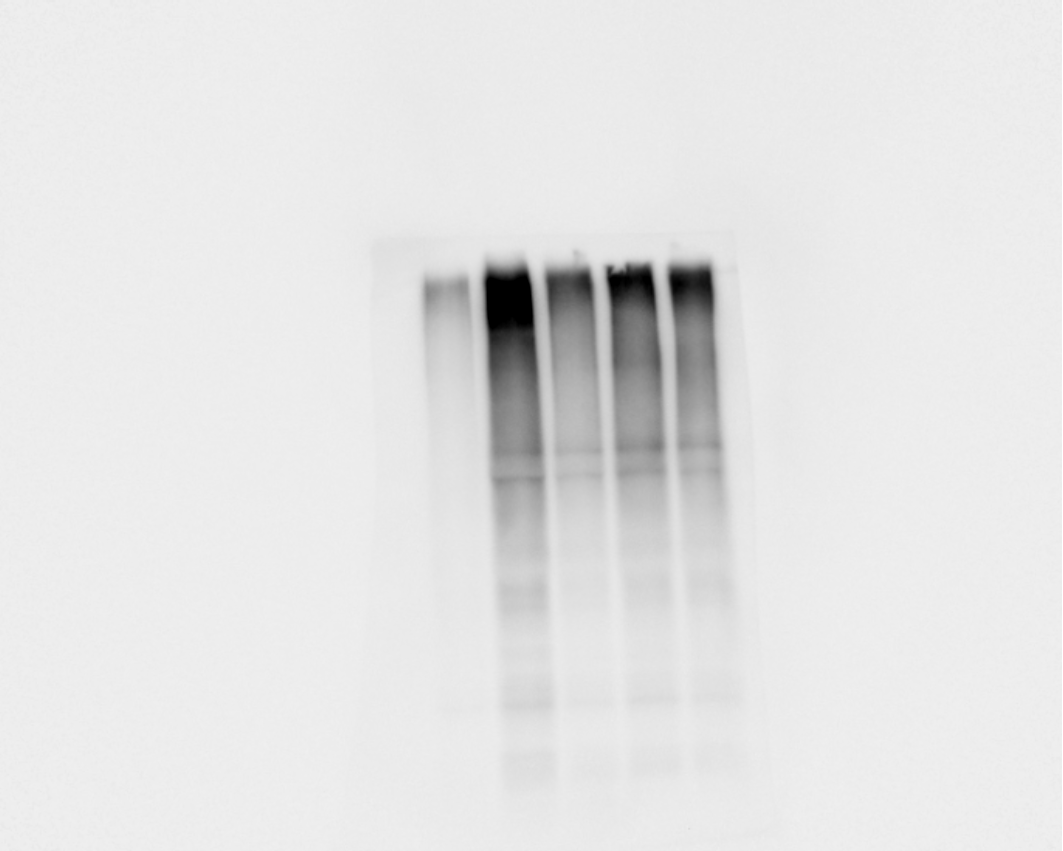

Supplement: Figure 2—source data 2. [file elife-97019-fig2-data2.zip › Figure 2-source data 2/2I/K48 IP.tif]

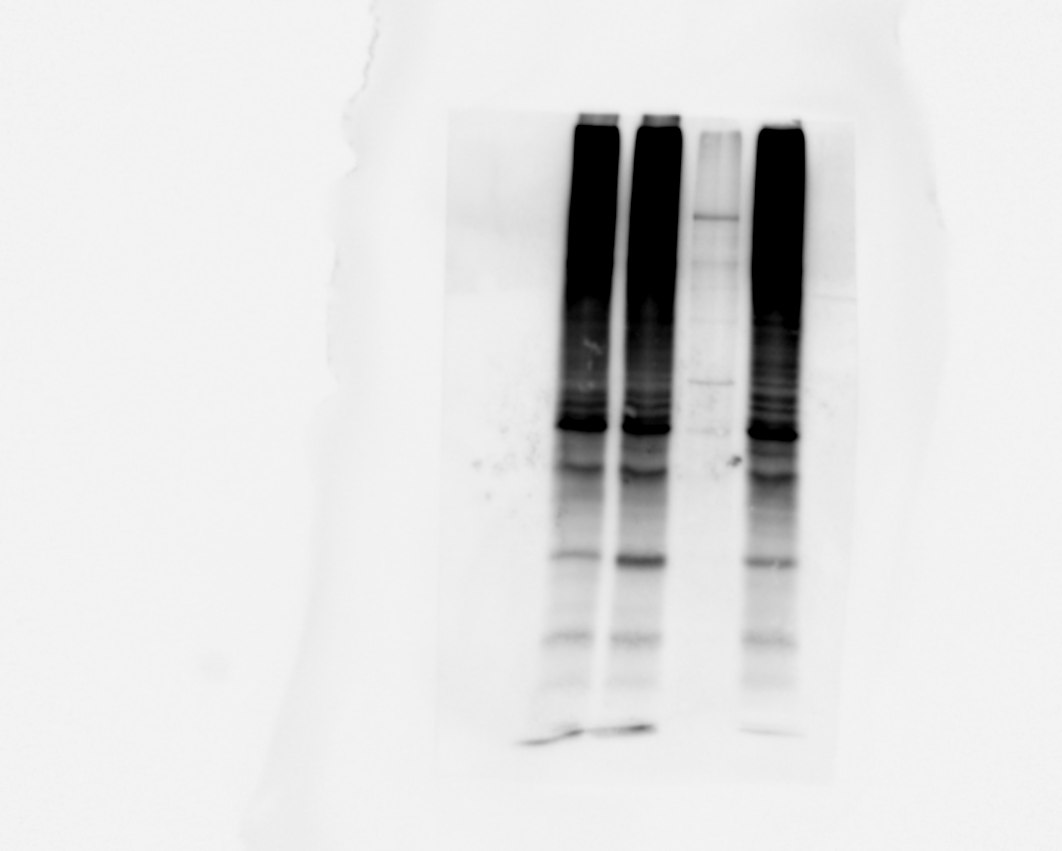

Supplement: Figure 2—source data 2. [file elife-97019-fig2-data2.zip › Figure 2-source data 2/2F/Input IB-HA.tif]

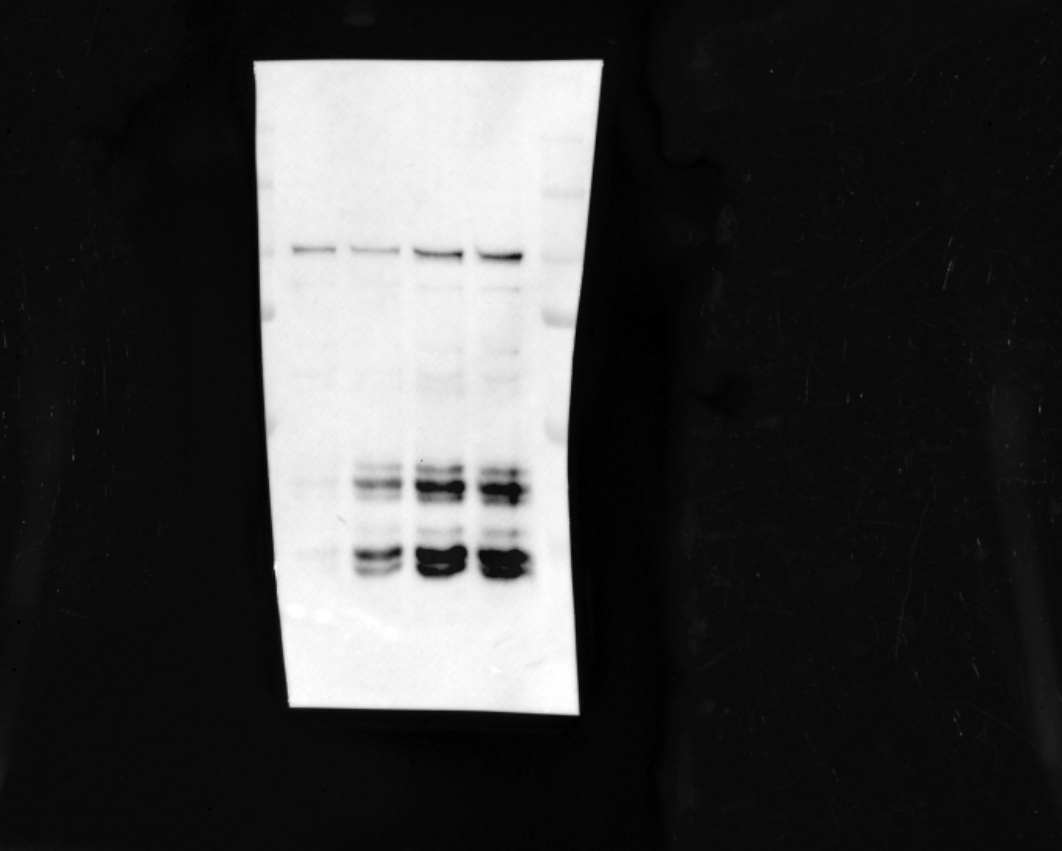

Supplement: Figure 2—source data 2. [file elife-97019-fig2-data2.zip › Figure 2-source data 2/2F/SIRT2.tif]

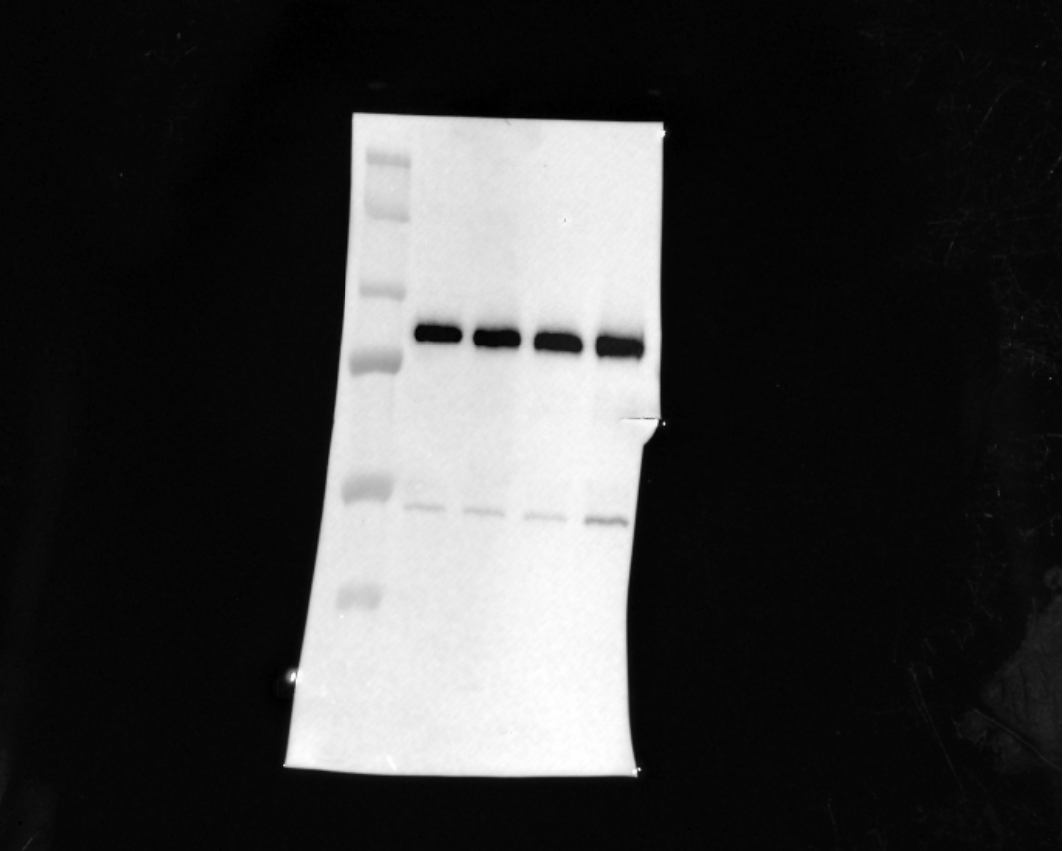

Supplement: Figure 2—source data 2. [file elife-97019-fig2-data2.zip › Figure 2-source data 2/2I/Flag.tif]

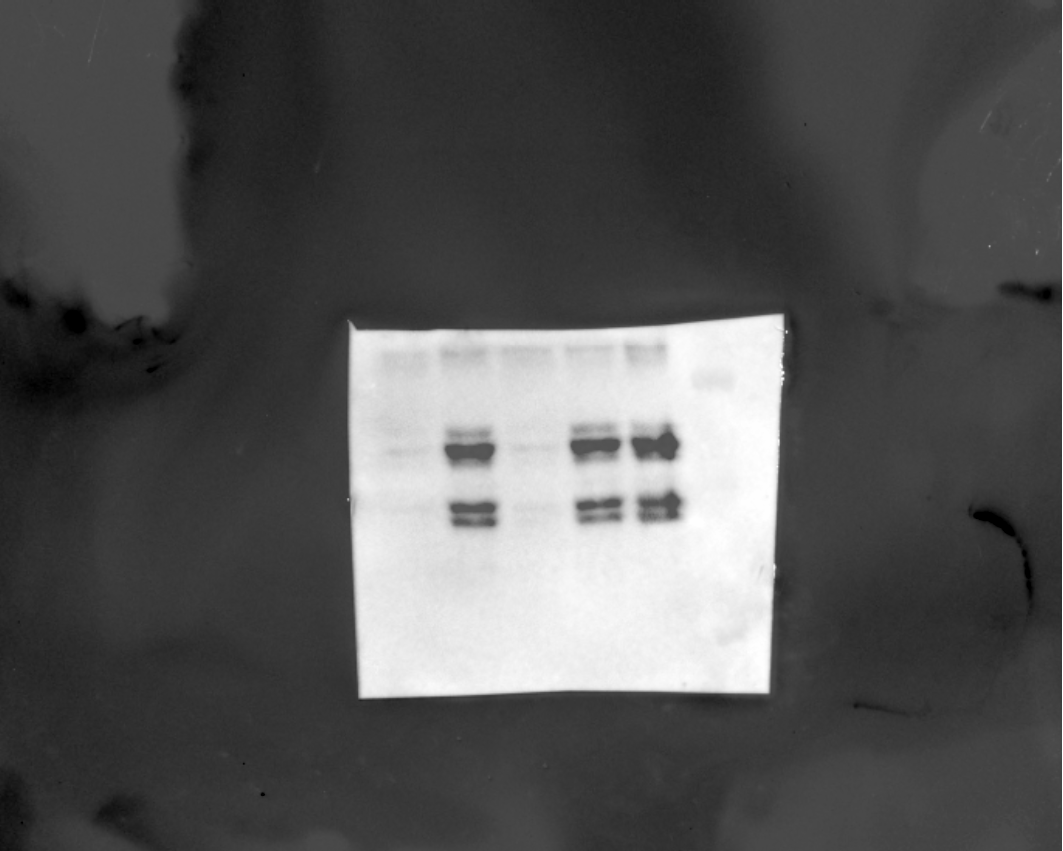

Supplement: Figure 2—source data 2. [file elife-97019-fig2-data2.zip › Figure 2-source data 2/2I/SIRT2.tif]

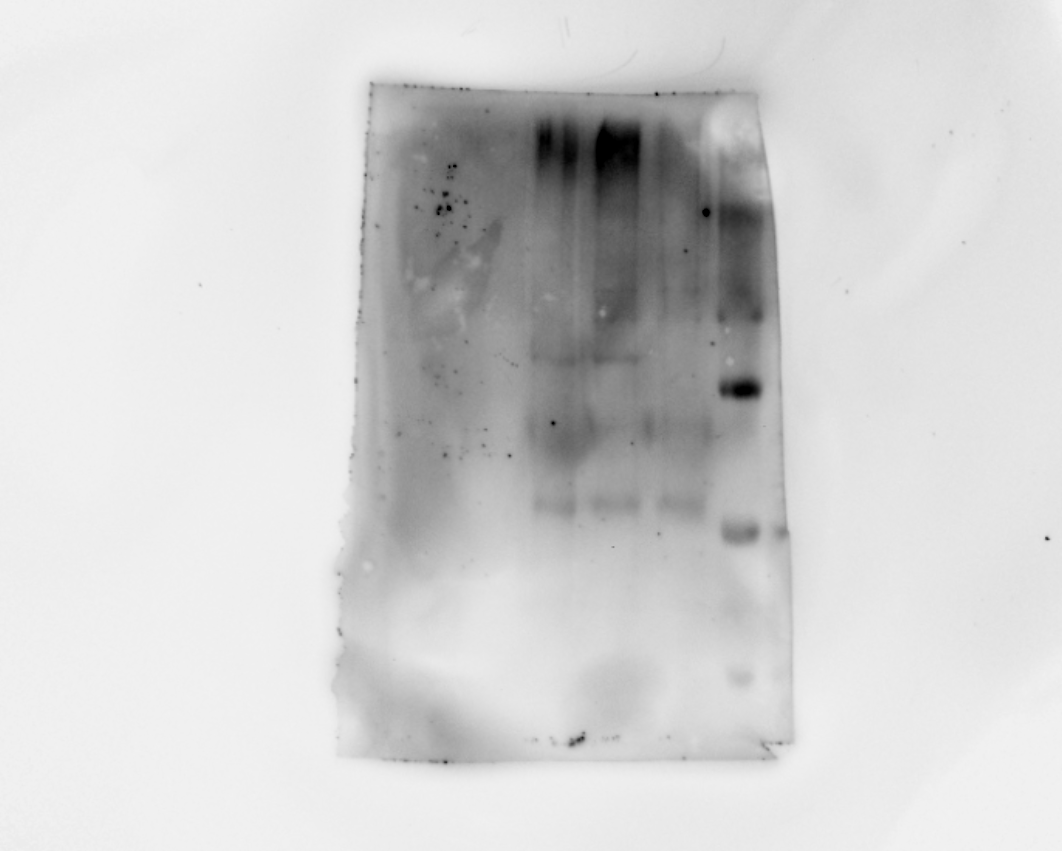

Supplement: Figure 2—source data 2. [file elife-97019-fig2-data2.zip › Figure 2-source data 2/2G/K48.tif]

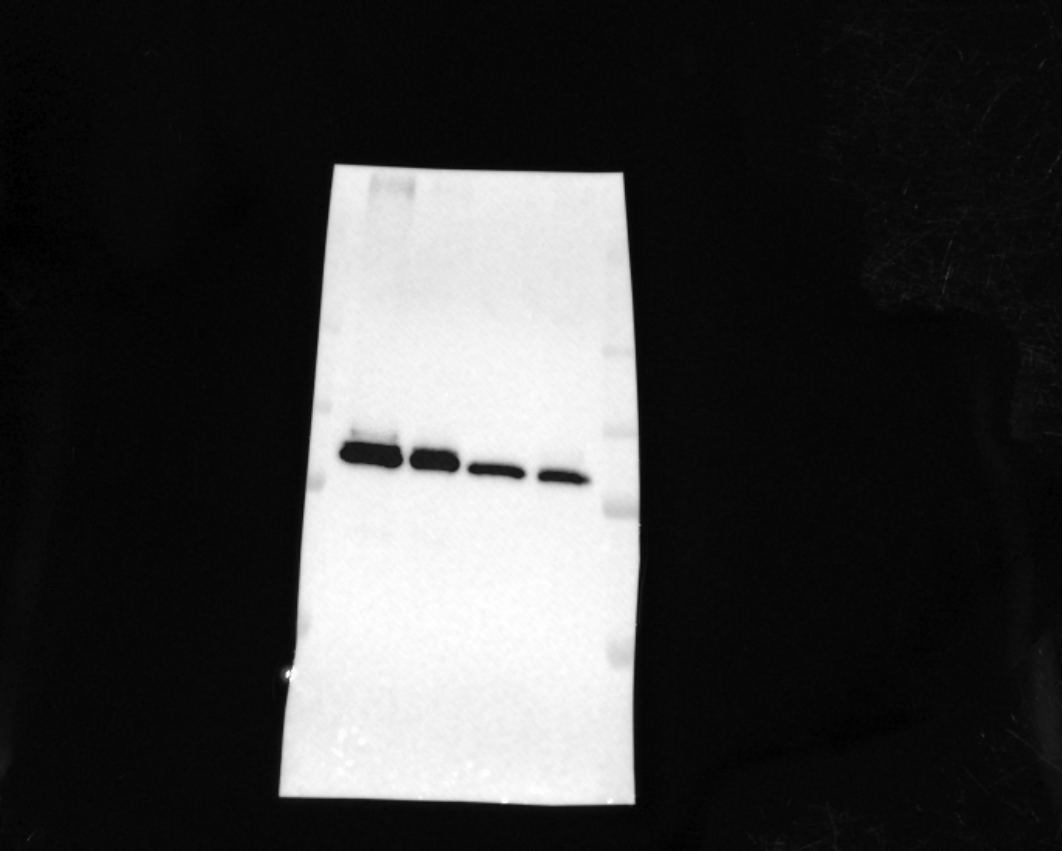

Supplement: Figure 2—source data 2. [file elife-97019-fig2-data2.zip › Figure 2-source data 2/2H/Flag.tif]

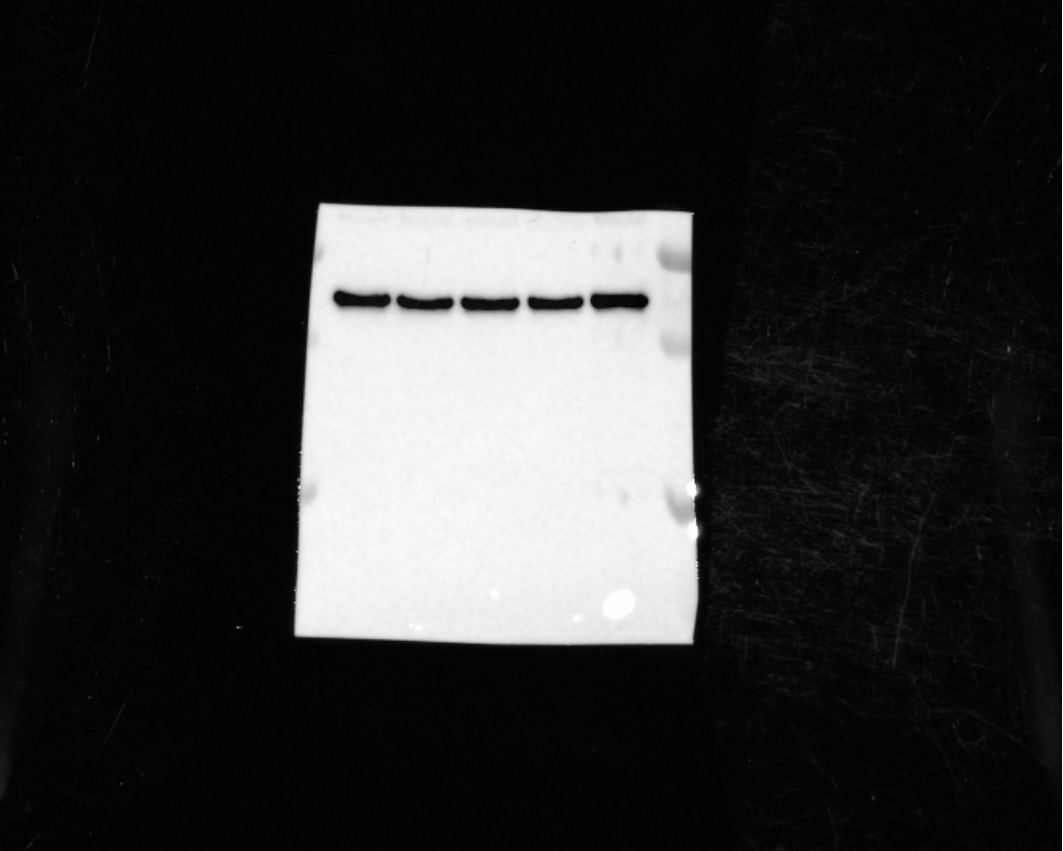

Supplement: Figure 2—source data 2. [file elife-97019-fig2-data2.zip › Figure 2-source data 2/2I/Actin.tif]

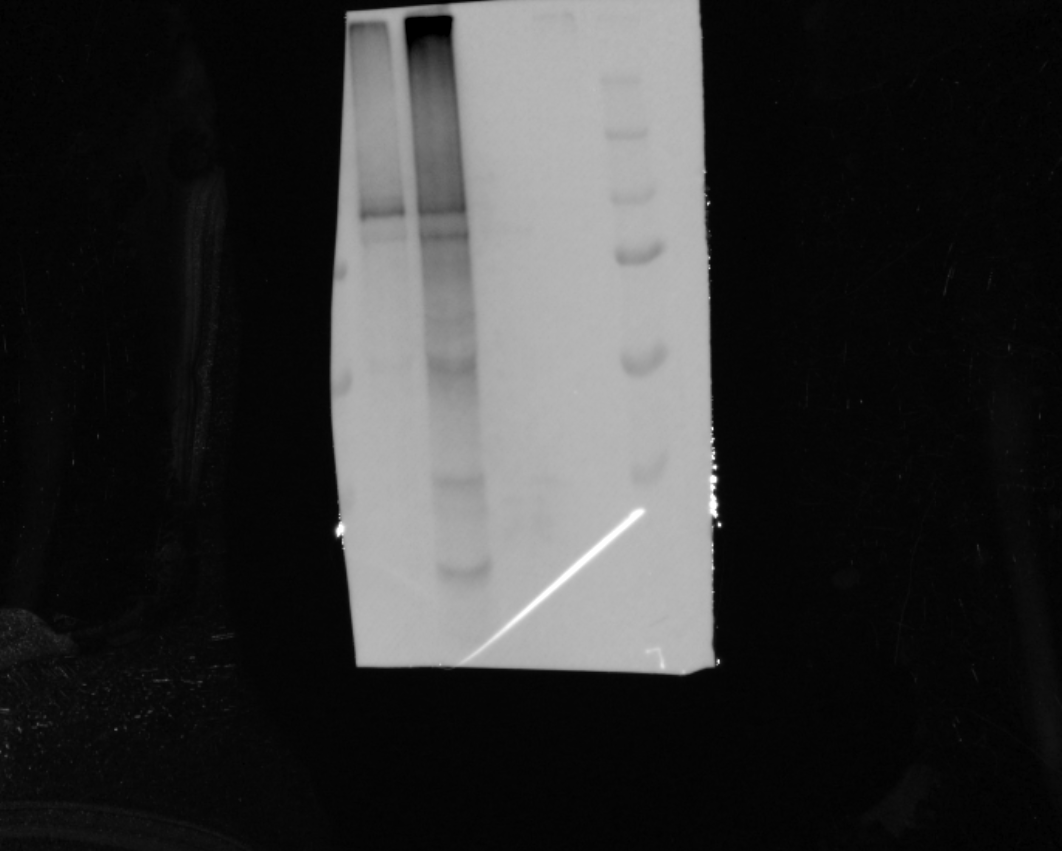

Supplement: Figure 2—source data 2. [file elife-97019-fig2-data2.zip › Figure 2-source data 2/2F/IB-HA.tif]

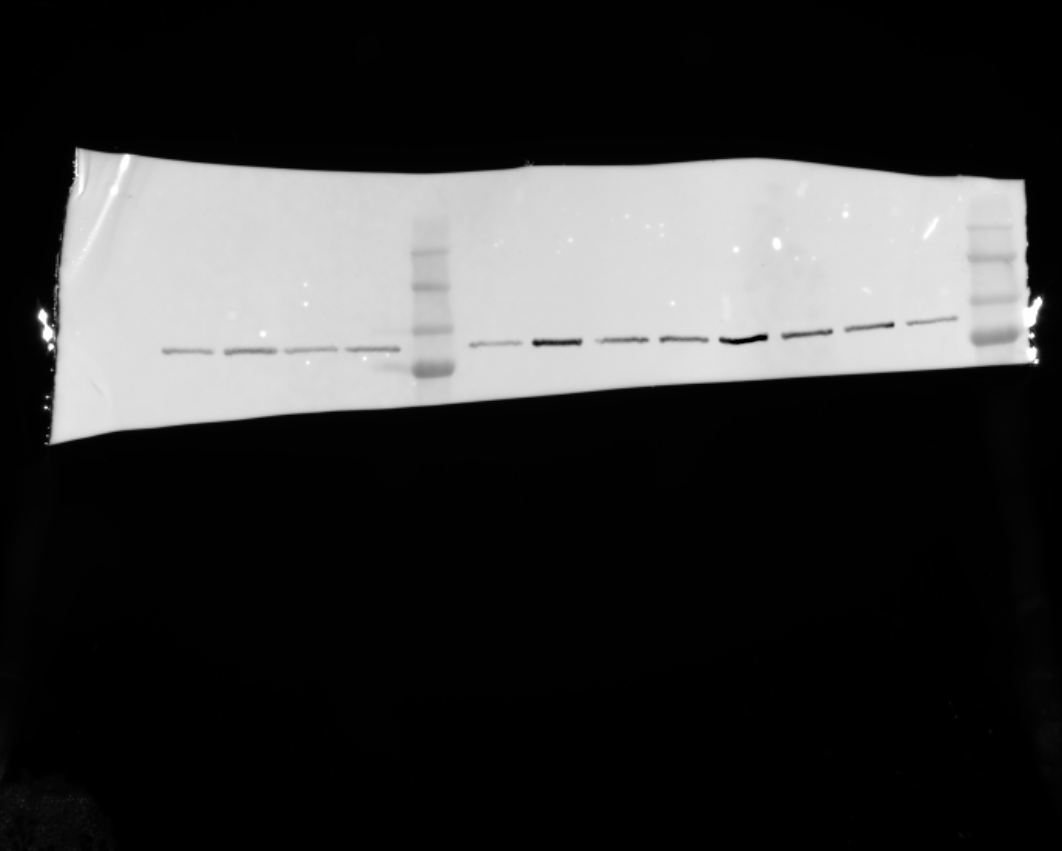

Supplement: Figure 2—source data 2. [file elife-97019-fig2-data2.zip › Figure 2-source data 2/2B/ACSS2.tif]

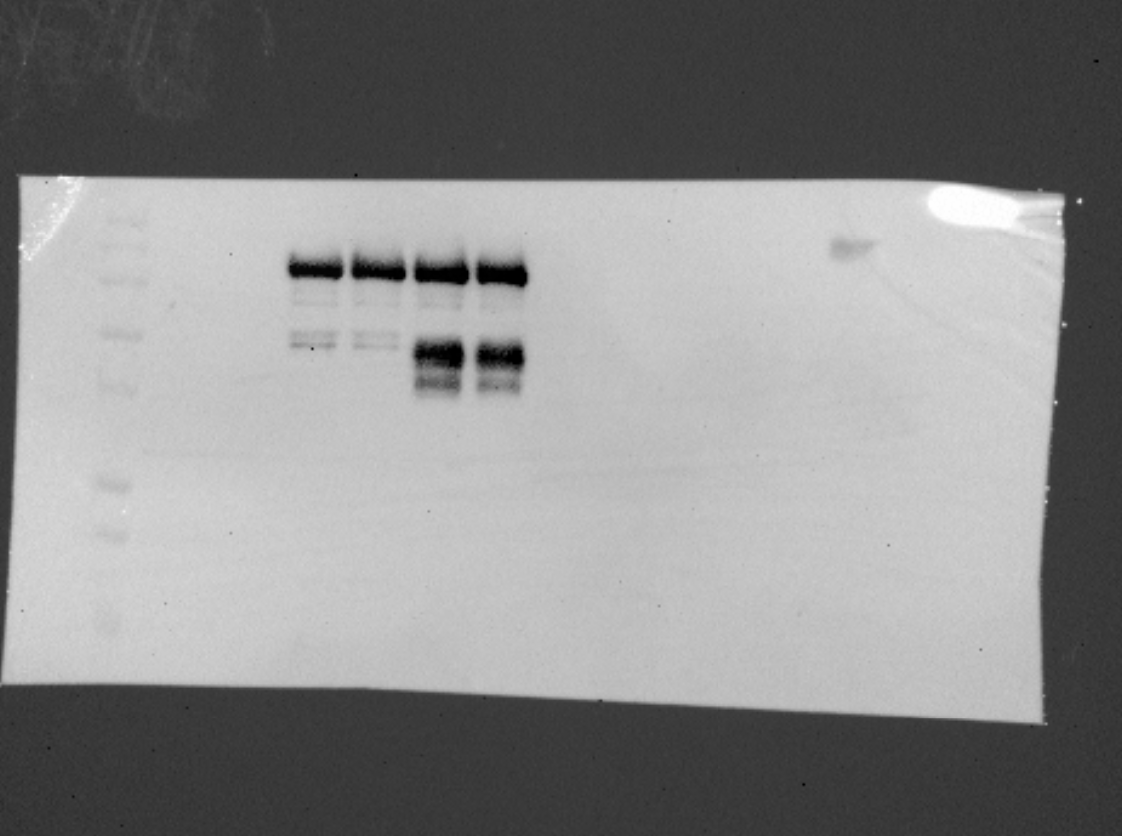

Supplement: Figure 2—source data 2. [file elife-97019-fig2-data2.zip › Figure 2-source data 2/2E/Flag.tif]

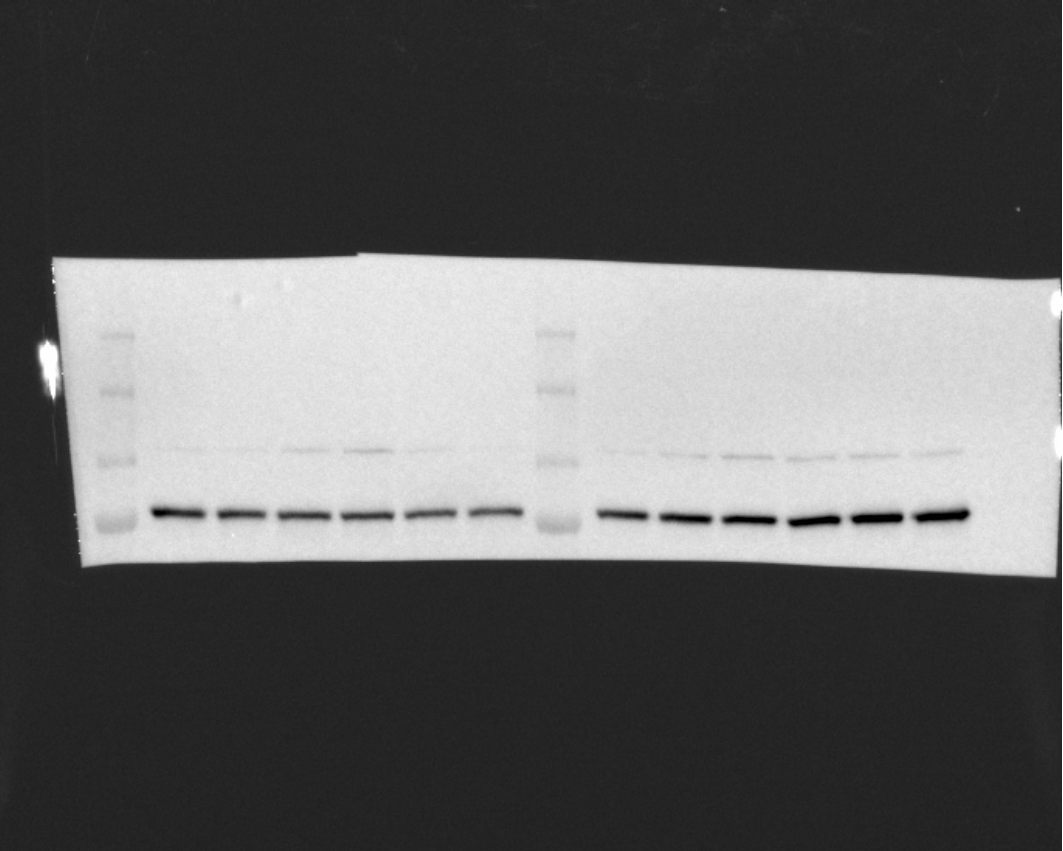

Supplement: Figure 2—source data 2. [file elife-97019-fig2-data2.zip › Figure 2-source data 2/2C/ACSS2.tif]

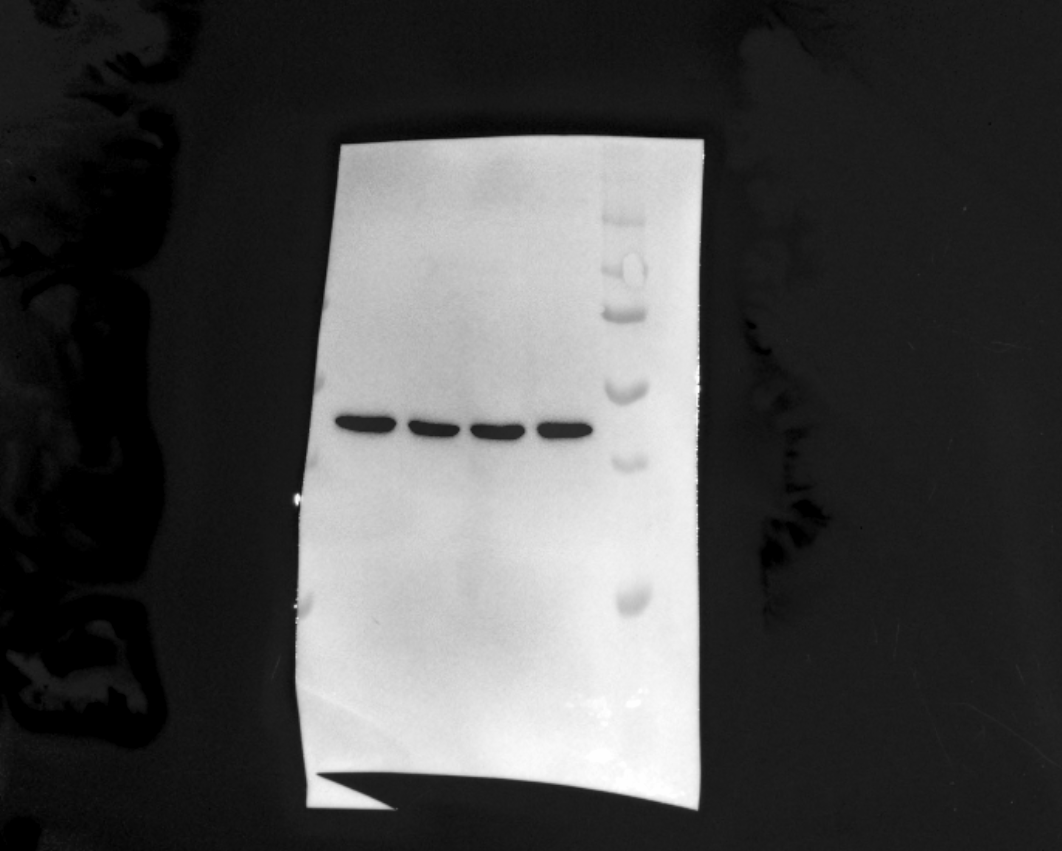

Supplement: Figure 2—source data 2. [file elife-97019-fig2-data2.zip › Figure 2-source data 2/2F/Actin.tif]

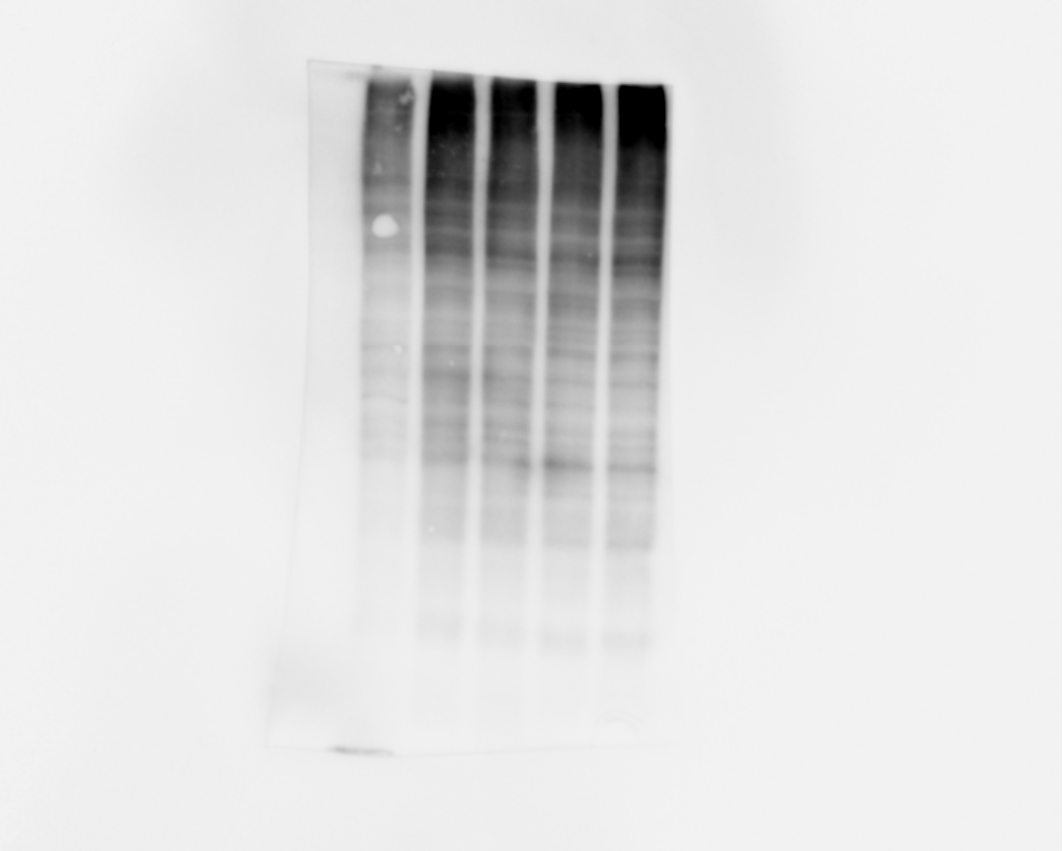

Supplement: Figure 2—source data 2. [file elife-97019-fig2-data2.zip › Figure 2-source data 2/2I/K48 input.tif]

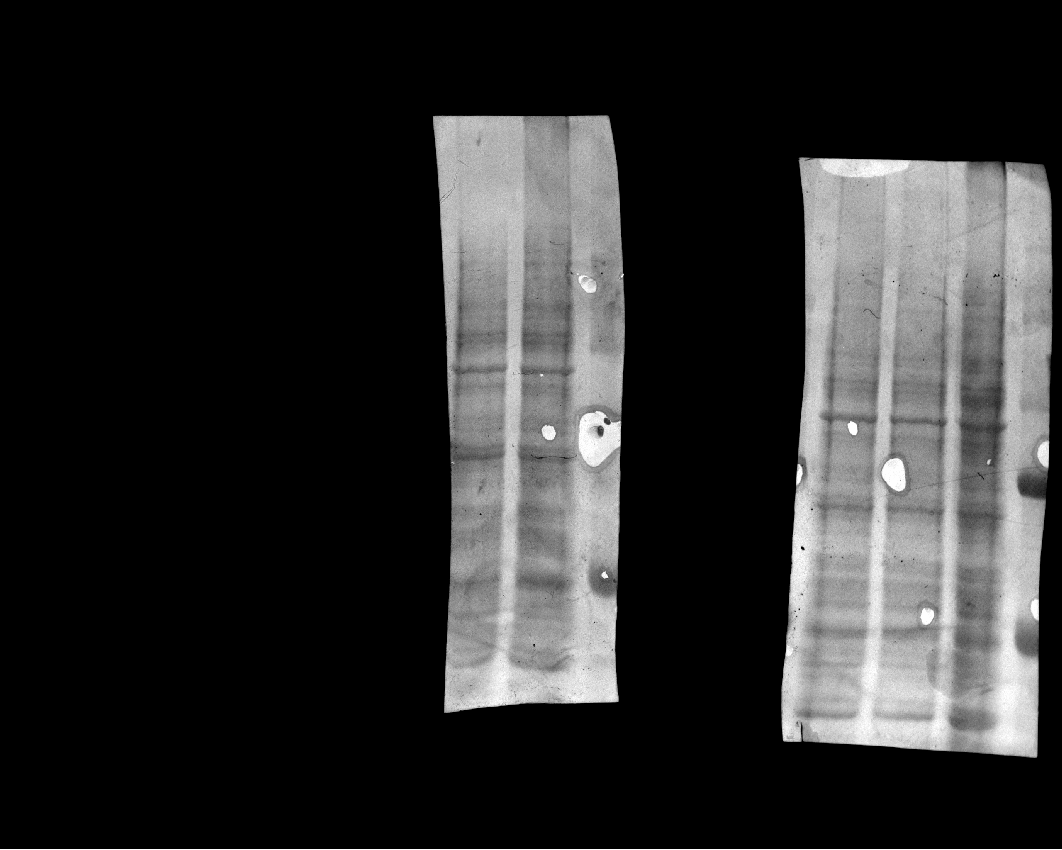

Supplement: Figure 2—source data 2. [file elife-97019-fig2-data2.zip › Figure 2-source data 2/2G/CBB.tif]

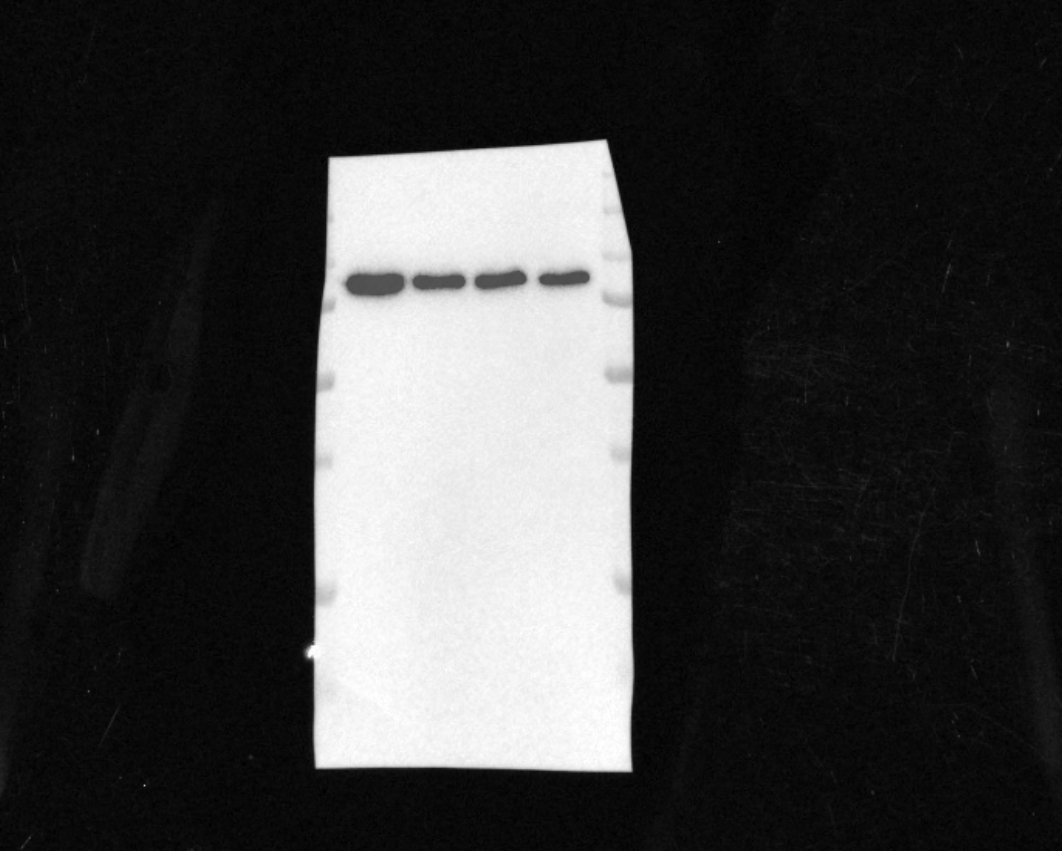

Supplement: Figure 2—figure supplement 1—source data 2. [file elife-97019-fig2-figsupp1-data2.zip › Figure 2-figure supplement 1, source data 2/ACSS12.tif]

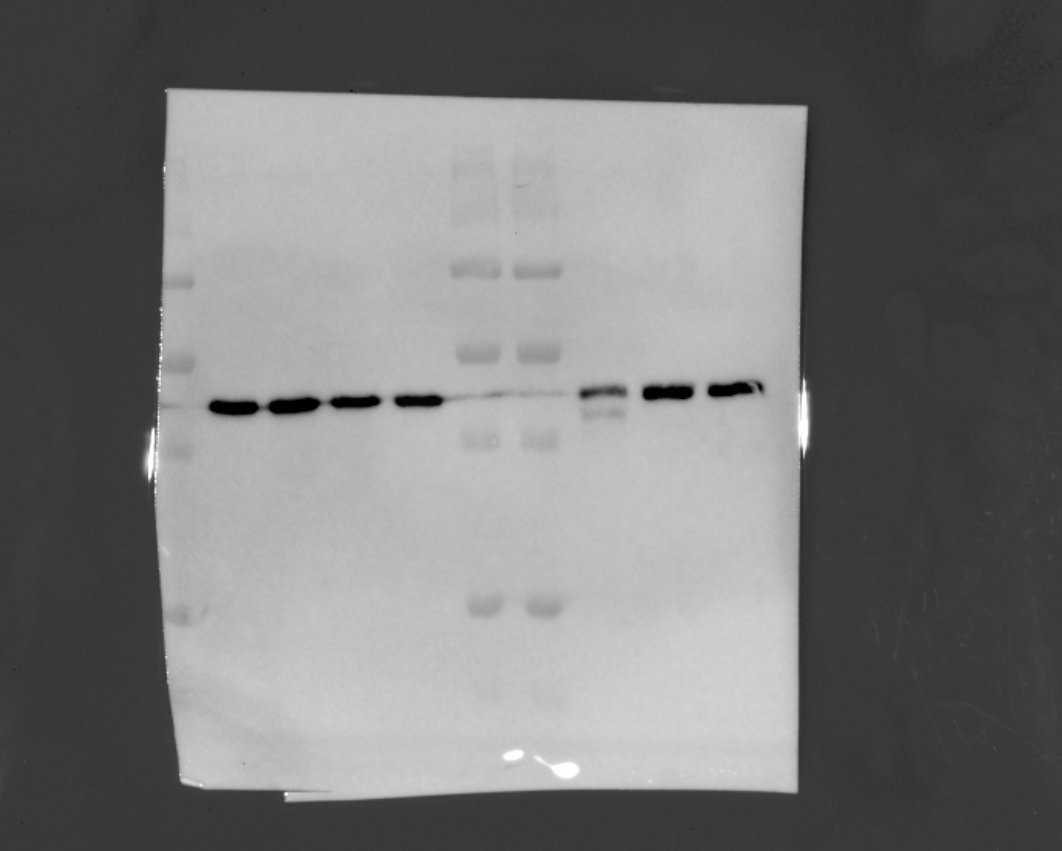

Supplement: Figure 2—figure supplement 1—source data 2. [file elife-97019-fig2-figsupp1-data2.zip › Figure 2-figure supplement 1, source data 2/Actin.tif]

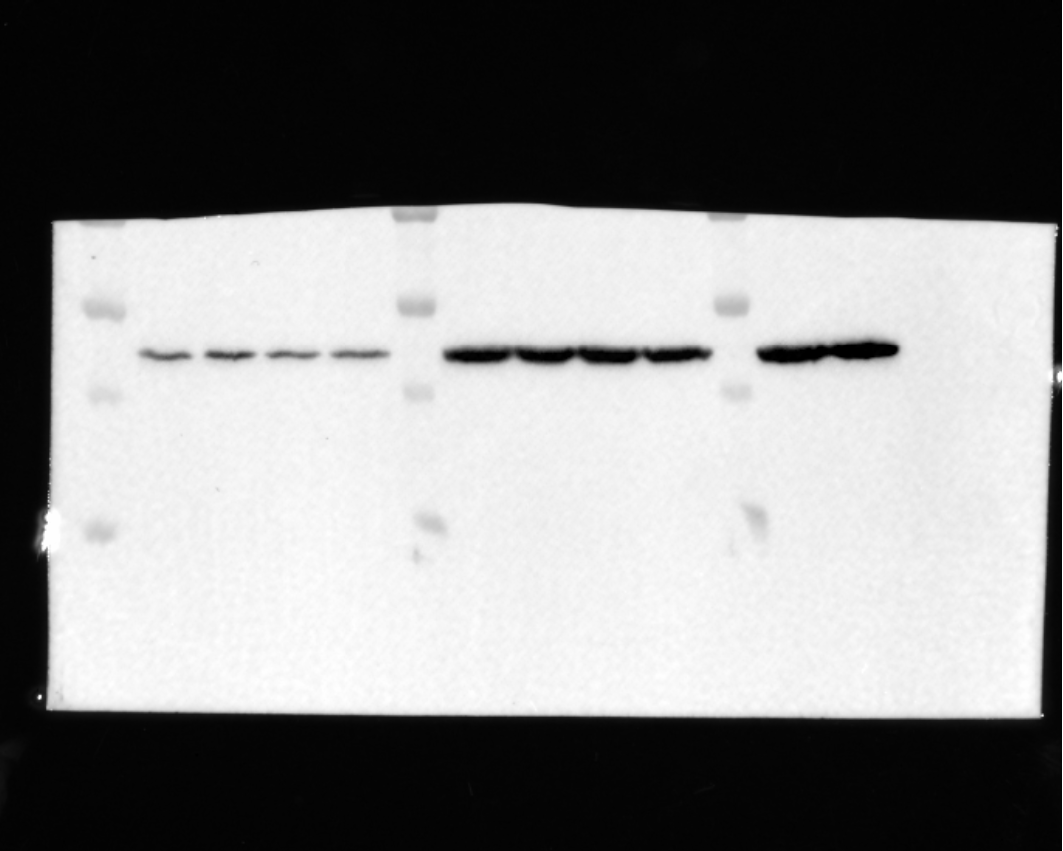

Supplement: Figure 2—figure supplement 2—source data 2. [file elife-97019-fig2-figsupp2-data2.zip › Figure 2-figure supplement 2 cource data 2/Actin_2.tif]

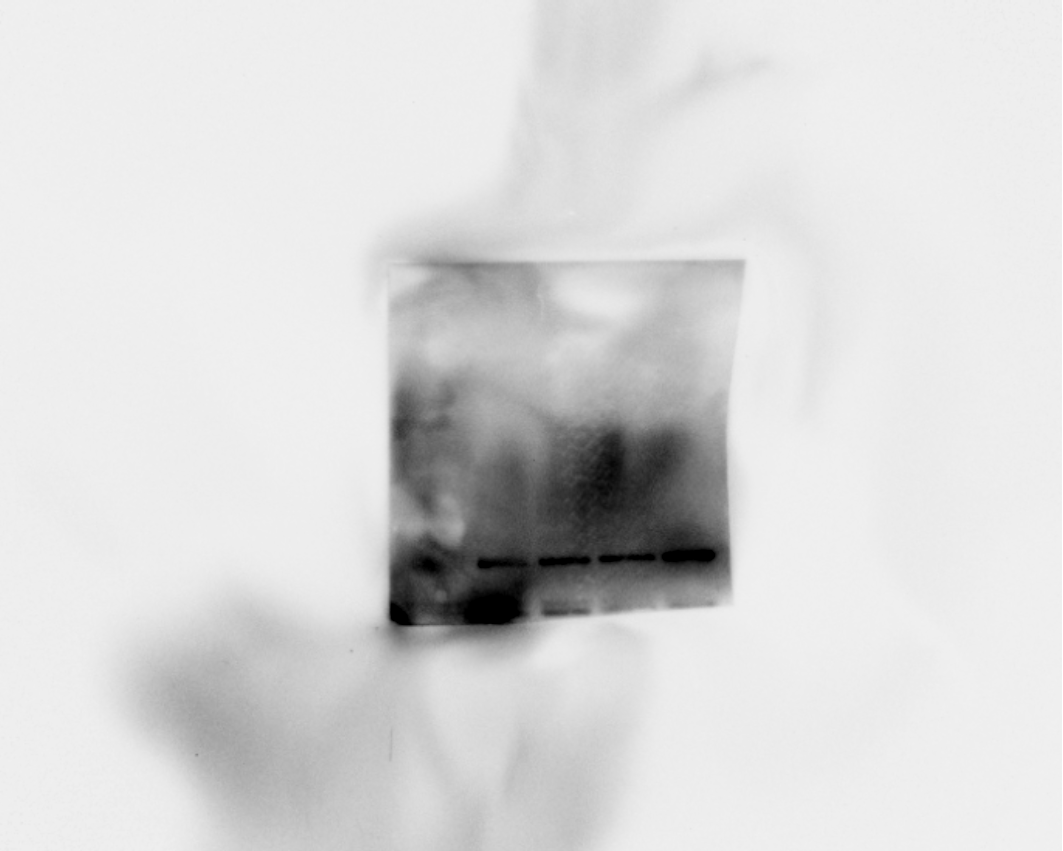

Supplement: Figure 2—figure supplement 2—source data 2. [file elife-97019-fig2-figsupp2-data2.zip › Figure 2-figure supplement 2 cource data 2/ACSS2_2.tif]

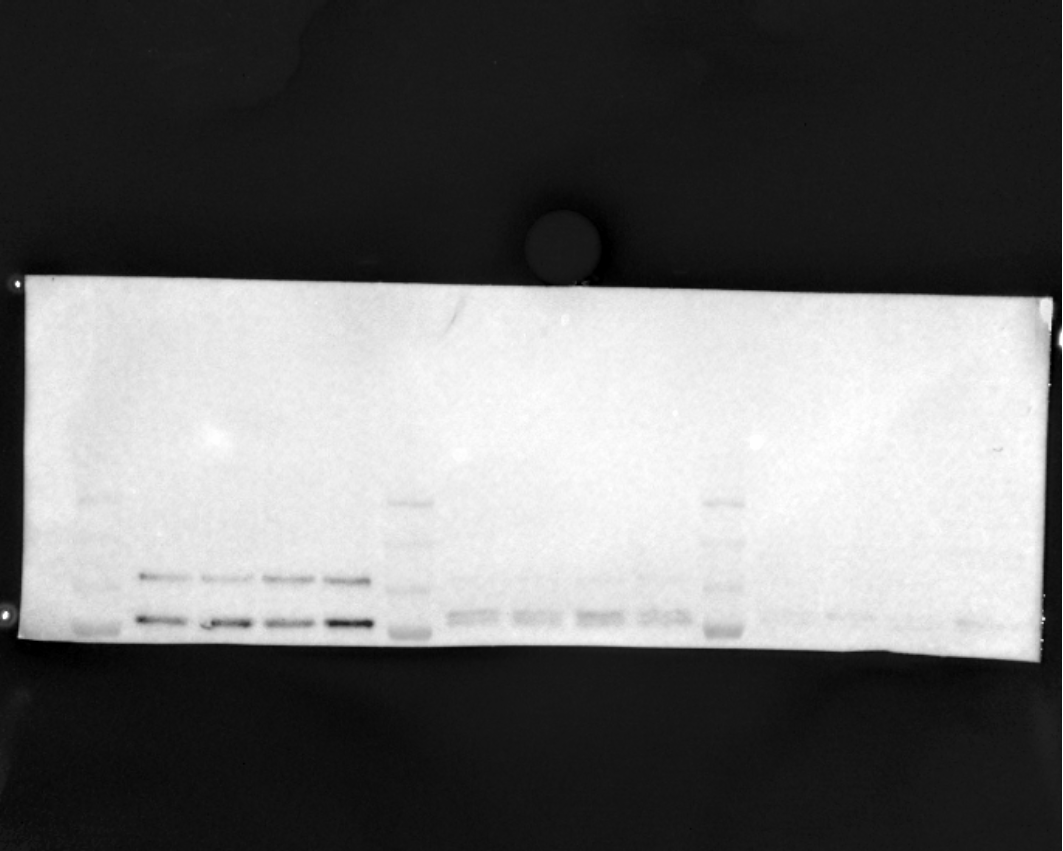

Supplement: Figure 2—figure supplement 2—source data 2. [file elife-97019-fig2-figsupp2-data2.zip › Figure 2-figure supplement 2 cource data 2/ACSS2.tif]

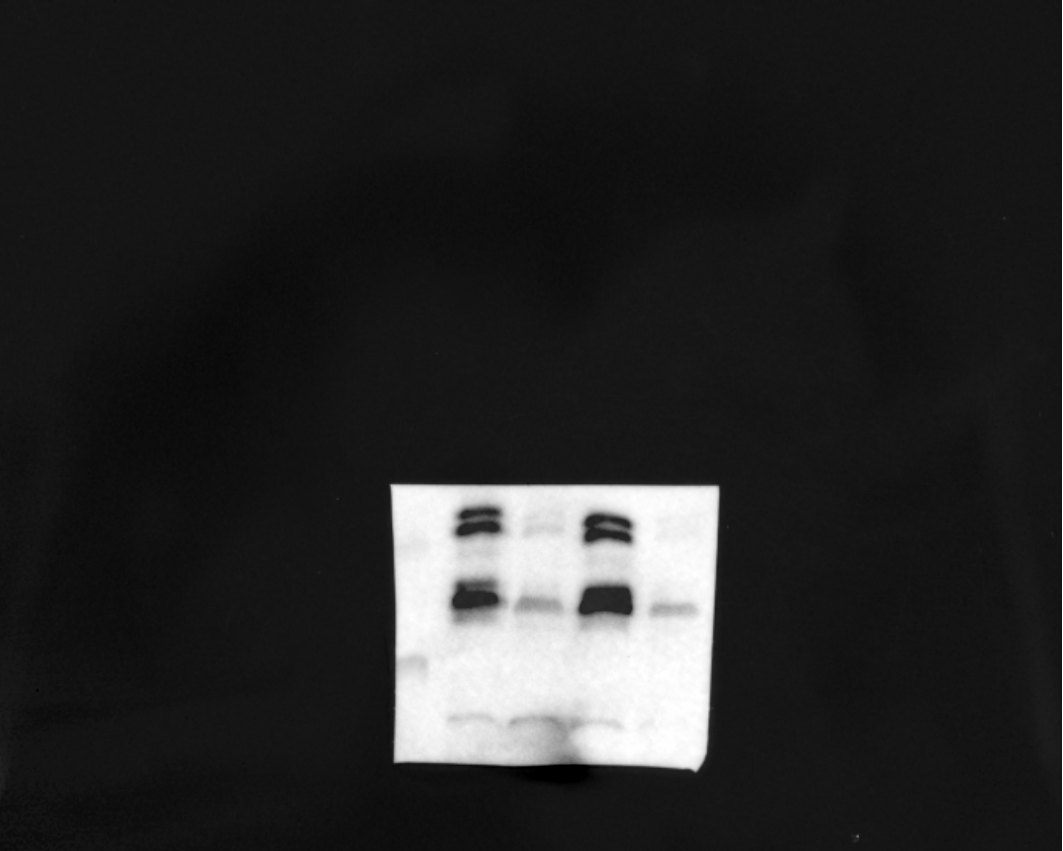

Supplement: Figure 2—figure supplement 2—source data 2. [file elife-97019-fig2-figsupp2-data2.zip › Figure 2-figure supplement 2 cource data 2/SIRT2.tif]

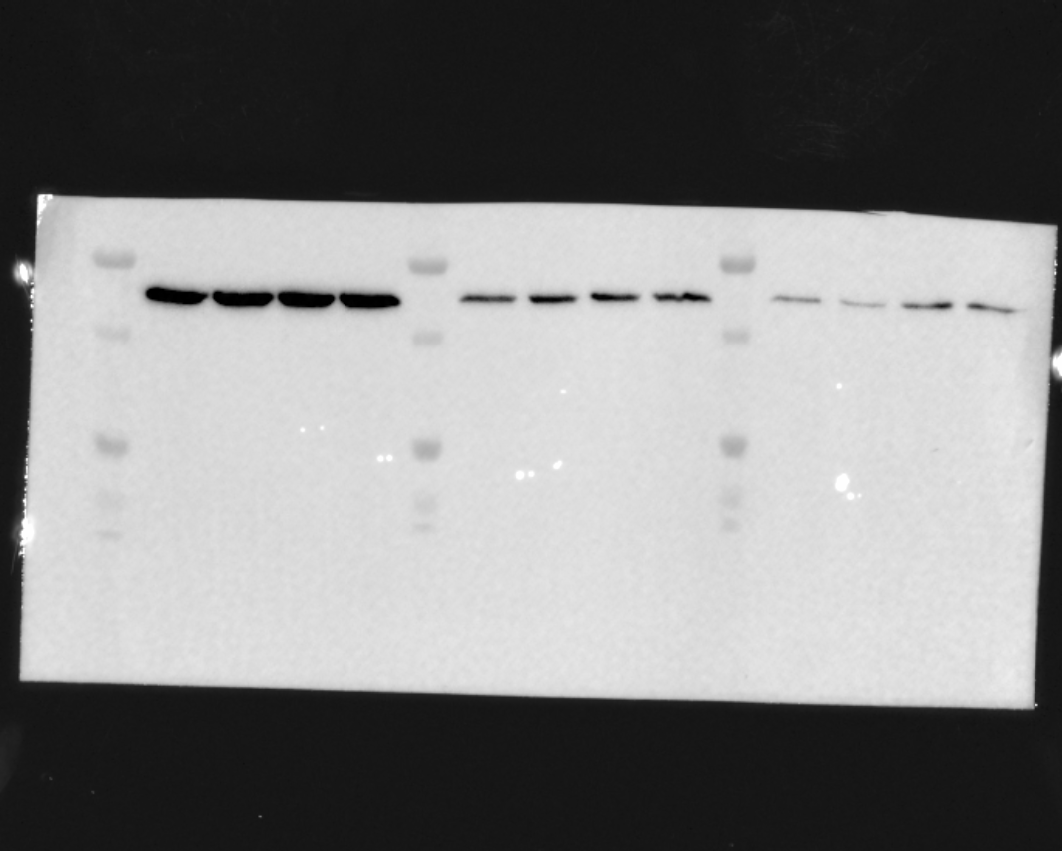

Supplement: Figure 2—figure supplement 2—source data 2. [file elife-97019-fig2-figsupp2-data2.zip › Figure 2-figure supplement 2 cource data 2/Actin.tif]

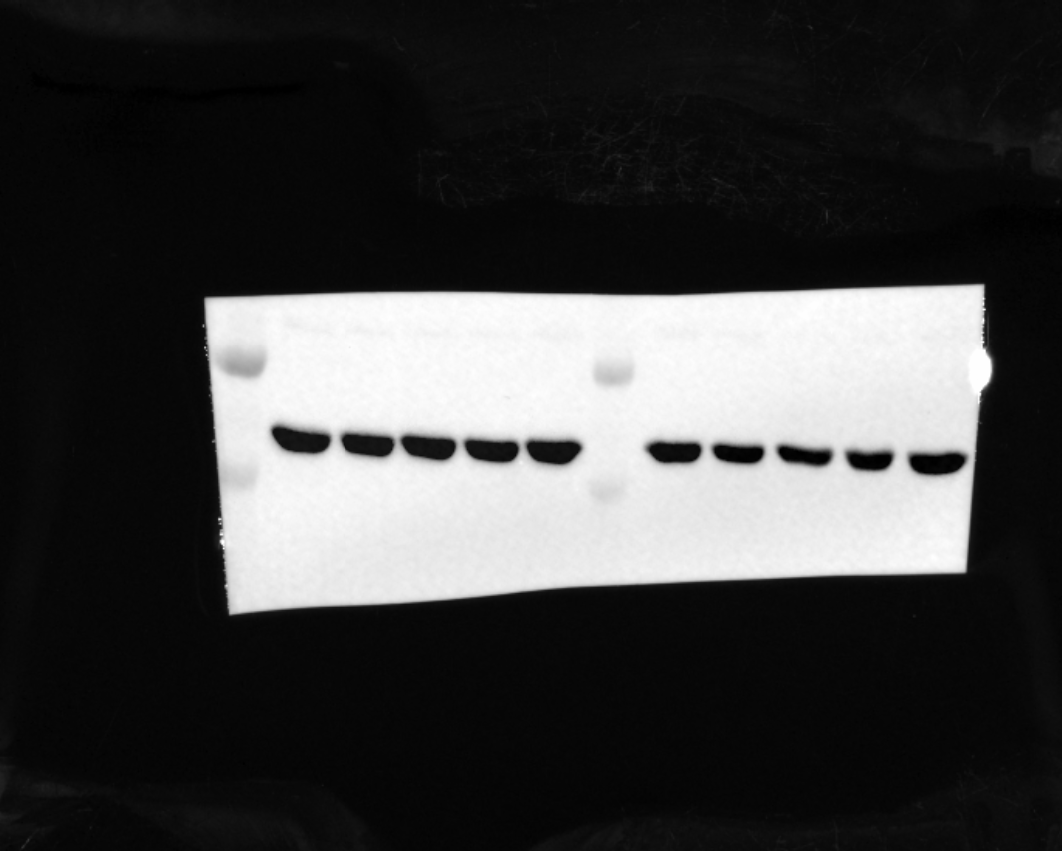

Supplement: Figure 2—figure supplement 3—source data 2. [file elife-97019-fig2-figsupp3-data2.zip › Figure 2-figure supplement 3 source data 2/Actin.tif]

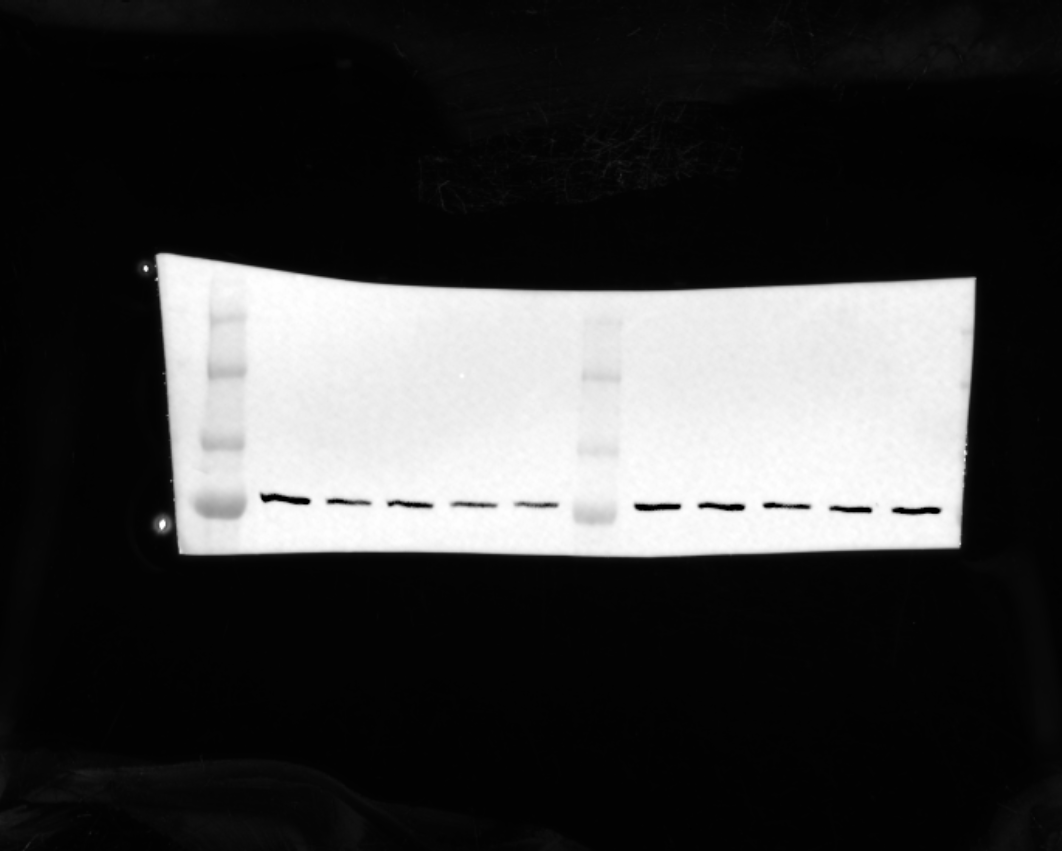

Supplement: Figure 2—figure supplement 3—source data 2. [file elife-97019-fig2-figsupp3-data2.zip › Figure 2-figure supplement 3 source data 2/ACSS2.tif]

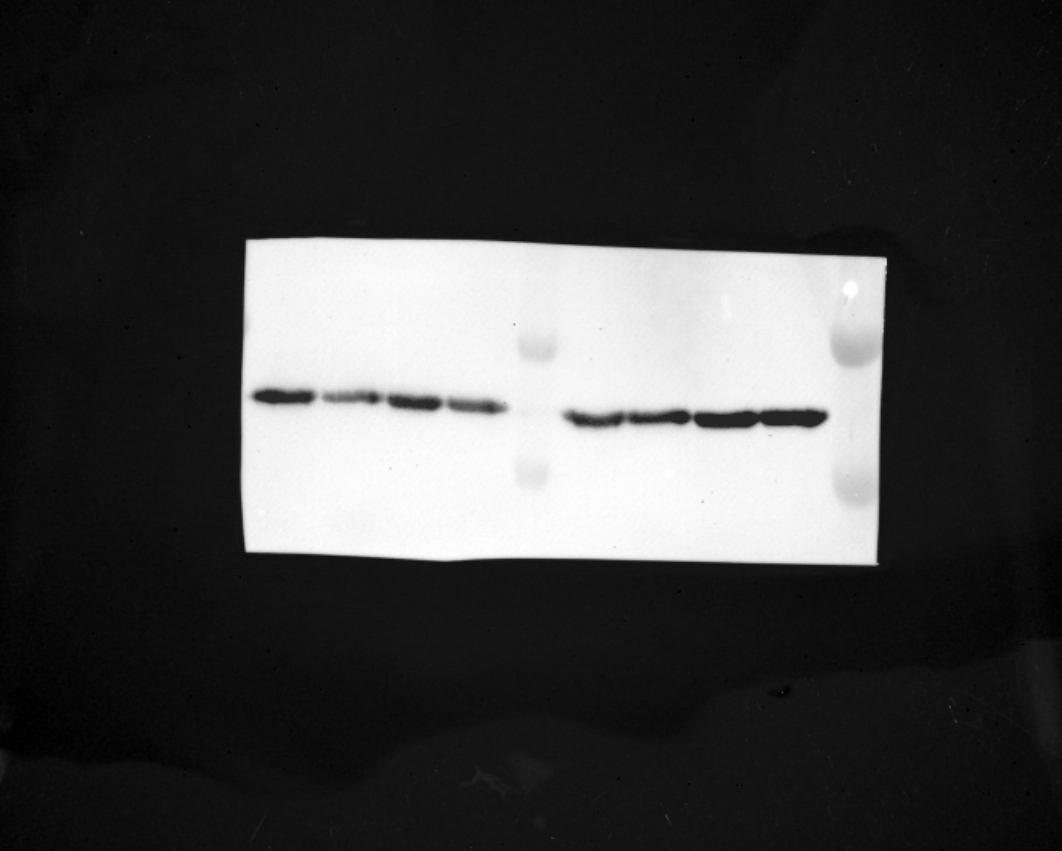

Supplement: Figure 2—figure supplement 6—source data 2. [file elife-97019-fig2-figsupp6-data2.zip › Figure 2-figure supplement 6, Source Data 2/Actin.tif]

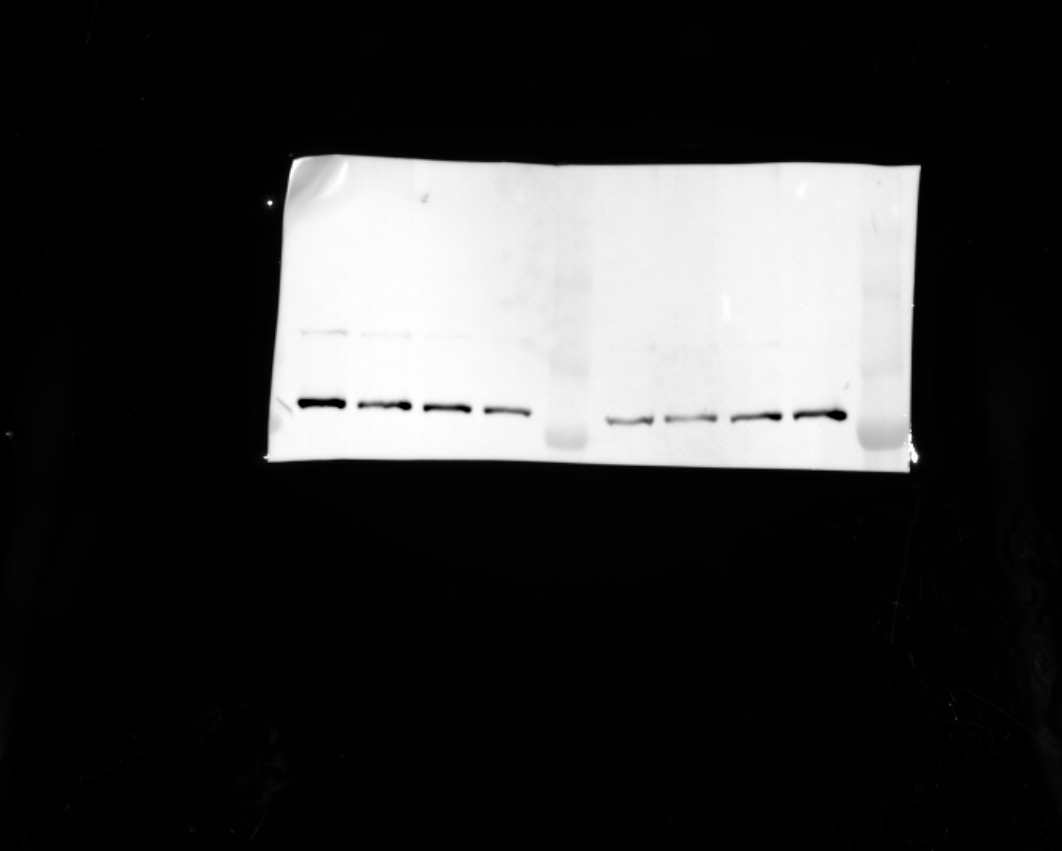

Supplement: Figure 2—figure supplement 6—source data 2. [file elife-97019-fig2-figsupp6-data2.zip › Figure 2-figure supplement 6, Source Data 2/ACSS2.tif]

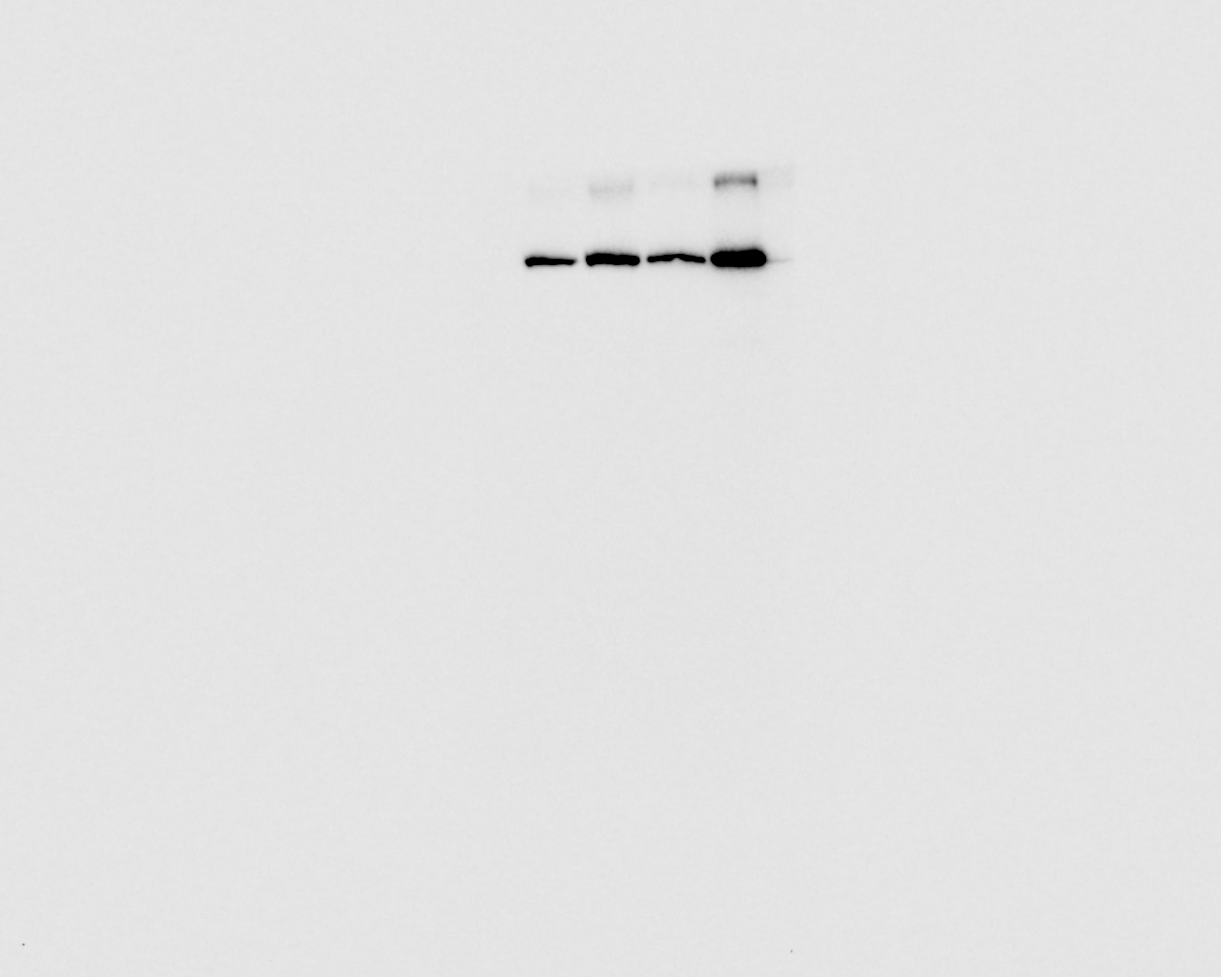

Supplement: Figure 2—figure supplement 8—source data 2. [file elife-97019-fig2-figsupp8-data2.zip › Figure 2-figure supplement 8, Source Data 2/IP.tif]

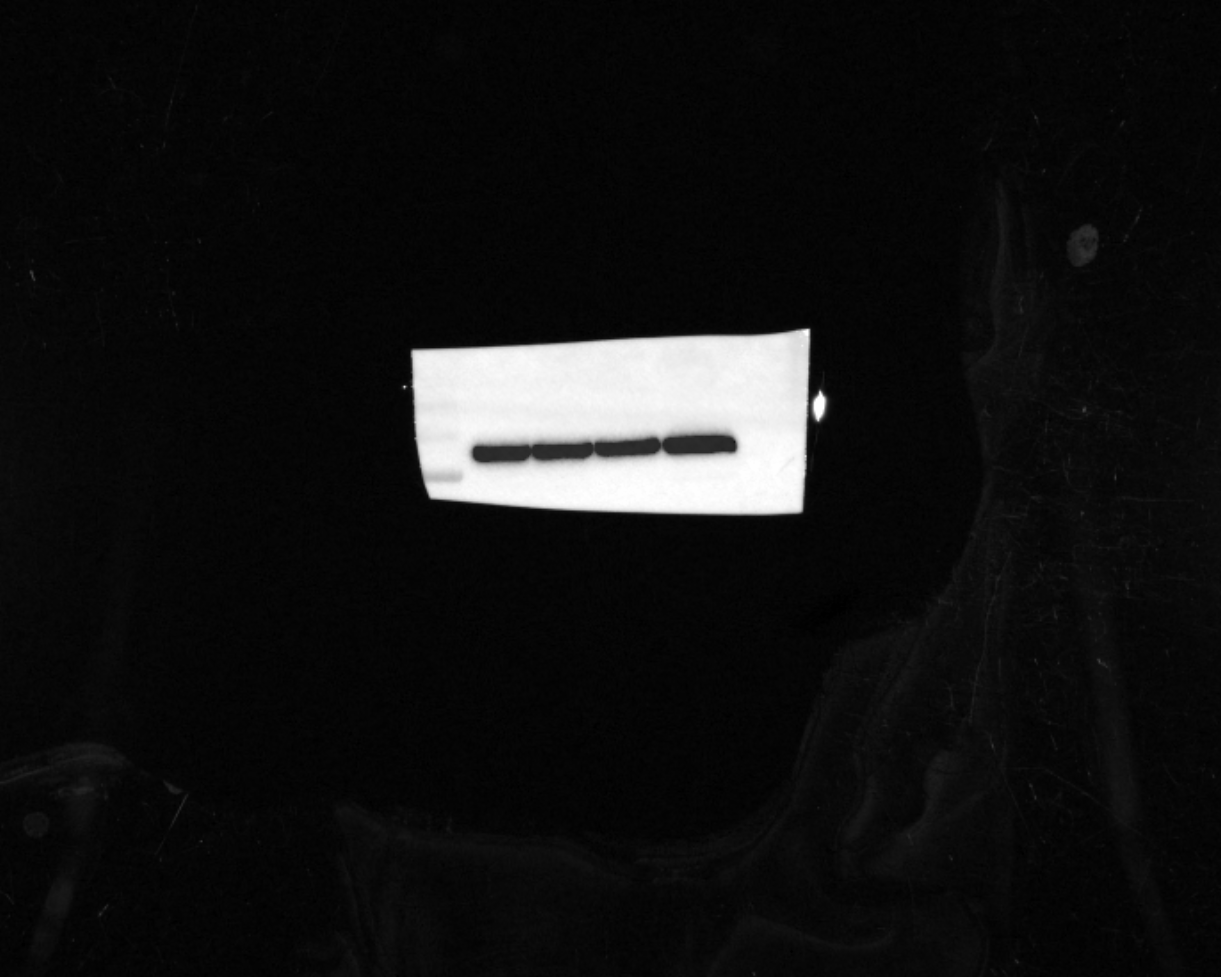

Supplement: Figure 2—figure supplement 8—source data 2. [file elife-97019-fig2-figsupp8-data2.zip › Figure 2-figure supplement 8, Source Data 2/flag.tif]

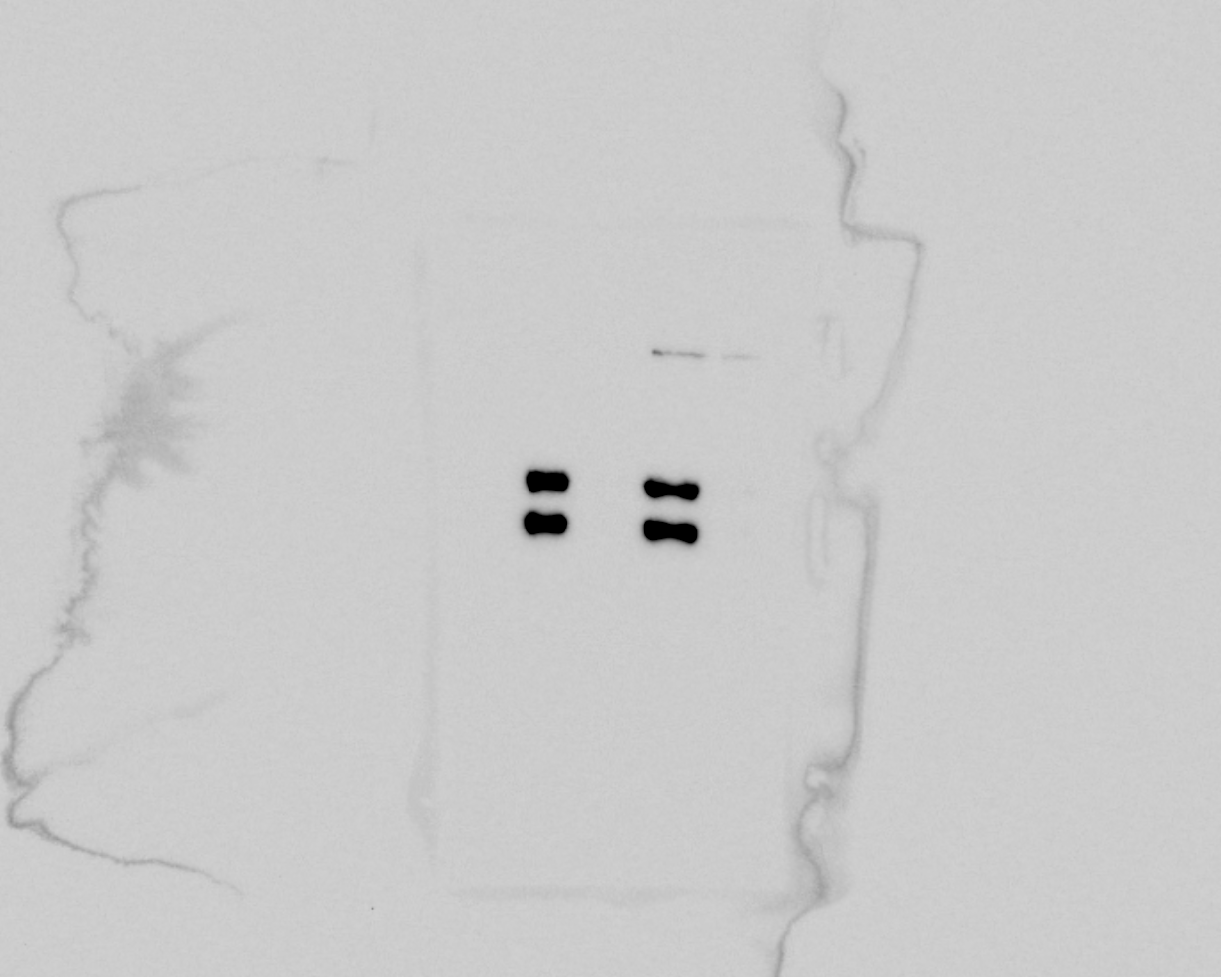

Supplement: Figure 2—figure supplement 8—source data 2. [file elife-97019-fig2-figsupp8-data2.zip › Figure 2-figure supplement 8, Source Data 2/SIRT2.tif]

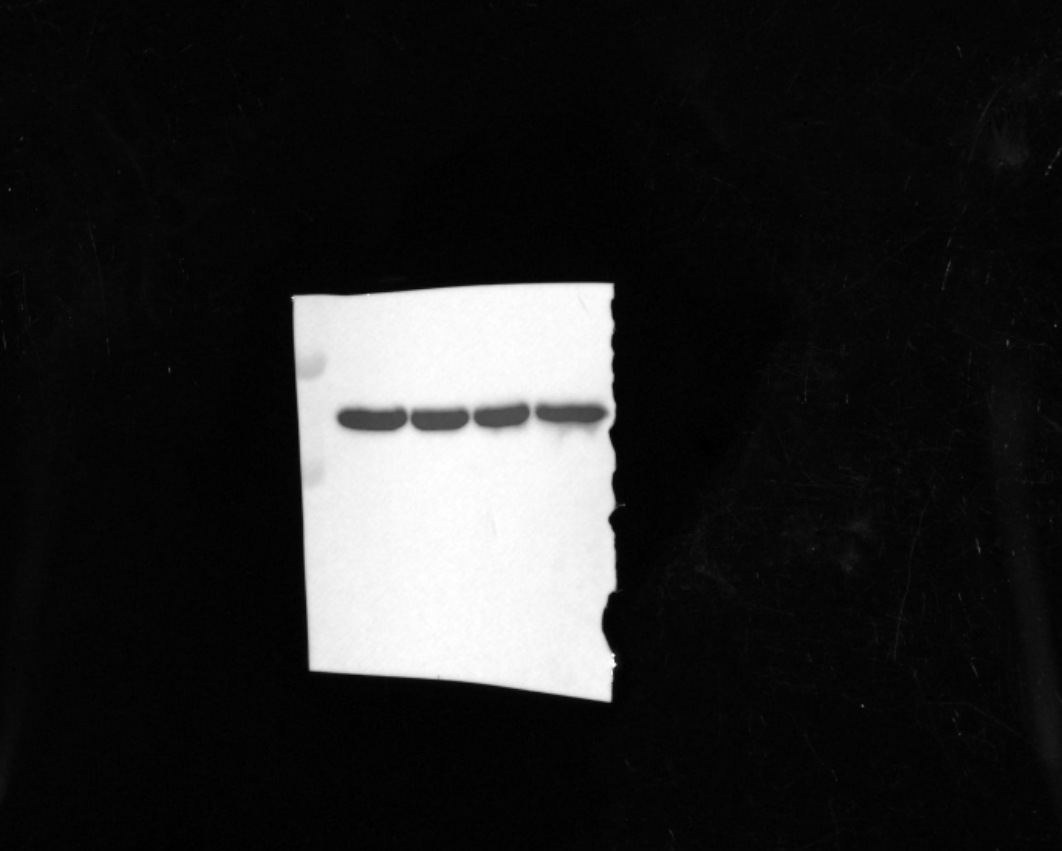

Supplement: Figure 2—figure supplement 8—source data 2. [file elife-97019-fig2-figsupp8-data2.zip › Figure 2-figure supplement 8, Source Data 2/actin.tif]

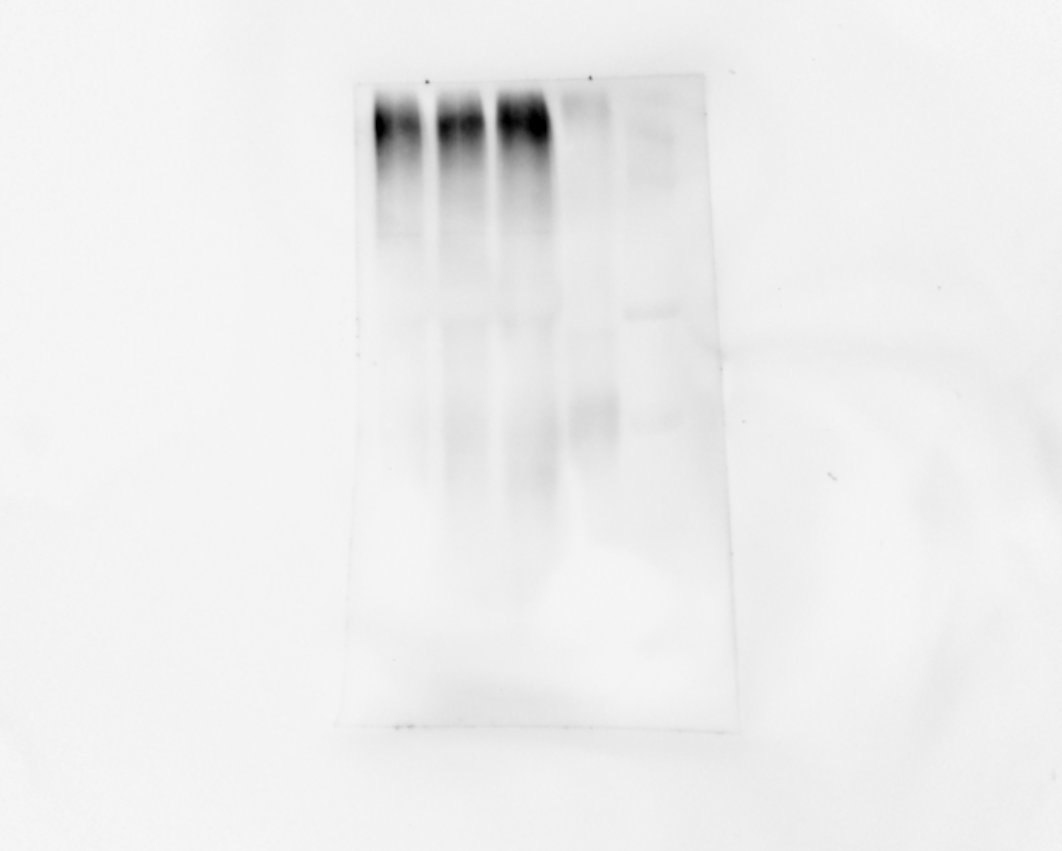

Supplement: Figure 2—figure supplement 9—source data 2. [file elife-97019-fig2-figsupp9-data2.zip › Figure 2-figure supplement 9, Source Data 2/Ub.tif]

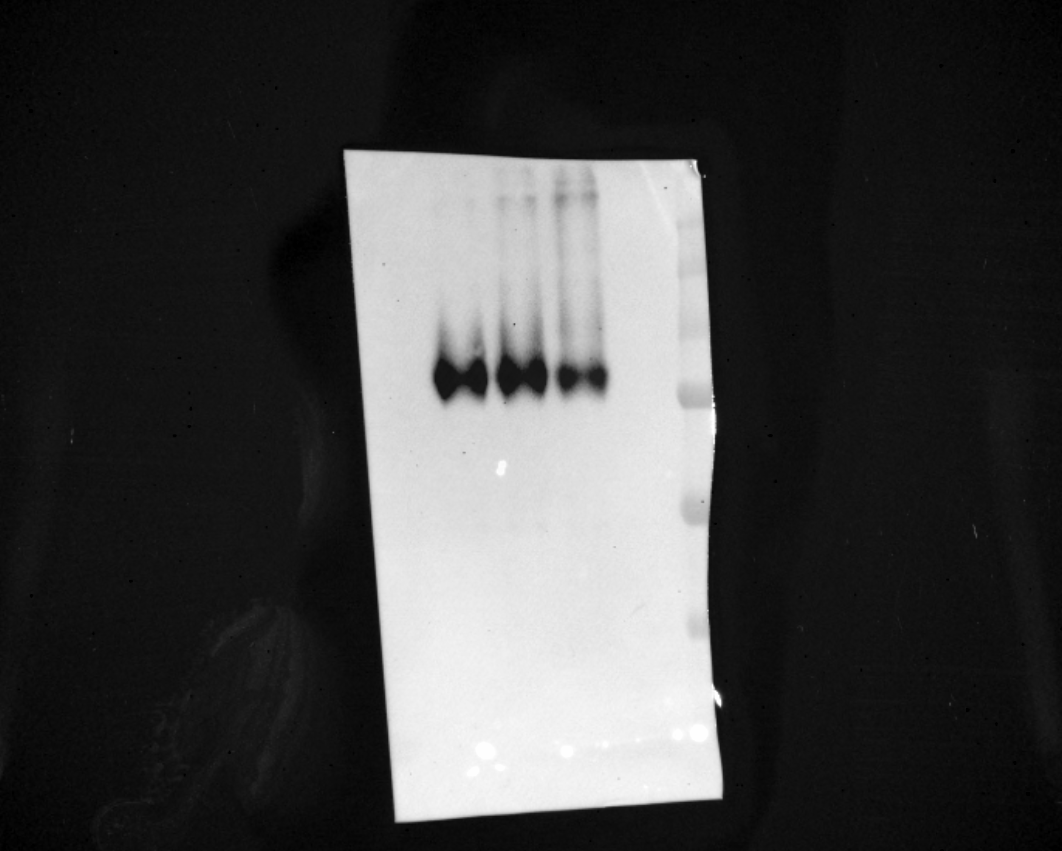

Supplement: Figure 2—figure supplement 9—source data 2. [file elife-97019-fig2-figsupp9-data2.zip › Figure 2-figure supplement 9, Source Data 2/IP.tif]

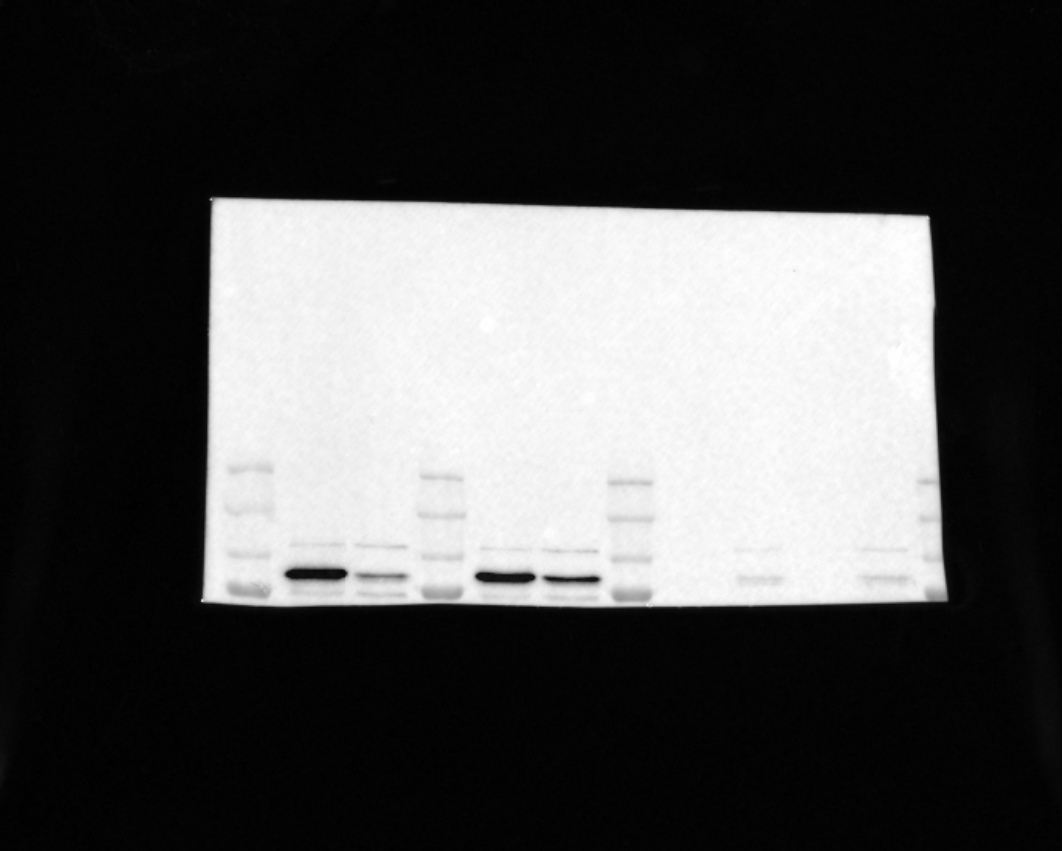

Supplement: Figure 3—source data 2. [file elife-97019-fig3-data2.zip › Figure 3-source data 2/3B/WT_7_ACSS2.tif]

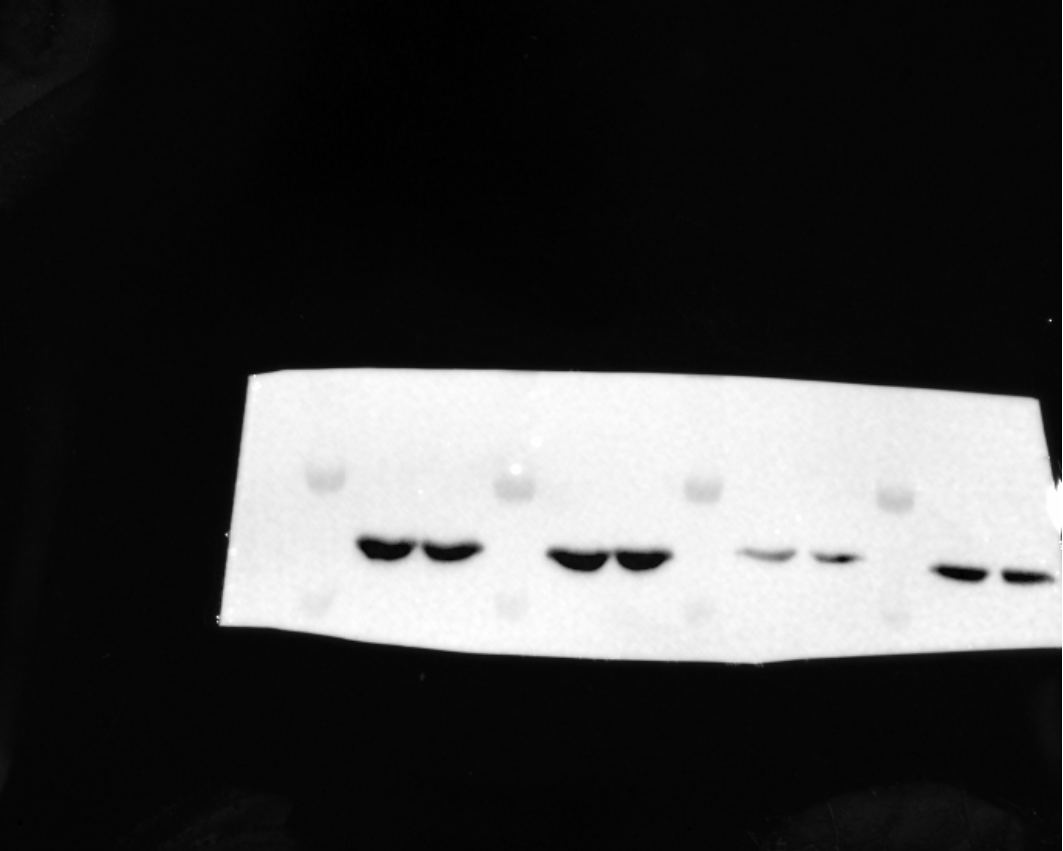

Supplement: Figure 3—source data 2. [file elife-97019-fig3-data2.zip › Figure 3-source data 2/3B/Actin_2.tif]

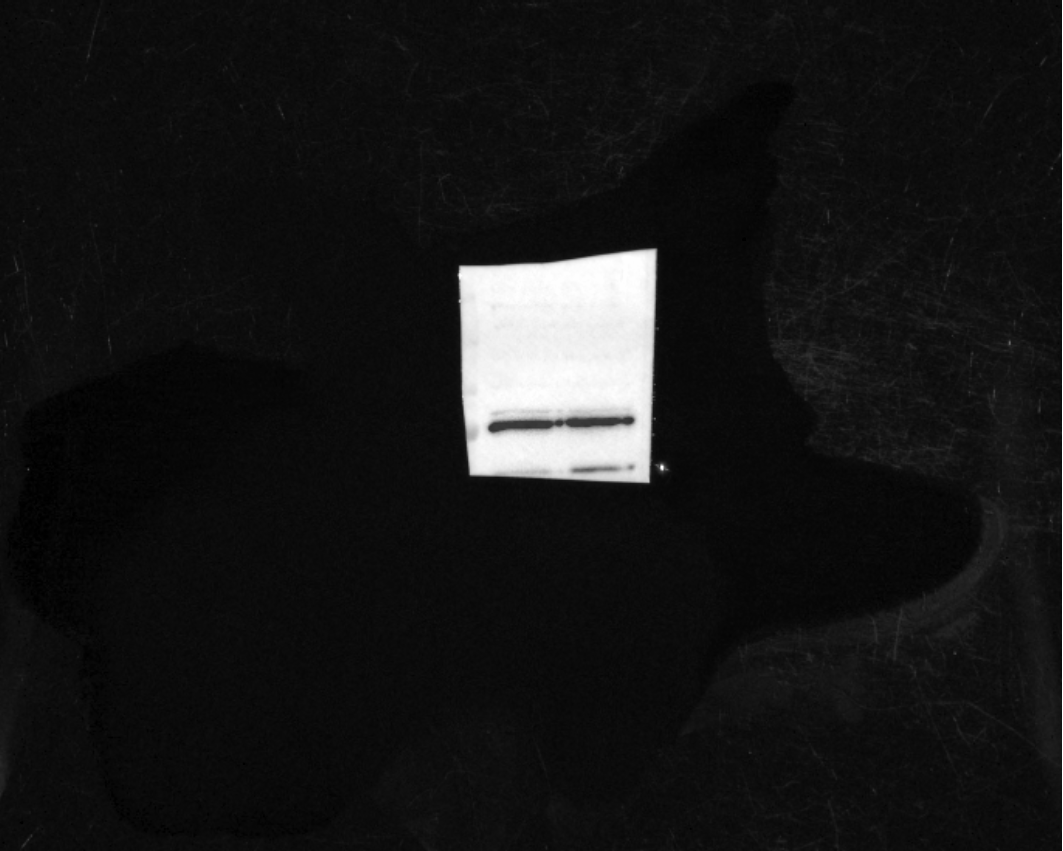

Supplement: Figure 3—source data 2. [file elife-97019-fig3-data2.zip › Figure 3-source data 2/3B/wt_0_acss2.tif]

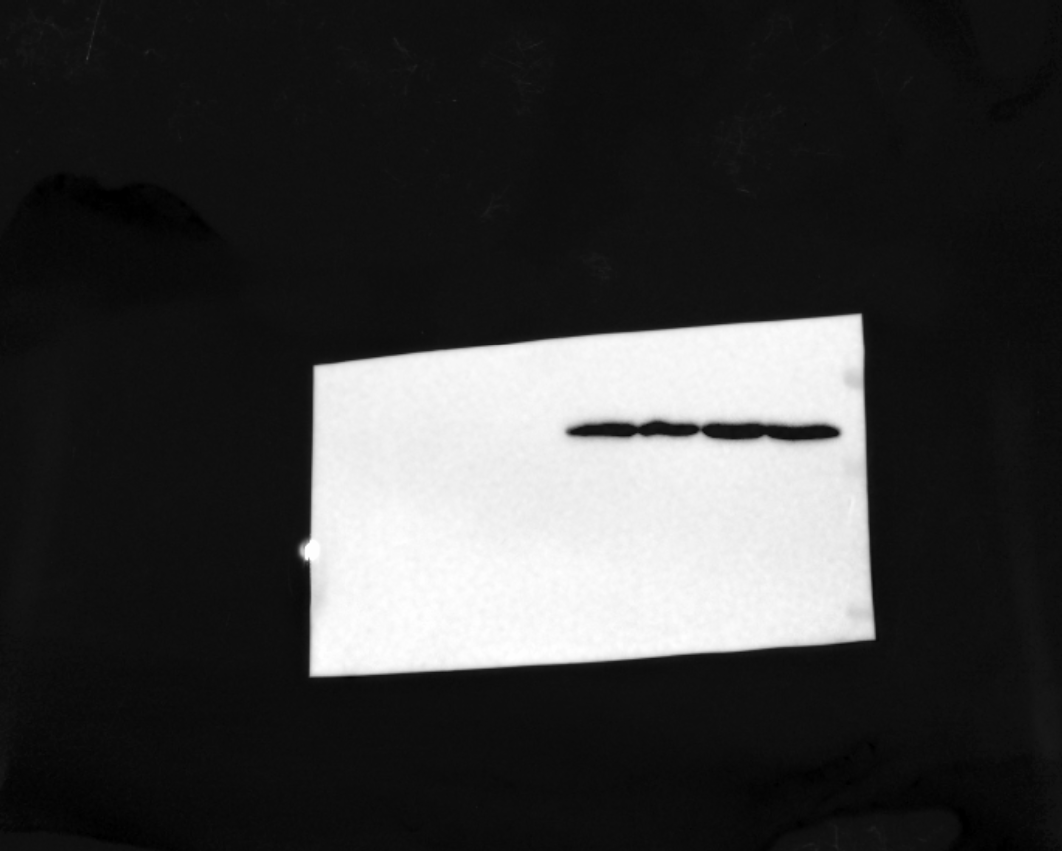

Supplement: Figure 3—source data 2. [file elife-97019-fig3-data2.zip › Figure 3-source data 2/3A/Actin.tif]

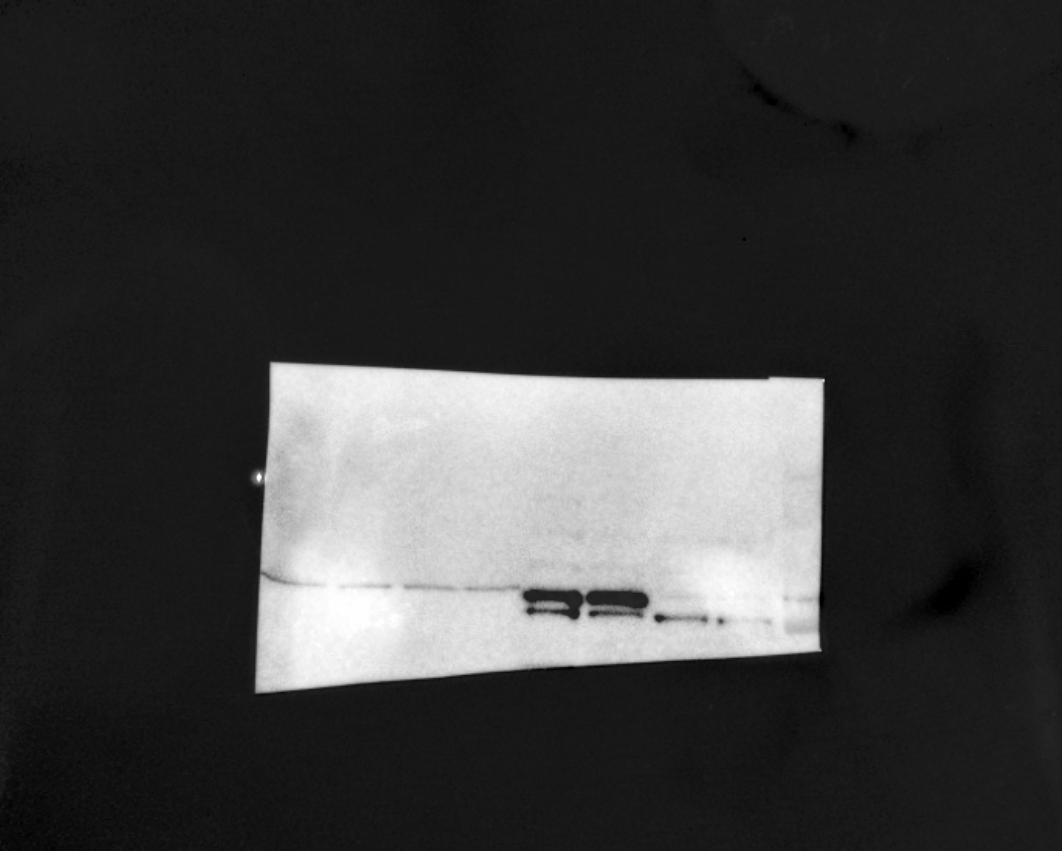

Supplement: Figure 3—source data 2. [file elife-97019-fig3-data2.zip › Figure 3-source data 2/3A/Flag.tif]

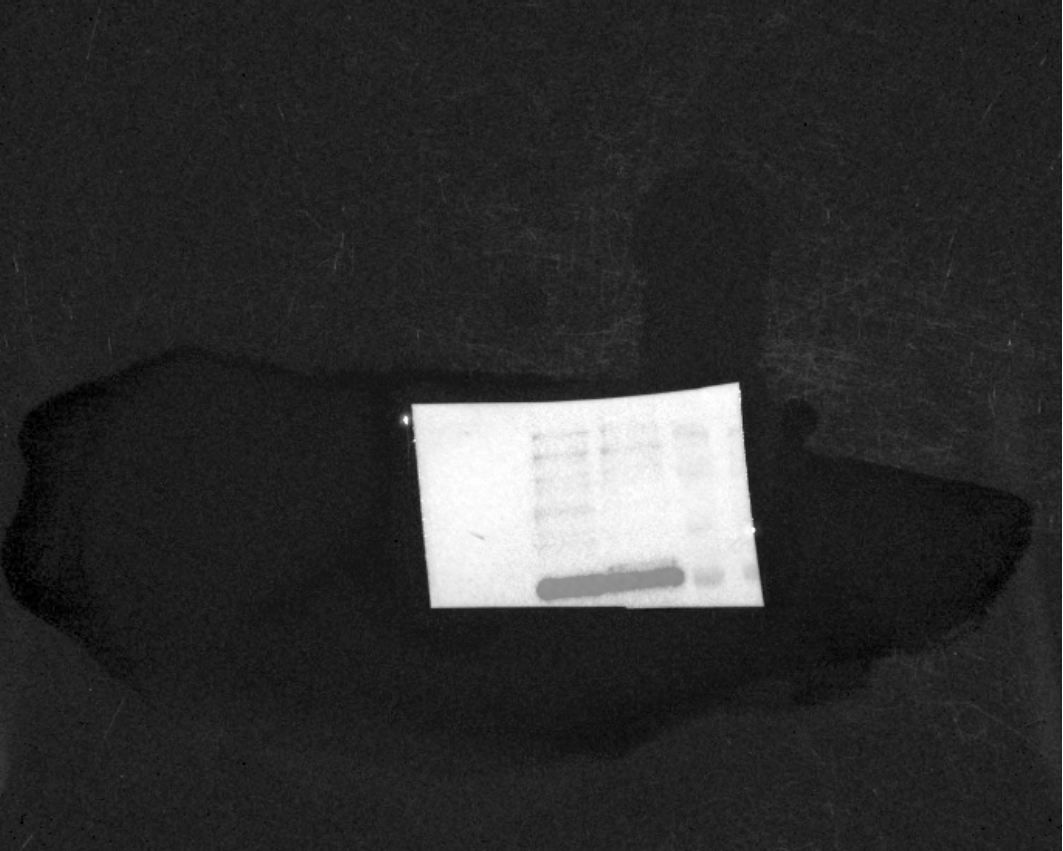

Supplement: Figure 3—source data 2. [file elife-97019-fig3-data2.zip › Figure 3-source data 2/3B/WT_0_flag.tif]

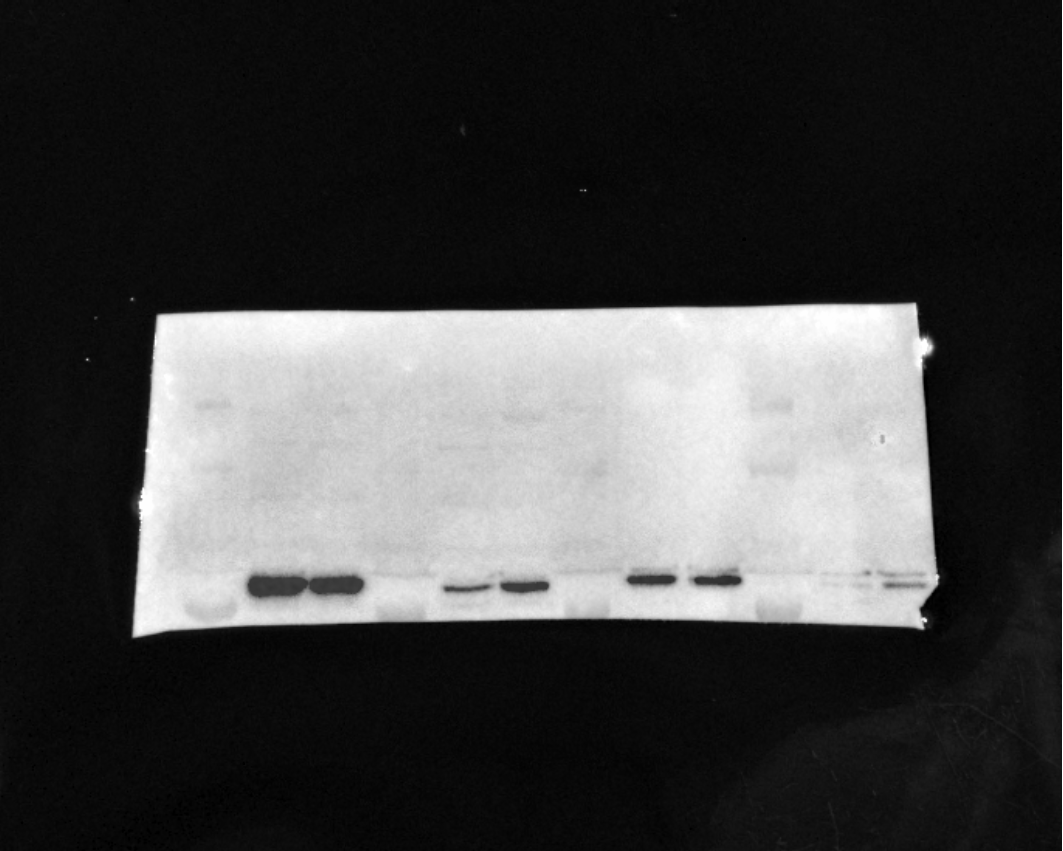

Supplement: Figure 3—source data 2. [file elife-97019-fig3-data2.zip › Figure 3-source data 2/3B/Flag.tif]

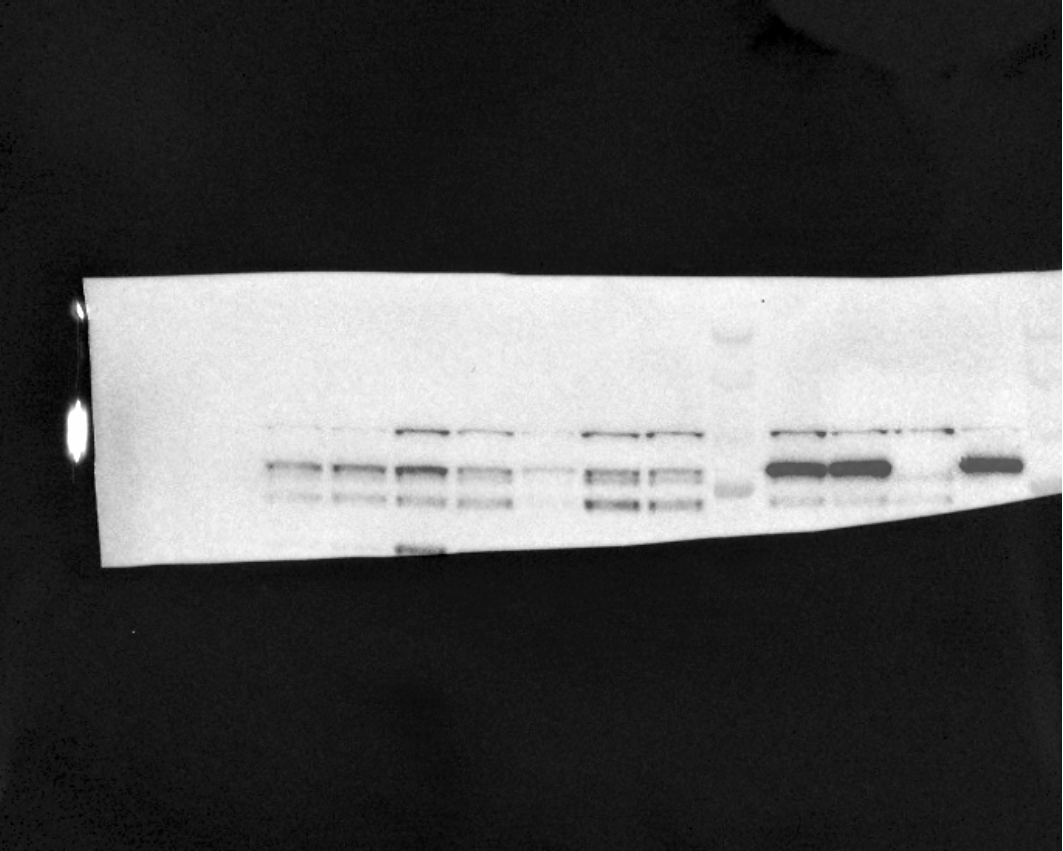

Supplement: Figure 3—source data 2. [file elife-97019-fig3-data2.zip › Figure 3-source data 2/3A/ACSS2.tif]

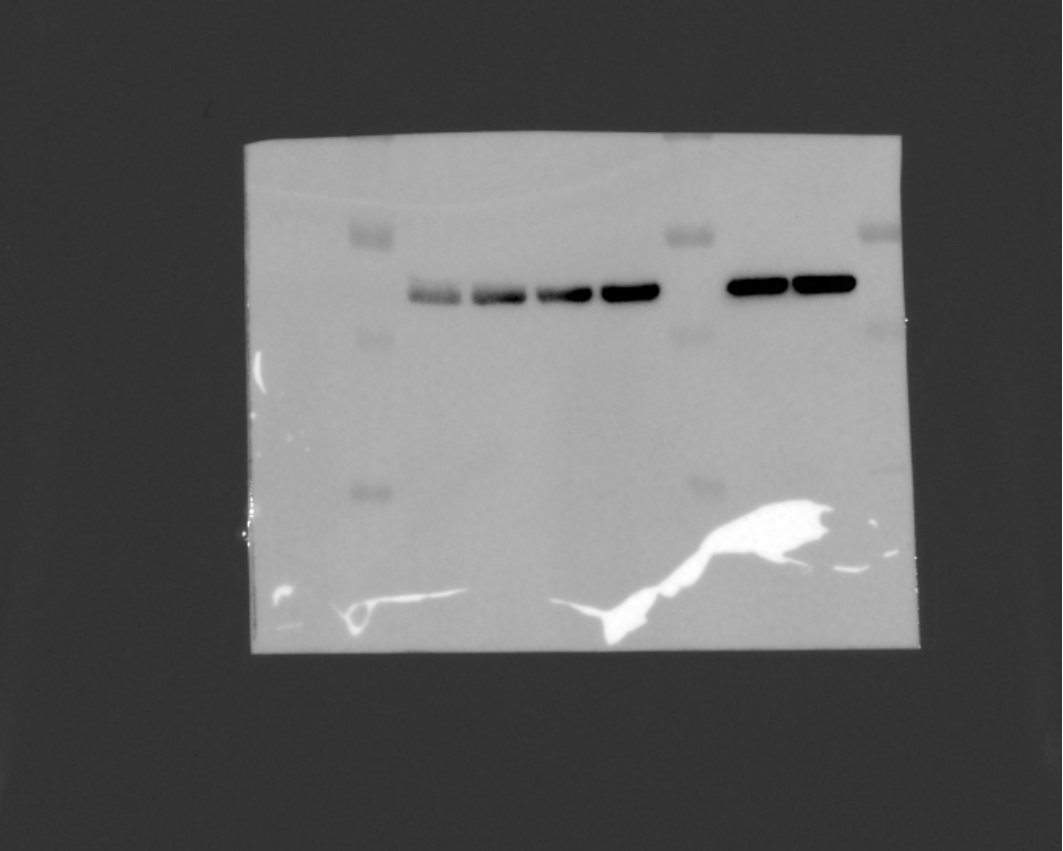

Supplement: Figure 3—figure supplement 1—source data 2. [file elife-97019-fig3-figsupp1-data2.zip › Figure 3-figure supplement 1, Source Data 2/actin.tif]

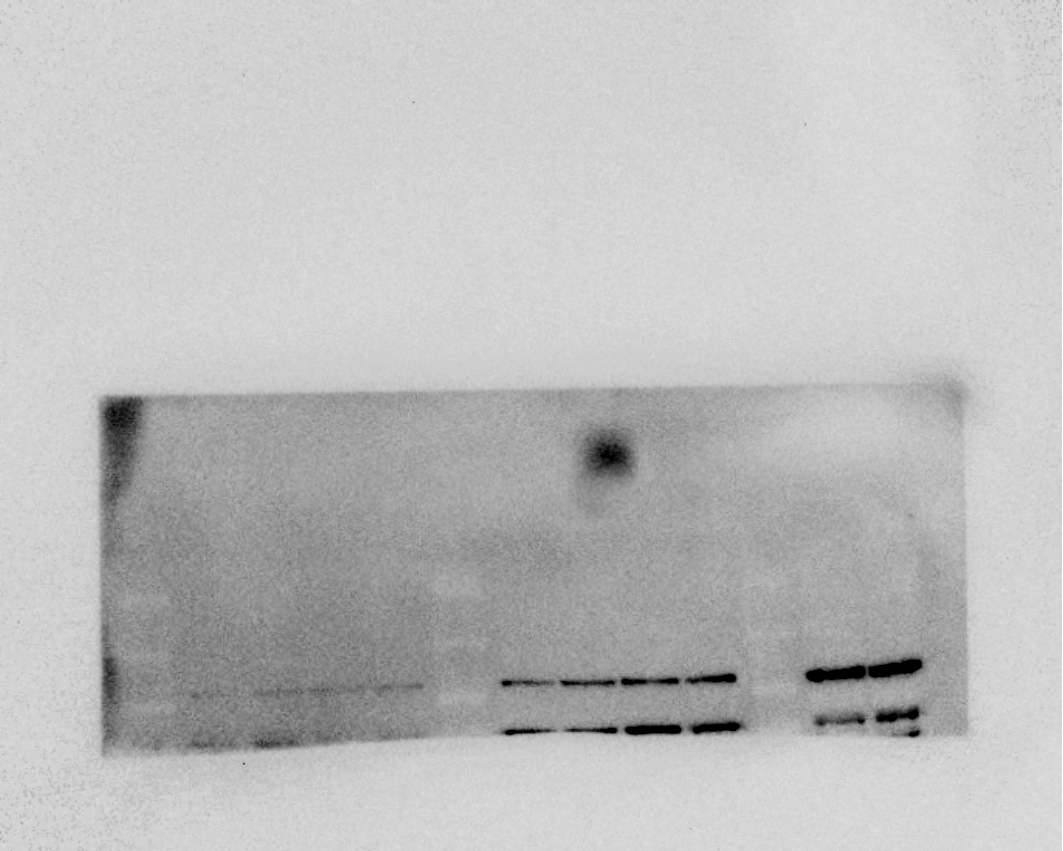

Supplement: Figure 3—figure supplement 1—source data 2. [file elife-97019-fig3-figsupp1-data2.zip › Figure 3-figure supplement 1, Source Data 2/ACSS2.tif]

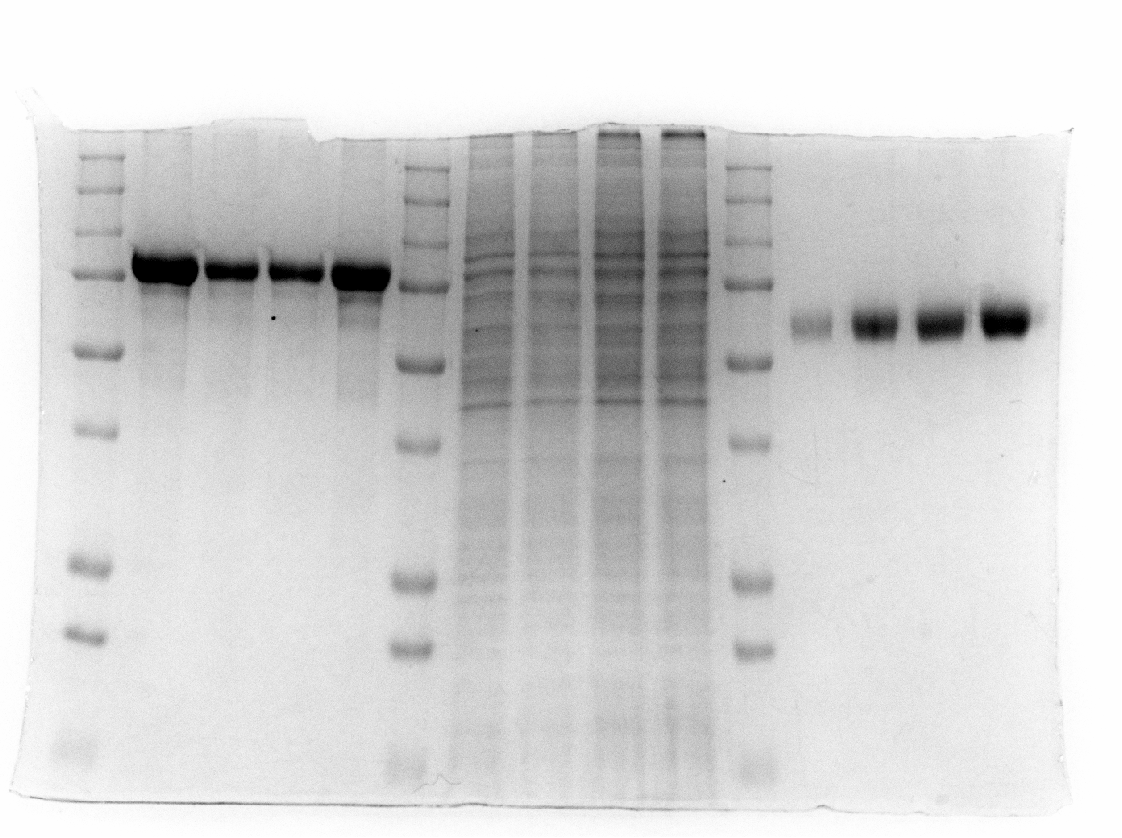

Supplement: Figure 3—figure supplement 2—source data 2. [file elife-97019-fig3-figsupp2-data2.zip › Figure 3-figure supplement 2, Source Data 2/ACSS2 protein purification.tif]
