# Supplementary material for: SIRT2-mediated ACSS2 K271 deacetylation suppresses lipogenesis under nutrient stress
Source: eLife. 2025 May 7;13:RP97019. doi: 10.7554/eLife.97019 (PMC12058118; doi:10.7554/eLife.97019)
Supplement: Figure 1—figure supplement 1—source data 1. [file elife-97019-fig1-figsupp1-data1.zip › Figure 1-figure supplement 1, Source Data 1/Figure 1-figure supplement 1.pdf]

IP: Flag (Alkyne labelled) | IP: Flag (IB:Flag) | Input (IB:Actin) | Input (IB:SIRT2)

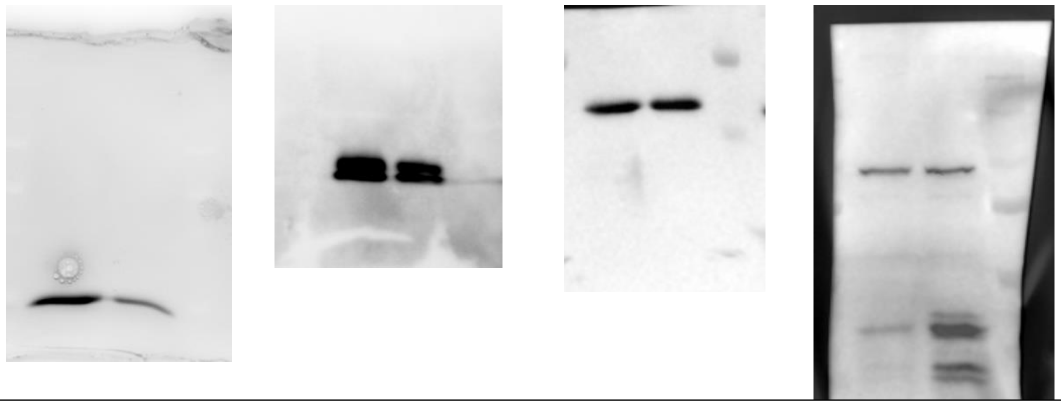

Figure 1-figure supplement 1, Source Data 1. Original membranes corresponding to Figure 1 supplement 1.
