# Supplementary material for: SIRT2-mediated ACSS2 K271 deacetylation suppresses lipogenesis under nutrient stress
Source: eLife. 2025 May 7;13:RP97019. doi: 10.7554/eLife.97019 (PMC12058118; doi:10.7554/eLife.97019)
Supplement: Figure 1—figure supplement 4—source data 1. [file elife-97019-fig1-figsupp4-data1.zip › Figure 1- Figure supplement 4, source data 1/Figure 1-figure supplement 4, source data 1.pdf]

Ac-K IP (IB: Flag)

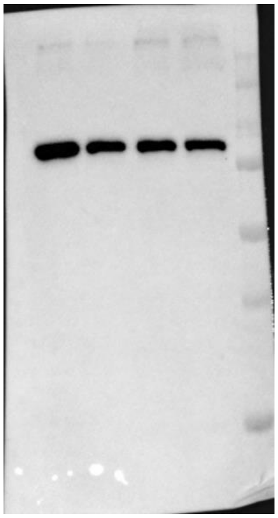

Input (IB: Flag)

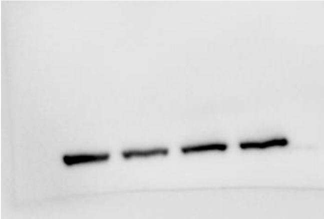

Input (IB: Actin)

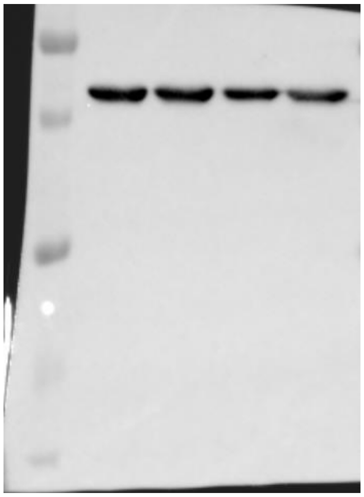

Figure 1-figure supplement 4, Source Data 1. Original membranes corresponding to Figure 1 supplement 4.
