# Supplementary material for: SIRT2-mediated ACSS2 K271 deacetylation suppresses lipogenesis under nutrient stress
Source: eLife. 2025 May 7;13:RP97019. doi: 10.7554/eLife.97019 (PMC12058118; doi:10.7554/eLife.97019)
Supplement: Figure 2—source data 1. [file elife-97019-fig2-data1.zip › Figure 2-source data 1/Figure 2-source data 1_2.pdf]

IP: Flag (IB:Flag)

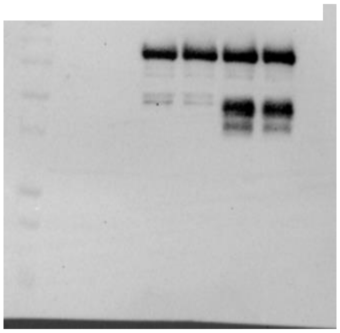

IP: Flag (IB:Ub)

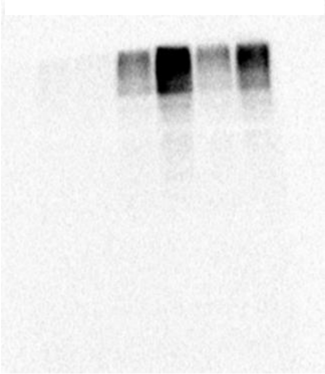

Figure 2, Source Data 1. Original membranes corresponding to Figure 2 E.

IP: Flag (IB: K48)

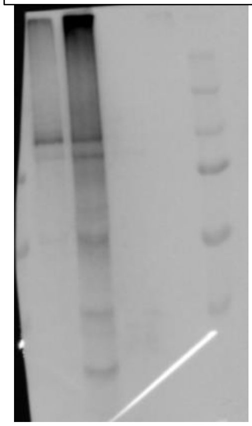

IP: Flag (IB: Flag)

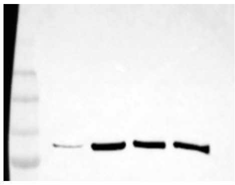

Input (IB: K48)

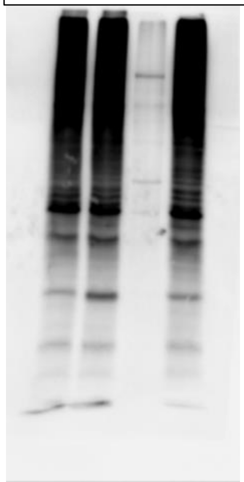

Input (IB: Actin)

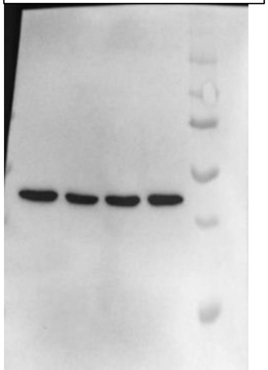

Input (IB: SIRT2)

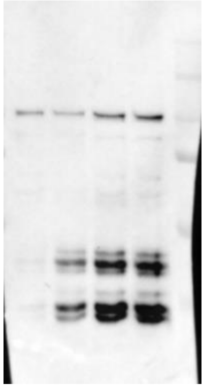

Figure 2, Source Data 1. Original membranes corresponding to Figure 2 F.

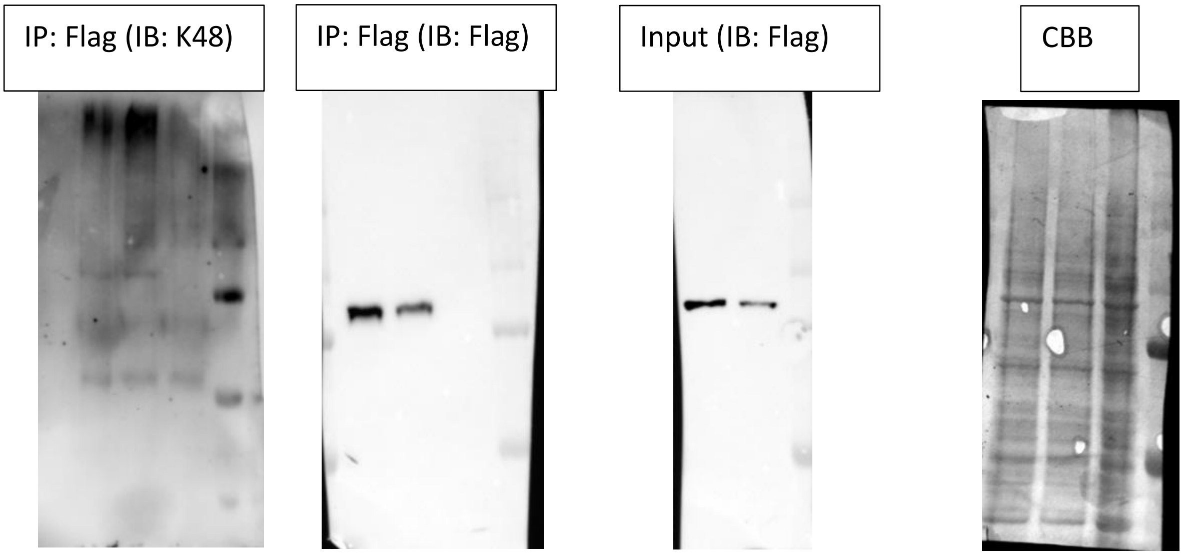

Figure 2, Source Data 1. Original membranes corresponding to Figure 2 G.

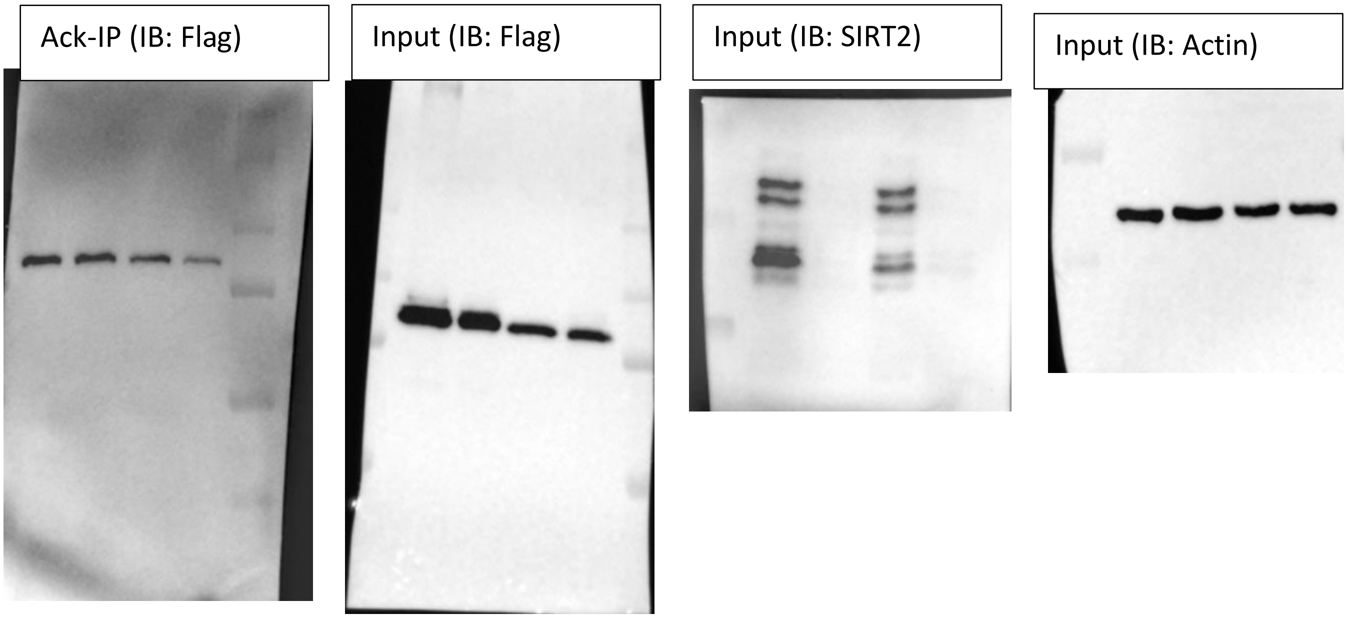

Figure 2, Source Data 1. Original membranes corresponding to Figure 2 H.

IP: Flag (IB: K48)

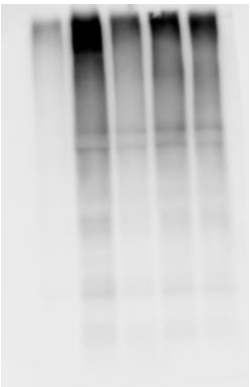

IP: Flag (IB: Flag)

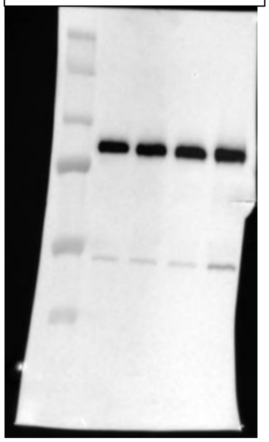

Input (IB: K48)

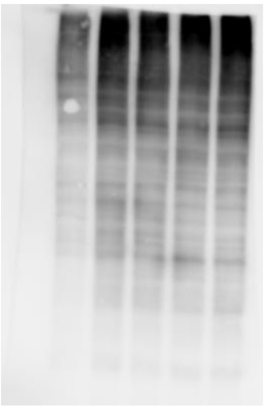

Input (IB: Actin)

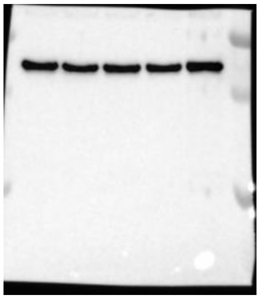

Input (IB: SIRT2)

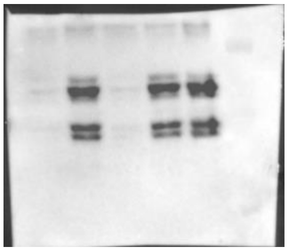

Figure 2, Source Data 1. Original membranes corresponding to Figure 2 I.
