# Supplementary material for: SIRT2-mediated ACSS2 K271 deacetylation suppresses lipogenesis under nutrient stress
Source: eLife. 2025 May 7;13:RP97019. doi: 10.7554/eLife.97019 (PMC12058118; doi:10.7554/eLife.97019)
Supplement: Figure 2—source data 1. [file elife-97019-fig2-data1.zip › Figure 2-source data 1/Figure 2-source data 1..pdf]

IB:ACSS2 IB:Actin IB:SIRT2

75kD

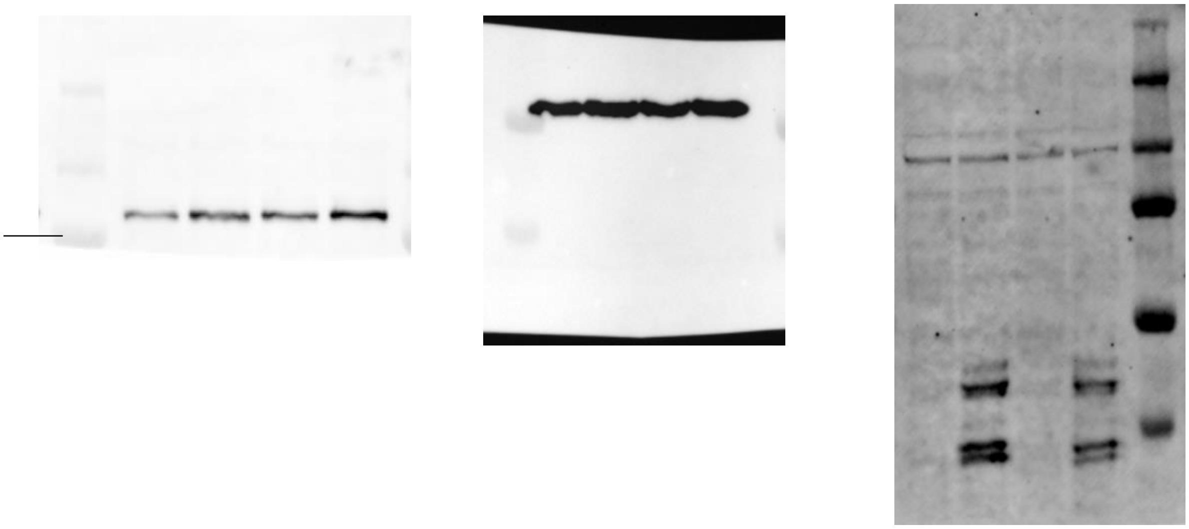

Figure 2, Source Data 1. Original membranes corresponding to Figure 2 A. BioRad protein ladder was used. Molecular weight for ACSS2 (78kD) and SIRT2 (43kD) are labelled.

IB:ACSS2 IB:Actin IB:SIRT2

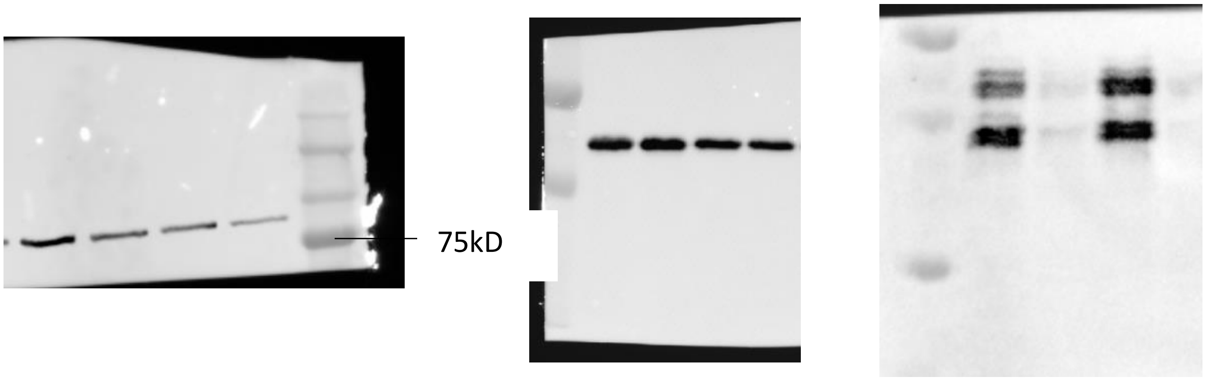

Figure 2, Source Data 1. Original membranes corresponding to Figure 2 B. BioRad protein ladder was used. Molecular weight for ACSS2 (78kD) and SIRT2 (43kD) are labelled.

IB:ACSS2

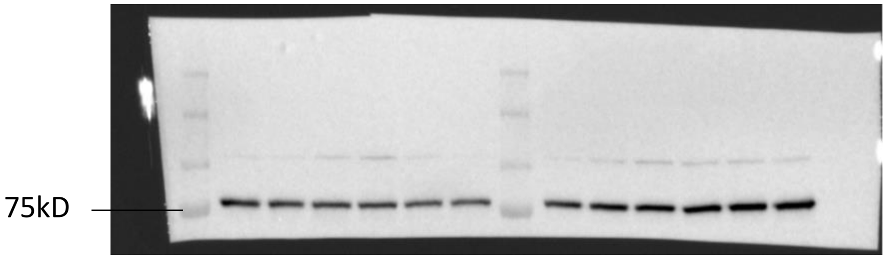

IB:Actin

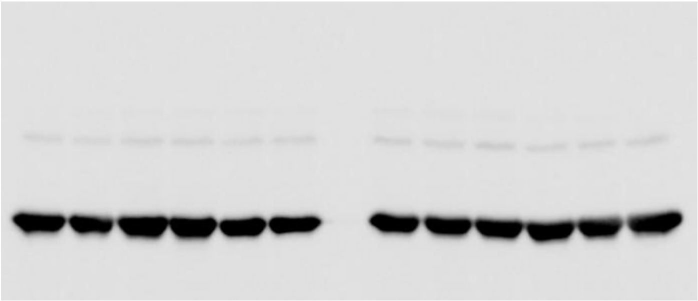

IB:SIRT2

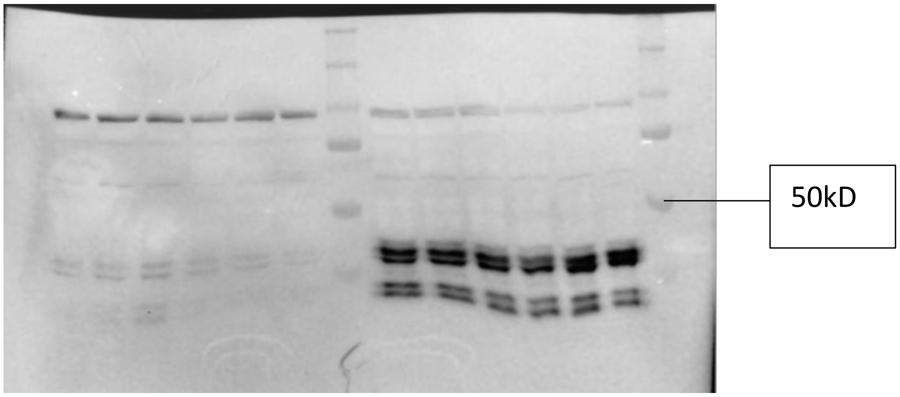

Figure 2, Source Data 1. Original membranes corresponding to Figure 2 C. BioRad protein ladder was used. Molecular weight for ACSS2 (78kD) and SIRT2 (43kD) are labelled.
