# Supplementary material for: SIRT2-mediated ACSS2 K271 deacetylation suppresses lipogenesis under nutrient stress
Source: eLife. 2025 May 7;13:RP97019. doi: 10.7554/eLife.97019 (PMC12058118; doi:10.7554/eLife.97019)
Supplement: Figure 2—figure supplement 8—source data 1. [file elife-97019-fig2-figsupp8-data1.zip › Figure 2-figure supplement 8, Source Data 1/Figure 2-figure supplement 8, Source Data 1. .pdf]

AcK IP (IB:Flag)

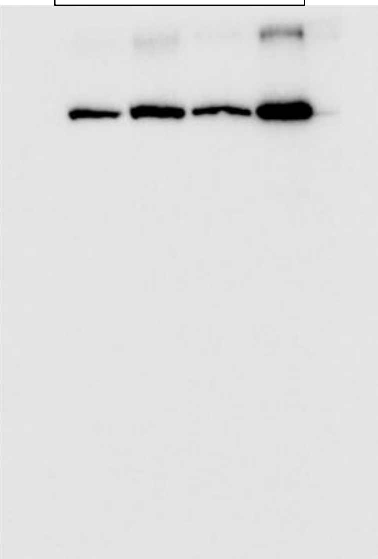

Input (IB:Flag)

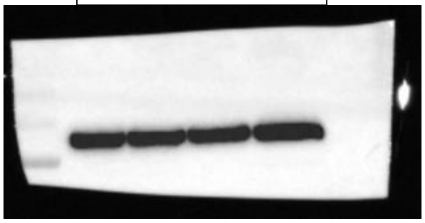

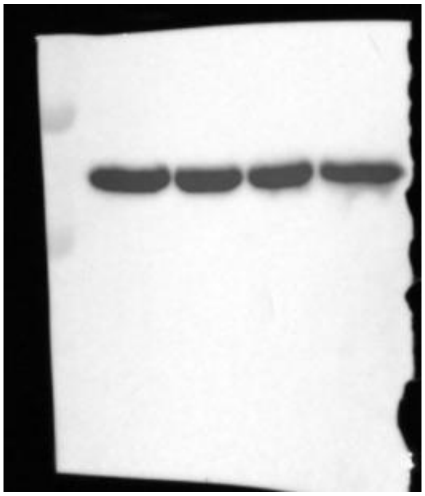

Input (IB:Actin)

Input (IB:SIRT2)

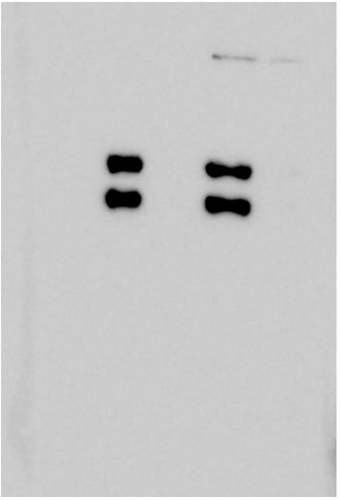

Figure 2-figure supplement 8, Source Data 1. Original membranes corresponding to Figure 2 supplement 8.
