# Supplementary material for: SIRT2-mediated ACSS2 K271 deacetylation suppresses lipogenesis under nutrient stress
Source: eLife. 2025 May 7;13:RP97019. doi: 10.7554/eLife.97019 (PMC12058118; doi:10.7554/eLife.97019)
Supplement: Figure 3—figure supplement 2—source data 1. [file elife-97019-fig3-figsupp2-data1.zip › Figure 3-figure supplement 2, Source Data 1/Figure 3-figure supplement 2, Source Data 1.pdf]

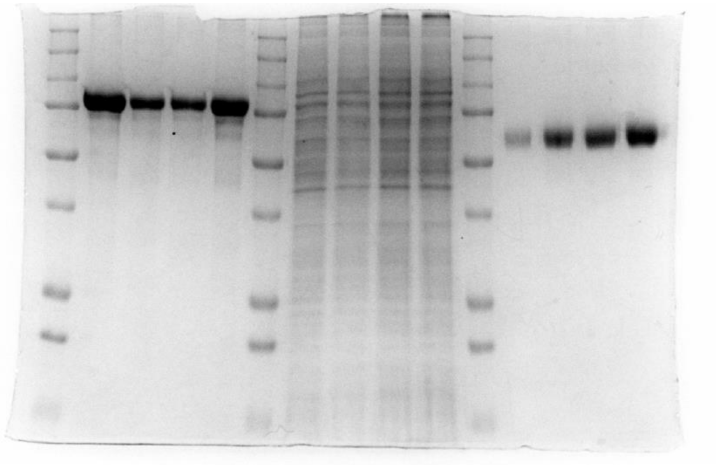

CBB

Figure 3-figure supplement 2, Source Data 1. Original membranes corresponding to Figure 3 supplement 2.
