# Supplementary material for: SIRT2-mediated ACSS2 K271 deacetylation suppresses lipogenesis under nutrient stress
Source: eLife. 2025 May 7;13:RP97019. doi: 10.7554/eLife.97019 (PMC12058118; doi:10.7554/eLife.97019)
Supplement: Supplementary file 1. [file elife-97019-supp1.docx]

| Annotated sequence | modifications | Modifications in master proteins | Abundance ratio: (SIRT2 KD/SIRT2 control | Abundances (grouped): SIRT2 KD | Abundances (grouped): SIRT2 control |
| --- | --- | --- | --- | --- | --- |
| [R].KIAQNDHDLGDMST VADPSVISHLFSHR.[C] | 1xAcetyl [K1]; 1xOxidation (M12) | Q9NR19 1xAcetyl [K669] |  |  |  |
| [R].LLMKFGDEPVTK.[H] | 1xAcetyl [K4]; 1xOxidation (M3) | Q9NR19 1xAcetyl [K418] | 0.959 | 97.9 | 102.1 |
| [R].AELGMGDSTSQSPP IKR.[S] | 1xAcetyl [K16]; | Q9NR19 1xAcetyl [K271] | 2.433 | 141.7 | 58.3 |
